# SUV39H2-mediated NCOA4 methylation controls ferritinophagy and ferroptosis in triple-negative breast cancer

**DOI:** 10.1038/s41419-026-09008-1

**Published:** 2026-07-16

**Authors:** Lingxia Liu, Xinyun Pei, Ding Li, Ying Huo, Zhaoting Wang, Qiu Zhang, Yuan Yue, Bao Deng, Minmin Yu, Tong Xu, Lei Zhong, Xianhui Ruan, Xichuan Li

**Affiliations:** 1 Tianjin Key Laboratory of Animal and Plant Resistance, College of Life Sciences, Tianjin Normal University, Tianjin, China; 2 Department of Clinical Laboratory, Tianjin Medical University Cancer Institute & Hospital, National Clinical Research Center for Cancer, Key Laboratory of Breast Cancer Prevention and Therapy, Tianjin Medical University, Ministry of Education, Tianjin’s Clinical Research Center for Cancer, Key Laboratory of Cancer Prevention and Therapy, Tianjin, China; 3 Department of Breast Surgery, Sixth Affiliated Hospital of Harbin Medical University, Harbin, China; 4 Department of Pathology, Affiliated Hospital of Shandong University of Traditional Chinese Medicine, Jinan, Shandong China; 5 Department of Thyroid and Neck Tumor, National Clinical Research Center for Cancer, Key Laboratory of Cancer Prevention and Therapy, Tianjin’s Clinical Research Center for Cancer, Tianjin Medical University Cancer Institute and Hospital, Tianjin, China

**Keywords:** Breast cancer, Autophagy, Methylation

## Abstract

Triple-negative breast cancer (TNBC) exhibits iron homeostasis that supports tumor growth and proliferation, yet the regulatory mechanisms controlling iron flux remain poorly defined. Here, we identify a ferritinophagic cargo receptor NCOA4 as a novel substrate of the lysine methyltransferase SUV39H2, uncovering a previously unrecognized mechanism that regulates ferritinophagy and ferroptosis. SUV39H2 directly binds and mono-methylates NCOA4 at lysine 356, a modification that reduces NCOA4 stability. Mechanistically, K356 methylation enhances NCOA4 interaction with the E3 ligase HERC2, promoting its ubiquitination and proteasomal degradation. This degradation increases FTH1 stability, suppresses ferritinophagic flux, limits iron release, maintains the high-risk iron homeostasis and ferroptosis resistance, ultimately promoting tumor proliferation and chemoresistance. Conversely, genetic or pharmacologic inhibition of SUV39H2 (OTS186935) inhibit NCOA4 methylation, stabilizes NCOA4 protein, enhances ferritinophagy, and triggers ferroptosis. Furthermore, SUV39H2 inhibition sensitizes TNBC cells to chemotherapy in vitro and in vivo, indicating OTS186935 treatment is a feasible therapeutic strategy. Collectively, the SUV39H2-NCOA4-HERC2 axis as a critical regulatory pathway in iron metabolism and ferroptosis, and highlight inhibition of NCOA4 K356 methylation as a promising therapeutic target in TNBC.

**Figure:**
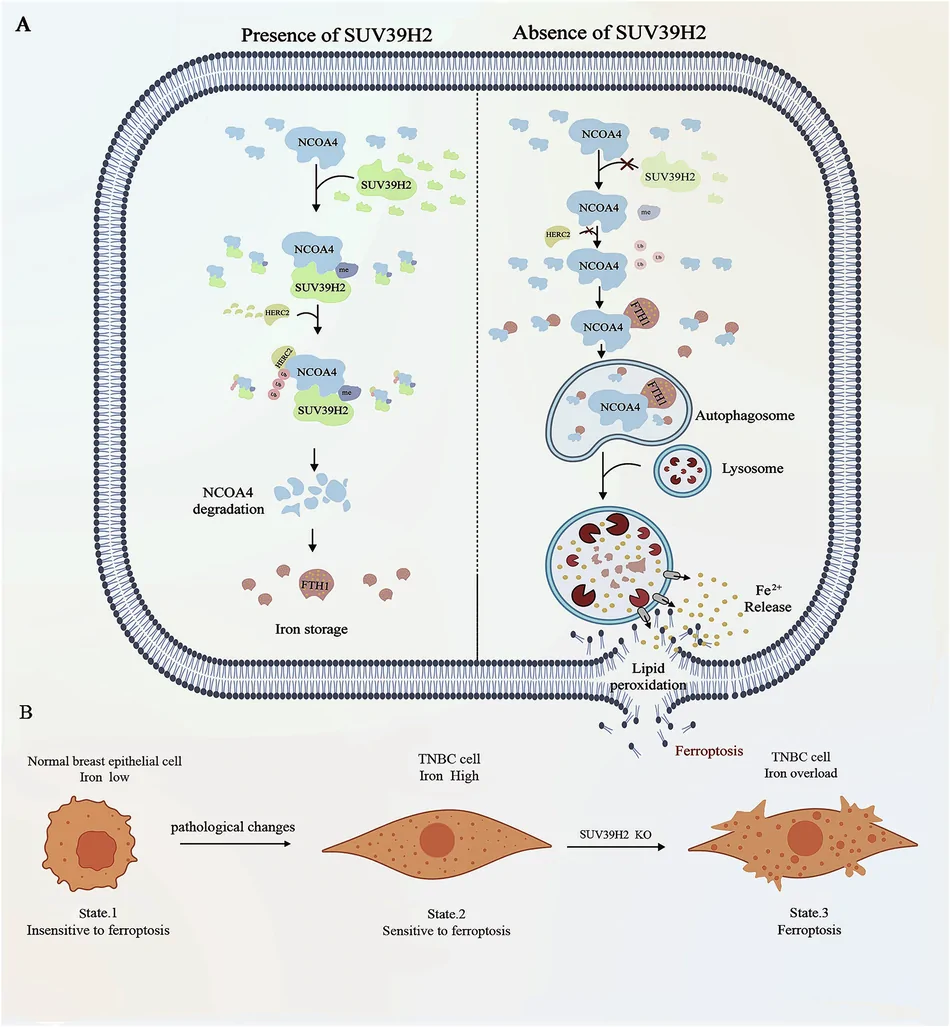
**The mechanistic scheme of SUV39H2 depletion to facilitate ferroptosis in TNBC. A** SUV39H2 binds and methylates NCOA4, which enhanced the interaction between NCOA4 and the E3 ubiquitin ligase HERC2, leading to NCOA4 ubiquitination and proteasomal degradation. As a result, iron metabolism was reprogramed via enhancing ferritinophagy, leading to an increase in the level of iron, ultimately triggering ferroptosis. **B** Cell-state transitions induced by SUV39H2.

**The mechanistic scheme of SUV39H2 depletion to facilitate ferroptosis in TNBC. A** SUV39H2 binds and methylates NCOA4, which enhanced the interaction between NCOA4 and the E3 ubiquitin ligase HERC2, leading to NCOA4 ubiquitination and proteasomal degradation. As a result, iron metabolism was reprogramed via enhancing ferritinophagy, leading to an increase in the level of iron, ultimately triggering ferroptosis. **B** Cell-state transitions induced by SUV39H2.

## Highlights

- NCOA4^low^-FTH1^high^ ferritinophagy axis contributes to maintaining iron homeostasis in TNBC.
- SUV39H2-mediated NCOA4 methylation facilitates HERC2-mediated ubiquitination and proteasomal degradation.
- Inhibition of NCOA4 K356me1 enhances ferritinophagic flux, disturbs iron homeostasis and triggers ferroptosis.
- OTS186935 induces ferroptosis in TNBC by targeting NCOA4 K356 methylation.

## Introduction

Iron is an essential trace element that supports numerous physiological processes in mammals, including energy metabolism, oxygen transport, DNA synthesis, and enzymatic activity [1]. Owing to its redox flexibility, iron cycles between ferrous (Fe^2+^) and ferric (Fe^3+^) states, enabling it to participate in various biochemical reactions [2]. However, the iron level is tightly regulated, as both iron deficiency and iron overload can lead to pathological conditions, including cancer [3–5]. Tumor cells usually exhibit abnormal iron metabolism, which is characterized by increased intracellular iron levels or “iron addiction” [6, 7]. Beyond acting as a nutrient, excess iron catalyzes the formation of ROS via the Fenton reaction, promoting genomic instability, lipid peroxidation, and ferroptosis, indicating tumor cells have a high-risk iron homeostasis [8–10].

Ferritinophagy, a selective form of autophagy mediated by the cargo receptor NCOA4, is thought to play a key role in maintaining intracellular iron homeostasis [11]. NCOA4 recognizes ferritin heavy chain (FTH1) and delivers ferritin to autophagosomes for degradation, leading to iron release in lysosomes [11–15]. This process regulates systemic and cellular iron availability and is increasingly recognized as a modulator of tumorigenesis [16–18]. Given this role of ferritinophagy in regulating iron homeostasis, enhancing NCOA4-mediated ferritinophagic flux increases vulnerability to ferroptosis [19–21]. Thus, a critical gap remains in understanding the molecular mechanisms by which ferritinophagy maintains intracellular high-risk iron levels without triggering ferroptosis.

Protein post-translational modifications (PTMs) are crucial enzymatic catalytic reactions that covalently regulate protein conformation, stability and cellular activities [22]. Accumulating evidence suggests that ferroptosis and ferritinophagy are tightly regulated by multiple PTMs, including methylation [23–26], phosphorylation [27–29], acetylation [30, 31], and ubiquitination [32]. Among these, protein lysine methylation has emerged as a key regulator of ferroptotic pathways [33]. Histone lysine methylation, such as H3K4me1, H3K4me3, and H3K9me3, has been shown to modulate ferroptosis [34–37]. Given that ferroptosis and ferritinophagy are regulated by many non-histone proteins, such as NCOA4, transferrin, and FTH1, it is highly plausible that lysine methylation of non-histone proteins may also play a significant role in these processes. SUV39H2, also a lysine methyltransferase, methylates histone H3K9 and non-histone proteins [38–40] and has been implicated in cancer proliferation and metastasis [41]. However, its specific role in regulating ferritinophagy and ferroptosis remains to be fully elucidated.

In this study, we identify a link between nonhistone lysine methylation and iron metabolism that modulates ferroptotic susceptibility. Mechanistically, SUV39H2 methylates NCOA4 at K356, promoting its degradation via enhanced HERC2 binding. This degradation suppresses ferritinophagy and ferroptosis, thereby contributing to tumor growth and chemoresistance. Our findings reveal an epigenetic mechanism by which SUV39H2 deficiency disrupts iron homeostasis and enhances ferroptotic susceptibility in TNBC.

## Results

### NCOA4^low^-FTH1^high^ ferritinophagy axis maintains iron homeostasis in TNBC

**Fig. 1:**
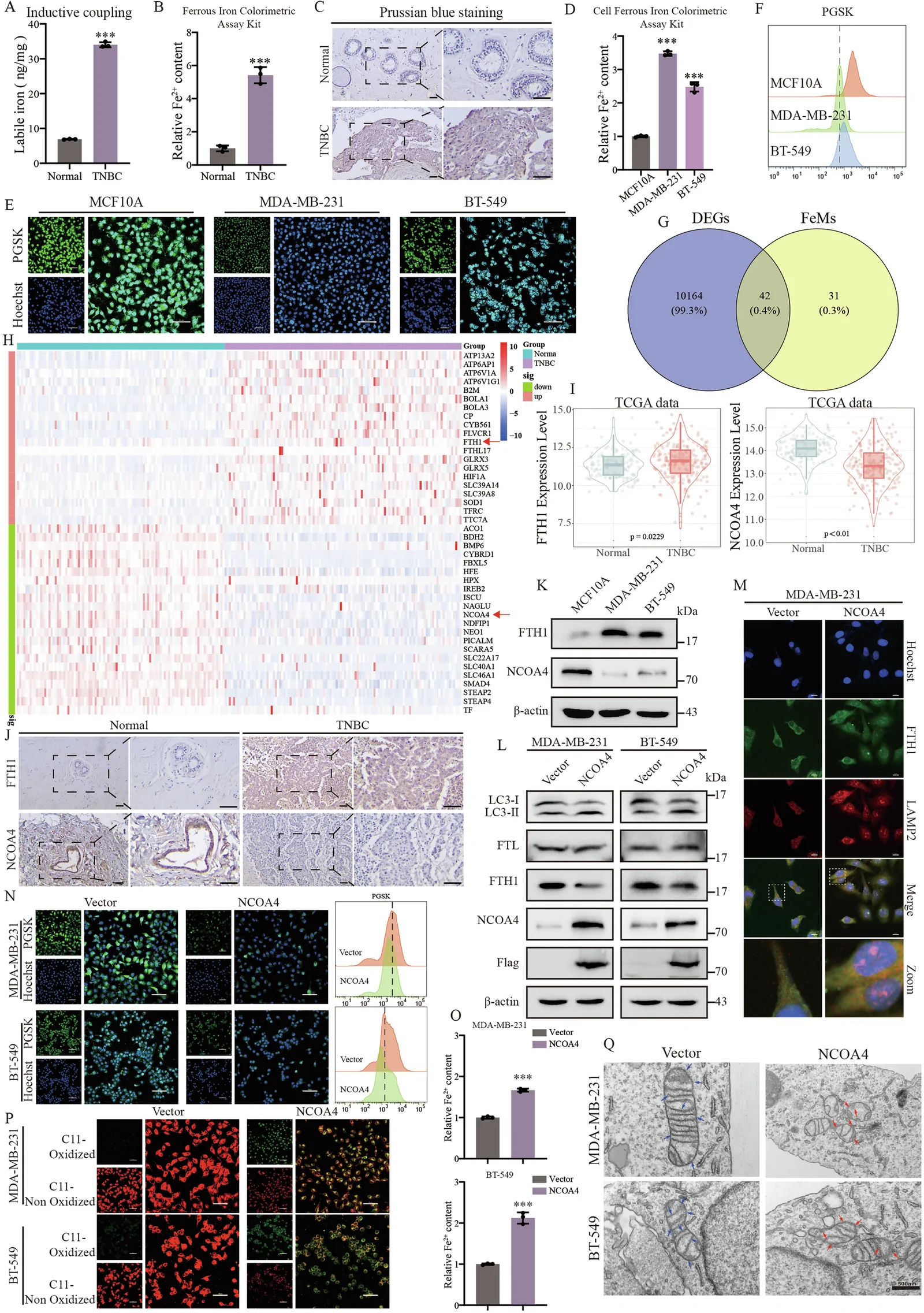
The NCOA4^low^-FTH1^high^ ferritinophagy axis maintains iron homeostasis in TNBC. **A** The iron content analyzed by ICP-OES in TNBC tissues and adjacent normal tissues. **B** Fe²⁺ levels measured by iron assay kit in TNBC tissues and adjacent normal tissues. **C** Prussian blue staining of the iron levels in TNBC tissues. Scale bar, 100 μm. **D** Fe²⁺ levels using iron assay kit in MCF10A, MDA-MB-231 and BT-549. **E** Fe²⁺ levels detected using PGSK fluorescent probe in MCF10A and TNBC cells. Scale bar, 100 μm. **F** Flow cytometry analysis of Fe²⁺ levels in TNBC cells. **G** Venn diagram showing overlapping genes between TNBC vs. adjacent differentially expressed genes (DEGs) and FeMs. **H** Heatmap of differentially expressed FeMs in TNBC vs. adjacent normal tissues. **I** Box plots showing differential expression levels of FTH1 and NCOA4. **J** Immunohistochemical staining of FTH1 and NCOA4 expression in TNBC tissues and adjacent normal tissues. Scale bar, 100 μm. **K** Western blot analysis of FTH1 and NCOA4 protein levels in MCF10A, MDA-MB-231 and BT-549. **L** Western blot analysis of LC3-I, LC3-II, FTL, and FTH1 protein levels in NCOA4-overexpressing cells. **M** Colocalization of endogenous FTH1 (green signal) and LAMP2 (red signal). Scale bar, 10 μm. **N** Fe²⁺ levels in NCOA4-overexpressing cells detected using PGSK probe. Representative images (left) and flow cytometry analysis (right). Scale bar, 100 μm. **O** Fe²⁺ levels measured by iron assay kit in NCOA4-overexpressing cells. **P** Lipid ROS levels measured using C11 fluorescent probe. Green: oxidized lipids; red: non-oxidized lipids. Representative images (left) and flow cytometry analysis (right). Scale bar, 100 μm. **Q** TEM images of NCOA4-overexpressing cells. Blue: normal mitochondria; red: damaged mitochondria. Scale bar, 500 nm. Data were presented as mean ± SD. Comparisons were made using one-way ANOVA with Tukey’s test; **p < 0.01, ***p < 0.001.

TNBC is characterized by aberrant iron and lipid metabolism, with elevated intracellular iron levels contributing to tumor progression [42]. To validate the presence of high iron accumulation in TNBC, we first measured the iron concentrations in TNBC tissues and adjacent normal tissues using ICP-OES (inductively coupled plasma optical emission spectrometry), colorimetric assays, and Prussian blue staining, which confirmed significantly increased iron levels in TNBC (Fig. 1A–C). Consistently, TNBC cell lines (MDA-MB-231 and BT-549) exhibited higher iron levels than non-tumorigenic mammary epithelial MCF10A cells (Fig. 1D–F and Fig. S1A). We hypothesized that elevated iron level in TNBC might result from altered expression of iron metabolism-related genes (FeMs) (Table S1). Analysis of The Cancer Genome Atlas (TCGA) data identified the expression levels of 42 FeMs in TNBC and normal controls varied significantly (Fig. 1G, H). Given that NCOA4-mediated ferritinophagy regulates intracellular iron homeostasis by directing ferritin degradation and iron release based on cellular demand [11], the NCOA4 and FTH1 expressive status may play a critical role in sustaining iron homeostasis in TNBC. To prove this hypothesis, we sought to investigate NCOA4 and FTH1 expressive levels in TNBC tissues and cells, and the results showed that FTH1 as significantly upregulated and NCOA4 as downregulated in TNBC compared with normal tissues (Fig. 1H–J), as well as by Western blot analyses in cell lines (Fig. 1K). Thus, the NCOA4^low^-FTH1^high^ ferritinophagy axis correlates with elevated iron homeostasis in TNBC.

To further determine whether disrupting this axis could enhance ferritinophagy, thereby inducing ferroptosis, we first stably overexpressed NCOA4 in MDA-MB-231 and BT-549 cells (Fig. S1B). NCOA4 overexpression led to decreased FTH1 protein levels and increased the expression of autophagic marker LC3-II, which was further enhanced by chloroquine (CQ) treatment (Fig. 1L and Fig. S1C). Subsequently, we performed tandem RFP-GFP-LC3 analysis, and the results showed that NCOA4 significantly increased the number of red puncta (Fig. S1D, E). Notably, the addition of CQ induced a more pronounced increase in yellow puncta accumulation, indicating that NCOA4 promoted autophagic flux (Fig. S1D, E). This was accompanied by increased co-localization of FTH1 with LAMP2 (Fig. 1M) and elevated intracellular iron levels (Fig. 1N, O and Fig. S1F), ultimately promoted ferritinophagy. We next evaluated whether ferritinophagy flux induces ferroptosis. Indeed, the overexpressed NCOA4 led to a significantly increase in ferroptotic biomarkers, including enhanced ROS (Figure S1G-I), elevated lipid ROS (Fig. 1P and Fig. S1J, K), and disrupted mitochondrial membrane potential (MMP) (Figure S1L), accompanied by condensed mitochondrial membranes and disrupted cristae (Fig. 1Q). Hence, the NCOA4^low^-FTH1^high^ ferritinophagy axis contributes to maintaining iron homeostasis in TNBC.

### NCOA4 is methylated by SUV39H2 at lysine 356

**Fig. 2:**
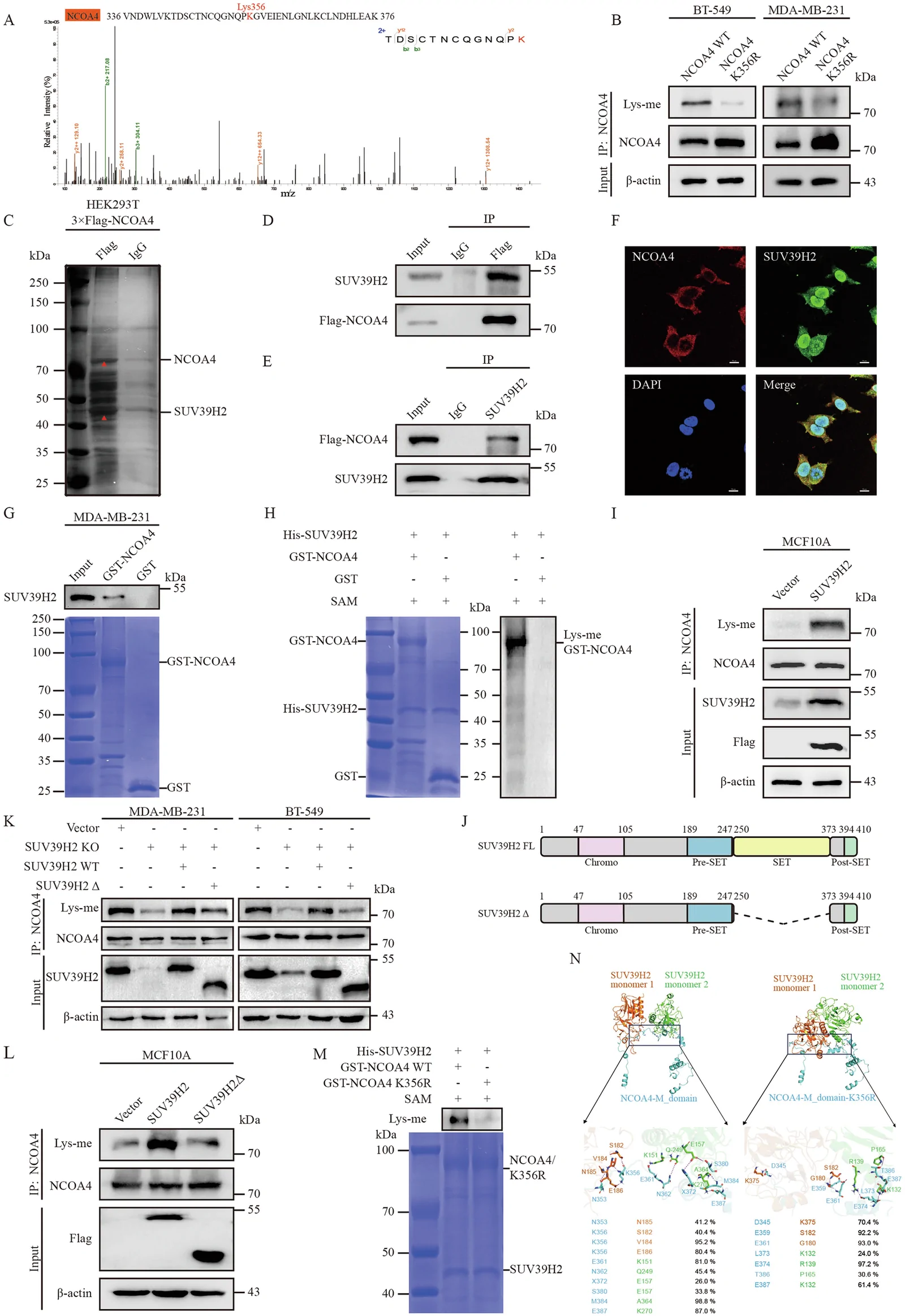
SUV39H2 catalyzes K356 methylation of NCOA4. **A** Identification of mono-methylated K356 of 3×Flag-NCOA4. Immunoprecipitation was performed using anti-Flag antibody in HEK-293T-NCOA4 cells, followed by liquid chromatography coupled with tandem mass spectrometry (LC-MS/MS) analysis. **B** Immunoprecipitation assay detecting methylation levels of NCOA4 WT and K356R mutant in MDA-MB-231 and BT-549 cell lines. **C** Mass spectrometry analysis of the purified 3×Flag-NCOA4 protein complex from the HEK-293T cells by anti-Flag immunoaffinity chromatography. The silver staining shows the location of 3×Flag-NCOA4 and its associated protein SUV39H2. **D**, **E** Western blot analyses of mutual co-immunoprecipitation of SUV39H2 and NCOA4. **F** Immunofluorescence showing co-localization of SUV39H2 (green) and 3×Flag-NCOA4 (red). Nuclei stained with DAPI (blue). Scale bar, 10 μm. **G** GST-pull down assay showing direct interaction between GST-NCOA4 and SUV39H2. GST and GST-NCOA4 proteins are shown by Coomassie Brilliant Blue staining. **H** Identification of recombinant NCOA4 as a substrate for SUV39H2 in vitro. Left, Coomassie staining. Right, Western blot. **I** Immunoprecipitation assay detecting the increased methylation level of NCOA4 in SUV39H2-overexpressing MCF10A cells. **J** Schematic diagram of full-length SUV39H2 and its truncated derivatives (SUV39H2Δ). **K** The level of NCOA4 methylation in MDA-MB-231\BT549-SUV39H2 KO cells overexpressing SUV39H2 or SUV39H2Δ. **L** IP assay evaluating the level of NCOA4 methylation in MCF10A cells overexpressing SUV39H2 or SUV39H2Δ. **M** In vitro methylation assays of NCOA4 K356R and NCOA4 WT. Western blot (top panel) and Coomassie brilliant blue staining (bottom panel). **N** Display hydrogen interactions between the SUV39H2 dimer and NCOA4-M_domain in the SUV39H2 dimer/NCOA4-M_domain-Wild and the SUV39H2 dimer/NCOA4-M_domain-K356R system. SUV39H2 monomer 1, SUV39H2 monomer 2, and NCOA4-M-domain were represented as orange, green, and cyan cartoons, respectively. The residues involved in hydrogen bonding between the proteins were shown as sticks in the corresponding colors. The table below lists the proportion of time that each hydrogen bond is present during the final 50 ns of the simulations.

Previous studies have shown that NCOA4 stability can be regulated by ubiquitination and arginine methylation [43, 44], but whether it is subject to other post-translational modifications has not been explored. To identify potential modification types and sites, we performed mass spectrometry on purified NCOA4 protein and identified mono-methylation at lysine 356 (K356me1) (Fig. 2A). Sequence alignment showed that K356 is highly conserved across species (Fig. S1M). As expected, mutating K356 to arginine (K356R) substantially reduced the lysine methylation (Fig. 2B). To identify potential modifying enzymes, we performed mass spectrometry on the 3×Flag-NCOA4 protein complex and the lysine methyltransferase SUV39H2 was identified as a putative NCOA4-binding protein (Fig. 2C). This interaction was further supported by AlphaFold-based structural modeling (Figure S1N), co-immunoprecipitation (Co-IP) assays (Fig. 2D, E) and immunofluorescence assays (Fig. 2F). Direct binding was also validated by GST pull-down using recombinant NCOA4 and MDA-MB-231 cell lysates (Fig. 2G). To determine whether SUV39H2 methylates NCOA4, we performed an in vitro methylation assay, which revealed that SUV39H2 catalyzed methylation of NCOA4 (Fig. 2H). Overexpression of SUV39H2 in MCF10A cells significantly increased lysine methylation of NCOA4 (Fig. 2I), while knockout of SUV39H2 using CRISPR/Cas9 in MDA-MB-231 and BT-549 cells markedly decreased NCOA4 methylation (Fig. 2J, K). Additionally, a catalytically inactive mutant of SUV39H2 failed to promote NCOA4 methylation (Fig. 2K, L). Despite high sequence conservation between SUV39H1 and SUV39H2 [39], SUV39H1 knockout did not reduce NCOA4 methylation (Fig. S1O), establishing SUV39H2 as the primary methyltransferase responsible for NCOA4 methylation.

To further validate K356 as a direct substrate site of SUV39H2, we generated a K356R mutant of GST-NCOA4 for use in vitro methylation assays. As expected, the K356R mutation substantially reduced SUV39H2-mediated methylation (Fig. 2M). Meanwhile, molecular modeling using the crystal structure of NCOA4 suggested that K356 was located within a core domain predicted to interface with SUV39H2 (Fig. S1P), suggesting that the K356R mutation likely impairs NCOA4-SUV39H2 binding. To prove this hypothesis, we constructed SUV39H2 dimer/NCOA4-M_domain-Wild (K356-in-M_domain) and SUV39H2 dimer/NCOA4-M_domain-K356R simulation systems. Both systems reached structural stable after ~120 ns of simulation and RMSF analysis showed that the K356R mutation markedly increased local flexibility of the NCOA4-M_domain (Fig. S1Q, R). Notably, the mutation also destabilized the SUV39H2 dimer, as reflected by elevated RMSF values (Fig. S1S, T). Hydrogen-bond analysis showed that K356 in the NCOA4-M domain forms stable hydrogen with S182, V184, and E186 in SUV39H2 in the wild-type system, whereas the K356R mutation disrupted these interactions, resulting in reduced complex stability (Fig. 2N). The destabilizing effect was further corroborated by immunoprecipitation assays, which showed that the NCOA4 K356R mutation impaired its binding to SUV39H2 (Fig. S1U). Thus, K356 serves as both a critical methylation site and a key structural determinant for the NCOA4-SUV39H2 interaction.

### SUV39H2 promotes NCOA4 degradation via the ubiquitin-proteasome system

**Fig. 3:**
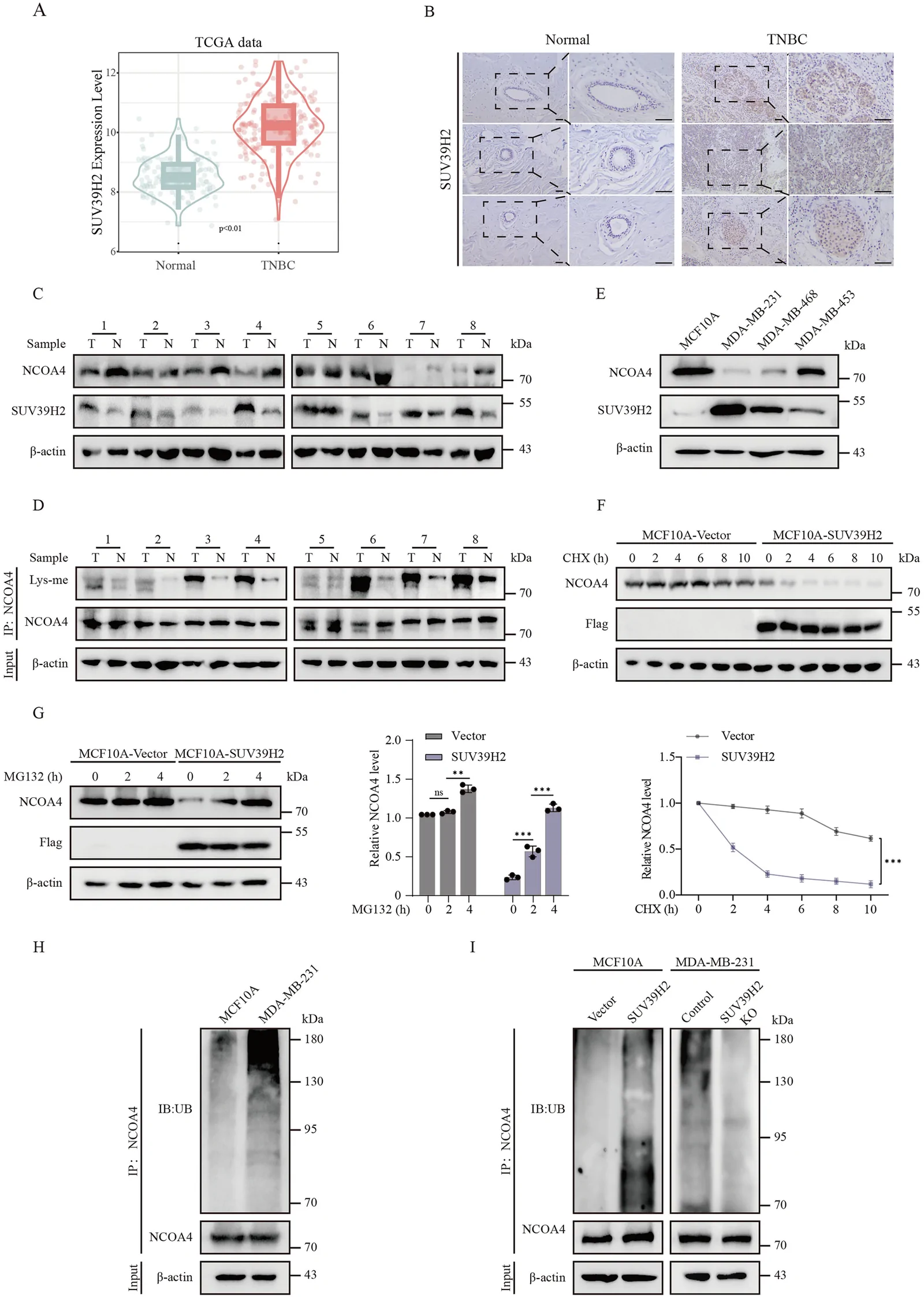
SUV39H2 drives NCOA4 degradation. **A** Box plots showing differential expression of SUV39H2 in TNBC vs. adjacent normal tissues based on TCGA data. **B** Immunohistochemical staining of SUV39H2 expression in TNBC tissues and adjacent normal tissues. Scale bar, 100 μm. **C** Western blot analysis of NCOA4 and SUV39H2 protein levels in TNBC tissues and adjacent normal tissues. **D** Immunoprecipitation assay detecting NCOA4 methylation levels in TNBC tissues and adjacent normal tissues. **E** Western blot analysis of NCOA4 and SUV39H2 protein levels in MCF10A, MDA-MB-231, MDA-MB-468, and MDA-MB-453 cells. **F** Western blot analysis of NCOA4 protein levels in SUV39H2-overexpressing MCF10A cells treated with CHX (100 μg/mL). **G** Western blot analysis of NCOA4 protein levels in SUV39H2-overexpressing MCF10A cells treated with MG132 (10 μM). **H** Immunoprecipitation assay detecting the ubiquitination levels of NCOA4 in MCF10A and MDA-MB-231 cells. **I** Immunoprecipitation assay detecting the ubiquitination levels of NCOA4 in SUV39H2-overexpressing MCF10A cells and SUV39H2-knockout MDA-MB-231 cells. Comparisons were assessed using a two-sided Wilcoxon rank-sum test, ***p < 0.001.

An inverse relationship between SUV39H2 and NCOA4 expression was observed in TNBC tissues and cells. TCGA data analyses, GEO data analyses, immunohistochemistry, and western blot revealed that SUV39H2 expression was markedly higher in tumor tissues compared to adjacent normal tissues, whereas NCOA4 levels were lower (Fig. 3A–C and Fig. S2A). Consistent with this, tissues with high SUV39H2 expression also exhibited elevated NCOA4 lysine methylation and reduced NCOA4 protein levels (Fig. 3D). Following this, we performed survival analysis, and the result revealed that SUV39H2 expression alone was not significantly associated with patient prognosis (Fig. S2B). Notably, we identified a patient subgroup characterized by the SUV39H2^high^/NCOA4^low^ expression pattern, and this signature was correlated with worse clinical outcomes (Fig. S2C, D). We next compared SUV39H2 and NCOA4 protein levels across breast cell lines and found that TNBC cells expressed higher SUV39H2 and lower NCOA4 compared to MCF10A (Fig. 3E). These observations raised the possibility that SUV39H2 regulates NCOA4 protein stability. To this end, we treated MCF10A-SUV39H2 cells with cycloheximide (CHX) to block new protein synthesis or with the proteasome inhibitor MG132. CHX treatment accelerated NCOA4 degradation, while MG132 treatment prevented it (Fig. 3F, G), suggesting SUV39H2 promotes NCOA4 degradation.

Since protein degradation often occurs via ubiquitination, we next investigated whether SUV39H2 modulates NCOA4 ubiquitination. Indeed, NCOA4 showed higher ubiquitination levels in MDA-MB-231 cells relative to MCF10A cells (Fig. 3H). Moreover, SUV39H2 overexpression increased NCOA4 ubiquitination (Fig. 3I), whereas SUV39H2 knockout reduced it (Fig. 3I). Together, these results demonstrate that SUV39H2 promotes NCOA4 degradation via the ubiquitin–proteasome system.

### NCOA4 K356me1 facilitates HERC2-mediated ubiquitination of NCOA4

**Fig. 4:**
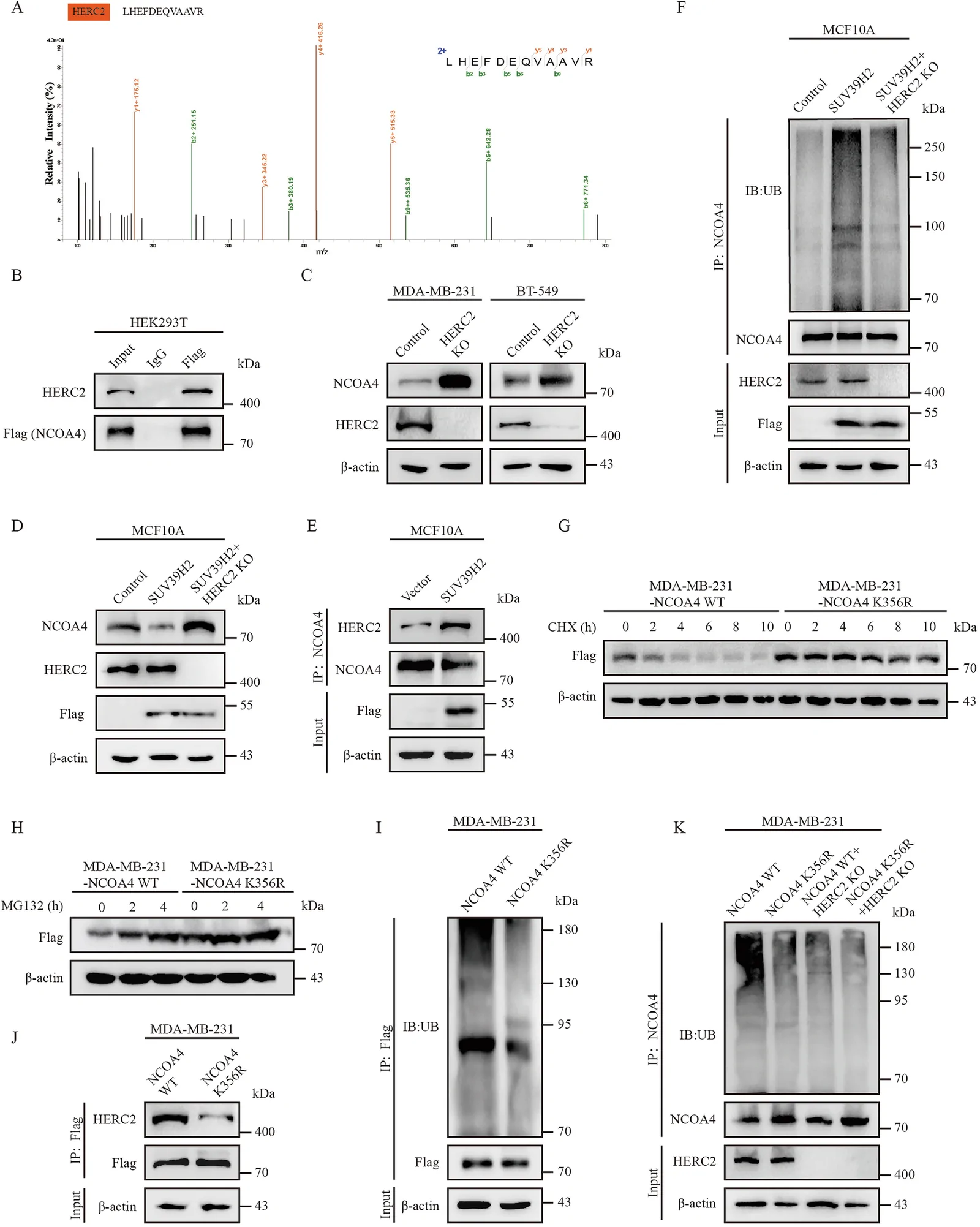
NCOA4 K356me1 promotes ubiquitin-proteasome degradation via enhancing interaction with HERC2. **A** The identification of HERC protein through mass spectrometry. **B** Co-immunoprecipitation of NCOA4 and HERC2 in NCOA4-overexpressing HEK-293T cells. **C** Western blot analysis of NCOA4 protein levels in HERC2-knockout MDA-MB-231 and BT-549 cells. **D** Western blot analyses of NCOA4 protein levels in MCF10A cells overexpressing SUV39H2 or co-overexpressing SUV39H2 and HERC2-knockout. **E** Immunoprecipitation assay detecting the binding ability of NCOA4 and HERC2 proteins in SUV39H2-overexpressing MCF10A cells. **F** Immunoprecipitation assay detecting ubiquitination levels of NCOA4 in MCF10A cells overexpressing SUV39H2 or co-overexpressing SUV39H2 and HERC2-knockout. **G** Western blot analysis of NCOA4 protein levels in CHX-treated MDA-MB-231 cells overexpressing NCOA4 WT or K356R mutant. **H** Western blot analysis of NCOA4 protein levels in MG132-treated MDA-MB-231 cells overexpressing NCOA4 WT or K356R mutant. **I** IP assay detecting ubiquitination levels of NCOA4 in MDA-MB-231 cells overexpressing NCOA4 WT or K356R mutant. **J** IP assay detecting HERC2 levels which was interacted with NCOA4 in MDA-MB-231 cells overexpressing NCOA4 WT or K356R mutant. **K** IP assay detecting ubiquitination levels of NCOA4 under combinatorial conditions of WT/K356R mutant and HERC2-knockout.

To further explore whether SUV39H2 facilitates the recruitment of ubiquitin ligases to NCOA4, we analyzed our LC-MS/MS data and identified HERC2, a known E3 ligase reported to interact with NCOA4[43], as a potential binding partner (Fig. 4A). Co-IP confirmed the in vivo interaction between HERC2 and NCOA4 (Fig. 4B). Meanwhile, HERC2 depletion led to increased NCOA4 protein levels (Fig. 4C), and similarly rescued NCOA4 expression in MCF10A-SUV39H2 cells (Fig. 4D). Moreover, SUV39H2 overexpression enhanced the interaction between HERC2 and NCOA4 (Fig. 4E), while HERC2 knockout reduced NCOA4 ubiquitination (Fig. 4F). Therefore, SUV39H2 facilitates NCOA4 degradation by promoting its ubiquitination via HERC2.

We next asked whether SUV39H2 promotes NCOA4 ubiquitination through K356 methylation. Supporting this, NCOA4 WT exhibited a shorter protein half-life than the K356R mutant (Fig. 4G), and MG132 treatment blocked the degradation (Fig. 4H). Consistently, NCOA4 WT showed markedly increased ubiquitination compared to K356R (Fig. 4I), indicating that methylation at K356 enhances protein degradation. Additionally, Co-IP revealed stronger interaction between HERC2 and NCOA4 WT than with K356R (Fig. 4J). Furthermore, HERC2 knockout decreased NCOA4 WT ubiquitination, while it had little effect on the K356R mutant (Fig. 4K). Thus, NCOA4 K356 methylation promotes its ubiquitination and degradation, in part by modulating its interaction with HERC2.

### Inhibition of NCOA4 K356me1 enhances ferritinophagic flux, disturbs iron homeostasis and triggers ferroptosis

**Fig. 5:**
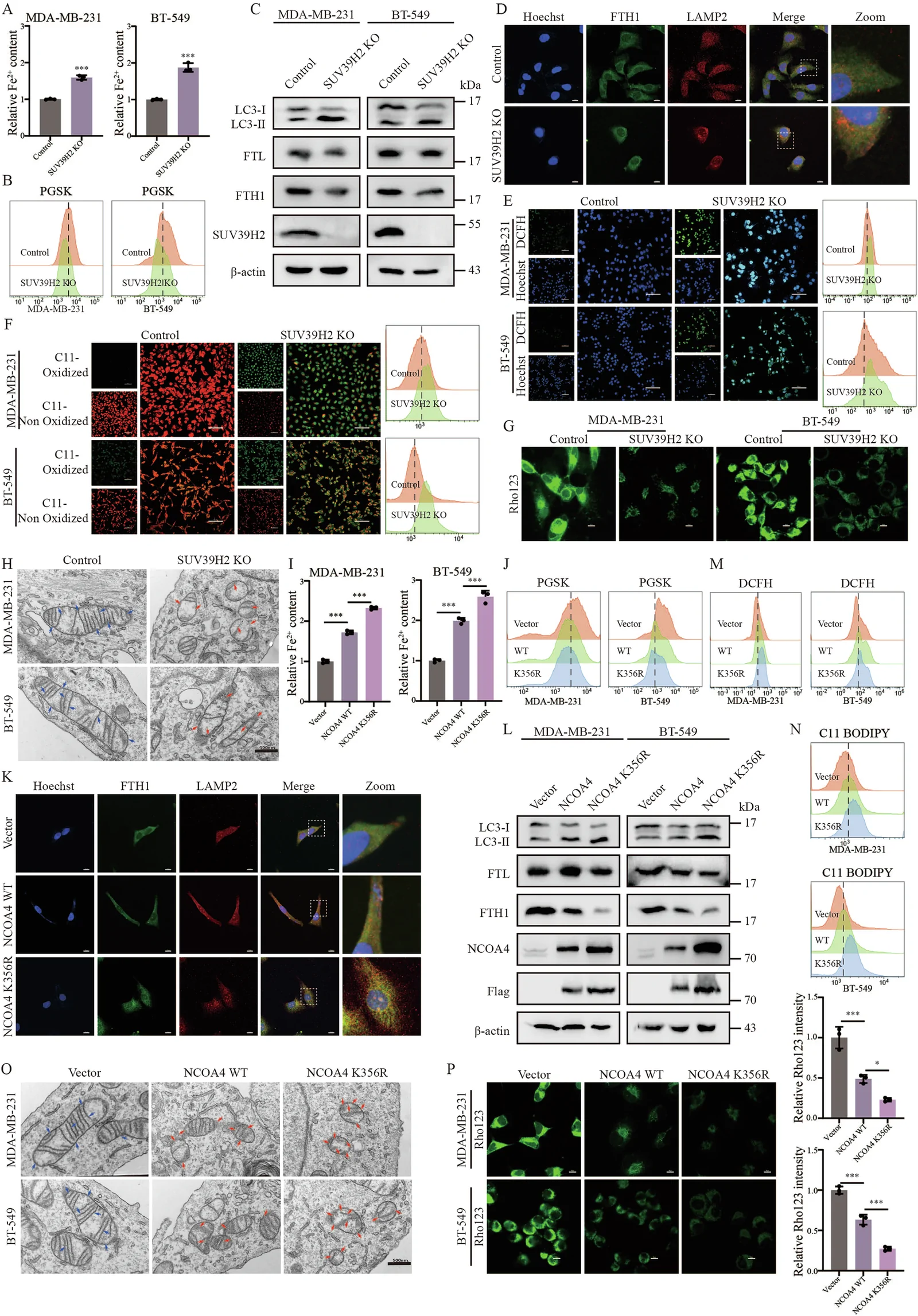
NCOA4 K356 methylation regulates ferritinophagy and ferroptosis in TNBC. **A** Fe²⁺ levels in SUV39H2-knockout cells measured by iron assay kit. **B** Flow cytometry analysis of Fe²⁺ levels in SUV39H2-KO cells. **C** Western blot analysis of LC3-I/II, FTL, and FTH1 protein levels in SUV39H2-knockout MDA-MB-231 cells and BT-549 cells. **D** Colocalization of endogenous FTH1 (green signal) and LAMP2 (red signal) in SUV39H2-KO MDA-MB-231 cells. Scale bar, 10 μm. **E** Representative images and flow cytometry analysis of ROS levels in SUV39H2-KO cells. Scale bar, 100 μm. **F** Representative images and flow cytometry analysis of lipid ROS levels in SUV39H2-KO cells. Scale bar, 100 μm. **G** MMP in SUV39H2-KO cells detected using Rhodamine 123 probe. Scale bar, 10 μm. **H** TEM images of SUV39H2-KO cells. Blue: normal mitochondria; red: damaged mitochondria. Scale bar, 500 nm. **I** Fe²⁺ levels measured by iron assay kit in NCOA4 WT or K356R mutant-overexpressing cells. **J** Flow cytometry analysis of Fe²⁺ levels in NCOA4 WT or K356R mutant-overexpressing cells. **K** Colocalization of endogenous FTH1 (green signal) and LAMP2 (red signal) in MDA-MB-231 cells overexpressing NCOA4 WT or K356R mutant. Scale bar, 10 μm. **L** Western blot analysis of LC3-I/II, FTL, and FTH1 protein levels in cells overexpressing NCOA4 WT or K356R mutant. **M** Flow cytometry analysis of ROS levels in NCOA4 WT or K356R mutant-overexpressing cells. **N** Flow cytometry analysis of lipid ROS levels in NCOA4 WT or K356R mutant-overexpressing cells. **O** TEM images of NCOA4 WT or K356R mutant-overexpressing cells. Blue: normal mitochondrial cristae; red: damaged mitochondrial cristae. Scale bar, 500 nm. **P** MMP in NCOA4-overexpressing cells detected using Rhodamine 123 probe. Scale bar, 10 μm. Data were presented as mean ± SD. Comparisons were made using one-way ANOVA with Tukey’s test; **p < 0.01, ***p < 0.001.

Given that SUV39H2 methylates NCOA4, we hypothesized that SUV39H2 might regulate ferritinophagy and ferroptosis. As expected, SUV39H2 knockout led to elevated intracellular iron levels (Fig. 5A, B and Fig. S2E), decreased FTH1 expression, and increased LC3-II levels (Fig. 5C and Fig. S2F). Consistently, the co-localization of endogenous FTH1 with LAMP2 (Fig. 5D) and the number of red puncta (Fig. S2G, H) were increased, further confirming the induction of ferritinophagy. We then assessed the effect of SUV39H2 deficiency on ferroptosis. Knockout of SUV39H2 resulted in increased cellular ROS (Fig. 5E and Fig. S2I), lipid ROS (Fig. 5F and Fig. S2J), a reduction in MMP (Fig. 5G and Fig. S2K), and typical ferroptotic mitochondrial changes (Fig. 5H). These findings indicate that SUV39H2 inhibition induces ferroptosis and ferritinophagy.

To test whether SUV39H2 deletion-induced ferroptosis is mediated by NCOA4, we knocked out NCOA4 in SUV39H2-deficient cells. The results showed that SUV39H2 loss elevated lipid ROS and labile iron, which were reversed by NCOA4 ablation, confirming that these phenotypes depend on NCOA4 (Fig. S2L–N). Having established this, we next investigated the functional role of SUV39H2-mediated NCOA4 K356 methylation in regulating ferritinophagy and ferroptosis. We found that NCOA4 K356R resulted in increased intracellular iron levels, co-localization of ferritin with lysosomes, and autolysosome formation compared to NCOA4 WT (Fig. 5I–K and Fig. S3A–C). Western blot analysis further revealed that the K356R mutation inhibited FTH1 expression and promoted the conversion of LC3-I to LC3-II (Fig. 5L and Fig. S3D), indicative of increased ferritinophagy. To assess ferroptosis, we measured relevant biomarkers and found that the K356R mutation significantly promoted ferroptotic cell death (Fig. 5M–P and Fig. S3E, F). Together, NCOA4 K356me1 suppresses ferritinophagy, thereby promoting ferroptosis resistance.

### Inhibition of NCOA4 K356me1 suppresses tumor growth and enhances chemosensitivity in vitro and in vivo

**Fig. 6:**
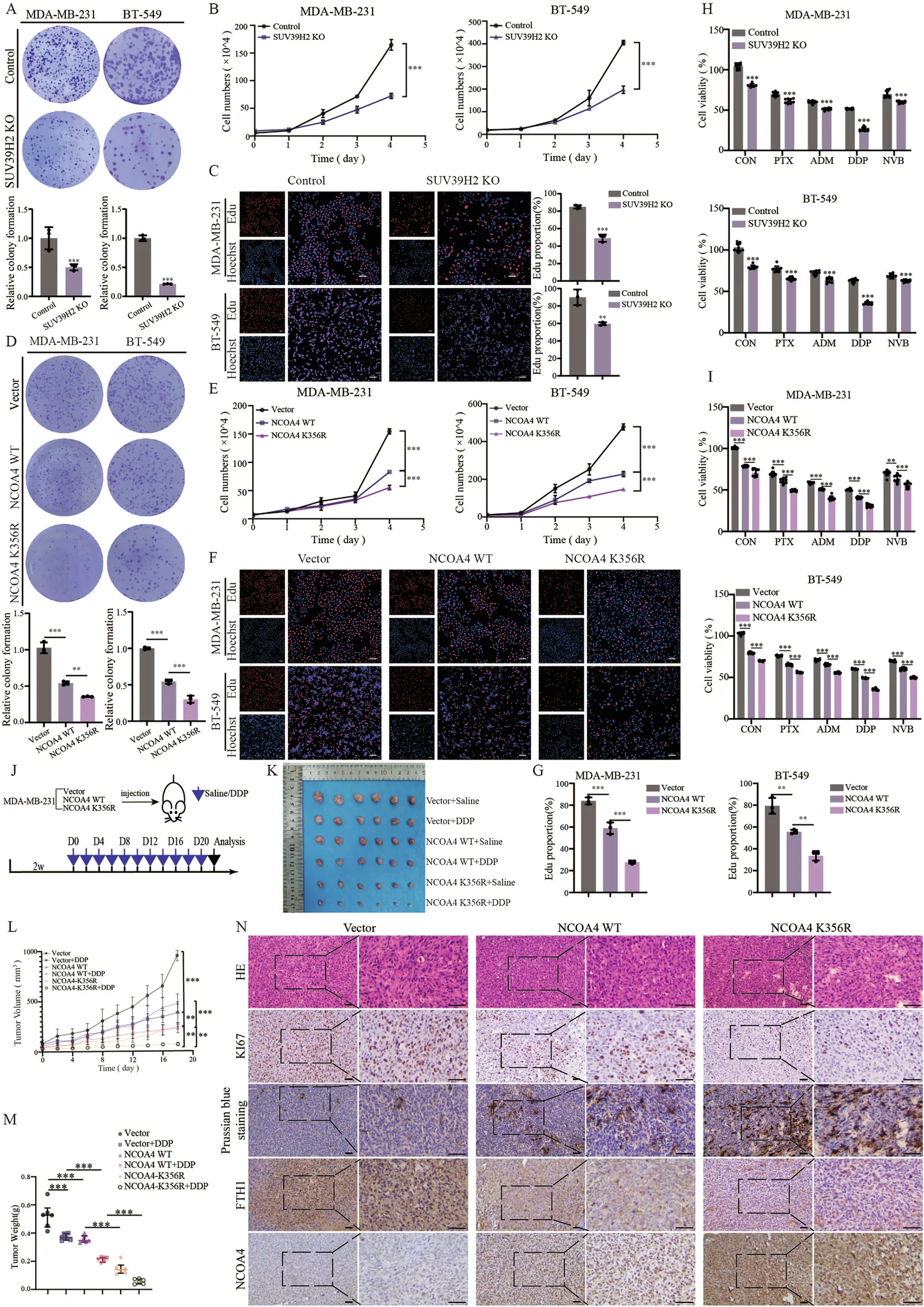
NCOA4 K356 methylation modulates malignant behaviors and chemotherapy sensitivity. **A** Representative images and quantitative analyses of colony formation assay in SUV39H2-KO MDA-MB-231 and BT-549 cells. **B** Growth curves of SUV39H2-KO MDA-MB-231 and BT-549 cells. **C** Representative images and quantitative analyses of EdU assay in SUV39H2-knockout MDA-MB-231 and BT-549 cells. Scale bar, 100 μm. **D** Representative images and quantitative analyses of colony formation assay in NCOA4 WT or K356R mutant-overexpressing MDA-MB-231 and BT-549 cells. **E** Growth curves of NCOA4 WT or K356R mutant-overexpressing MDA-MB-231 and BT-549 cells. **F** Representative images of EdU assay in NCOA4 WT/K356R overexpressed MDA-MB-231 and BT-549 cells. Scale bar, 100 μm. **G** Quantitative analyses of EdU assay in NCOA4 WT/K356R overexpressed MDA-MB-231 and BT-549 cells. **H** Cell viability of SUV39H2-knockout cells treated with PTX (1.5 nM), ADM (50 nM), DDP (10 μM) or NVB (2 nM). **I** Cell viability of NCOA4 WT/K356R-overexpressing cells treated with PTX, ADM, DDP, or NVB. **J** Schematic of xenograft models using MDA-MB-231 NCOA4 WT or K356R mutant cells with DDP (5 mg/kg) treatment. **K**–**M** Tumor images, volumes, and weights in treatment groups (Vector+Saline, Vector+DDP, NCOA4 WT+Saline, NCOA4 WT + DDP, NCOA4 K356R+Saline, NCOA4 K356R + DDP, mean of 6 mice). **N** HE staining, Prussian blue staining, and immunohistochemical staining of Ki67, FTH1, and NCOA4 in tumor tissues. Scale bar, 100 μm. Data are presented as mean ± SD. Comparisons were made using one-way ANOVA with Tukey’s test; **p < 0.01, ***p < 0.001.

Given our findings that NCOA4 overexpression suppresses TNBC cell proliferation and invasion (Fig. S4A–E), we next examined the functional role of SUV39H2 in TNBC cell growth and invasion. SUV39H2 knockout significantly reduced colony formation (Fig. 6A), cell proliferation (Fig. 6B, C), and cell migration and invasion (Fig. S4F, G). Similarly, expression of the NCOA4 K356R mutant—resistant to SUV39H2-mediated methylation—significantly impaired colony formation (Fig. 6D), growth (Fig. 6E–G), and invasive properties (Fig. S4H–J) compared to NCOA4 WT. Ferroptosis has been implicated in regulating chemotherapy resistance [45]. Therefore, we tested whether K356 methylation modulates chemosensitivity in TNBC. SUV39H2 knockout increased the sensitivity of MDA-MB-231 and BT-549 cells to paclitaxel (PTX), doxorubicin (ADM), cisplatin (DDP), and vinorelbine (NVB), with the most pronounced effect observed in response to DDP (Fig. 6H and Fig. S5A). We further explored the IC50 values of chemotherapeutic drugs using *pRRophetic* R software package. Notably, we found that the IC50 value of DDP and PTX was lower in TNBC patients with SUV39H2^low^/NCOA4^high^ expression (Fig. S5B), suggesting that the NCOA4 K356R mutation may also increase sensitivity to DDP. Consistently, cells overexpressing NCOA4 K356R displayed greater sensitivity to DDP compared to NCOA4 WT-expressing cells or cells treated with other chemotherapeutic agents (Fig. 6I).

To assess the in vivo relevance of NCOA4 K356 methylation in ferroptosis and chemotherapy response, we established a xenograft model using MDA-MB-231 cells stably expressing either NCOA4 WT or the methylation-resistant K356R mutant. And 2 weeks after cell inoculation, DDP was administered every 2 days for 3 weeks (Fig. 6J). Tumor volume and weight were significantly reduced in mice bearing K356R-expressing tumors (Fig. 6K–M). Immunohistochemical analysis confirmed that tumors expressing NCOA4 K356R exhibited reduced Ki67 staining, consistent with suppressed proliferation, and increased iron accumulation and decreased FTH1, consistent with enhanced ferroptosis (Fig. 6N). Notably, the combination of NCOA4 K356R expression and DDP treatment led to a synergistic inhibition of tumor growth, whereas K356R expression alone had a moderate effect (Fig. 6J–N). These findings underscore that methylation-mutant NCOA4 at K356 not only impairs tumor proliferation *via* ferroptosis but also sensitizes TNBC tumors to cisplatin chemotherapy in vivo.

### OTS186935 induces ferroptosis in TNBC by targeting NCOA4 K356 methylation

**Fig. 7:**
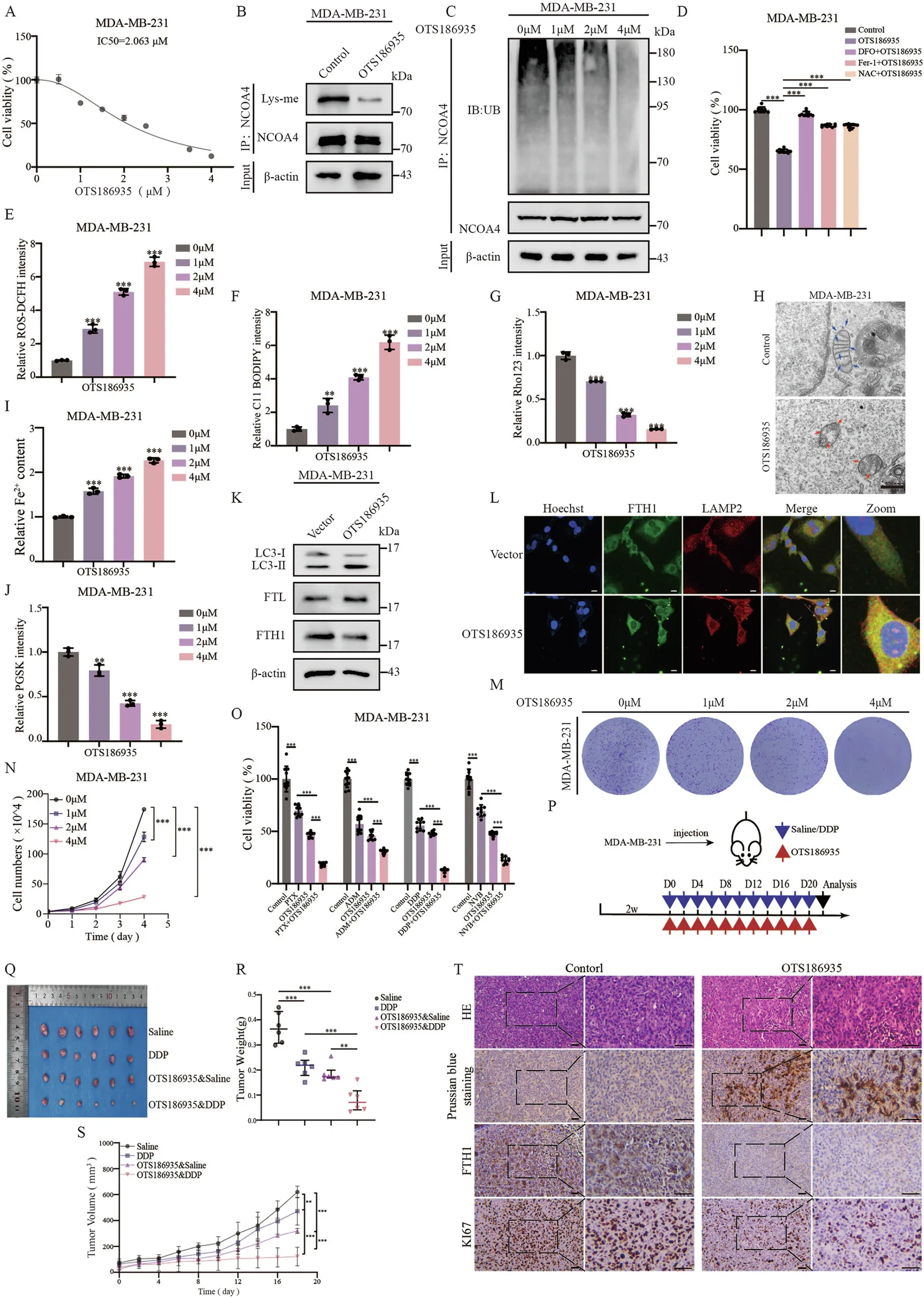
OTS186935 targets SUV39H2 to enhance ferritinophagy, ferroptosis and anti-TNBC effects. **A** CCK8 assay detecting the IC50 of OTS186935 in MDA-MB-231 cells**. B** Immunoprecipitation assay detecting NCOA4 methylation levels in OTS186935 (2 μM)-treated MDA-MB-231 cells. **C** Immunoprecipitation assay detecting NCOA4 ubiquitination levels under OTS186935 treatment. **D** The analyses of cell viability in MDA-MB-231 after OTS186935 treatment combined with ferroptosis inhibitors (DFO: 10 μM, Fer-1: 10 μM, NAC: 1 mM). **E**–**J** Quantitative analyses of ROS, lipid ROS, MMP, TEM, and Fe²⁺ levels in OTS186935-treated MDA-MB-231 cells. **K** Western blot analysis of LC3I/II, FTL, and FTH1 protein levels in OTS186935-treated MDA-MB-231 cells. **L** Colocalization of endogenous FTH1 (green signal) and LAMP2 (red signal) in OTS186935-treated MDA-MB-231 cells. **M** Representative images of colony formation assay in OTS186935-treated MDA-MB-231 cells. **N** Growth curves of OTS186935-treated MDA-MB-231 cells. **O** Cell viability of chemotherapeutic drugs combined with OTS186935. **P** Schematic of xenograft models following OTS186935 (25 mg/kg)/DDP (5 mg/kg) treatments. **Q**–**S** Tumor images, weights, and volumes in treatment groups (Control+Saline, Control+DDP, OTS186935+Saline, OTS186935 + DDP, mean of 6 mice). **T** HE staining, prussian blue staining, and immunohistochemical staining of FTH1 and Ki67 in tumor tissues. Scale bar, 100 μm. Data are presented as mean ± SD. Comparisons were made using one-way ANOVA with Tukey’s test; **p < 0.01, ***p < 0.001.

Having shown that SUV39H2-mediated methylation of NCOA4 at K356 suppresses ferritinophagy and promotes chemotherapy resistance in TNBC, we next tested whether OTS186935, a known SUV39H2 inhibitor[46–48], could restore ferroptosis and chemosensitivity. We performed the RNA-seq on SUV39H2-knockout and OTS186935-treated MDA-MB-231 cells, and results showed highly consistent transcriptional changes, with 1,191 overlapping DEGs (Fig. S6A, Table S2). Expression trends of the top 50 altered genes were also largely concordant (Fig. S6B), supporting OTS186935 as an SUV39H2 inhibitor [49, 50]. Furthermore, treating MDA-MB-231 and BT-549 cells with OTS186935 markedly reduced NCOA4 methylation and ubiquitination (Fig. 7A–C and Fig. S6C–E). Importantly, OTS186935 significantly inhibited TNBC cell viability, but this inhibition was partially rescued by co-treatment with DFO (iron chelator), Fer-1 (ferroptosis inhibitor), or NAC (antioxidant) (Fig. 7D and Fig. S6F). Consistently, OTS186935 induced hallmarks of ferroptosis (Fig. 7E–H and Fig. S6G–J), which was driven by enhanced ferritinophagy (Fig. 7I–L and Fig. S7A–G). Thus, OTS186935 enhances ferritinophagy and triggers ferroptosis.

Functionally, OTS186935 treatment impaired colony formation (Fig. 7M and S7H–J), reduced cell proliferation (Fig. 7N and Fig. S7K, L), suppressed migration and invasion (Fig. S8A–G). and enhanced the DDP sensitivity (Fig. 7O and Fig. S8H). Furthermore, OTS186935 significantly suppressed MDA-MB-231 xenograft tumor growth, as evidenced by reduced tumor volume, weight, and Ki67 staining (Fig. 7P–T). OTS186935-treated tumors also showed increased iron accumulation and reduced FTH1 expression, consistent with ferroptosis activation (Fig. 7T). Notably, combining OTS186935 with DDP achieved greater tumor suppression than either treatment alone (Fig. 7Q–T). Besides, the OTS186935 and DDP-treated groups showed no evident organ damage or abnormalities were observed, exhibiting no significant toxicity (Fig. S8I, J). To further investigate the clinical translational potential of OTS186935, we conducted pharmacokinetic investigations to assess the absorption and safety profile. Pharmacokinetic profiling indicated effective oral absorption, characterized by a C_max_ of 42.0 ng/mL, a T_max_ of 0.389 h, and an AUC_0-t_ of 275 hr*ng /mL, corresponding to an absolute oral bioavailability (F) of 17.1% (Table S3). The plasma disappearance half-time was 10.3 h, and at 24 h post-injection, the injected OTS186935 had almost completely disappeared from the blood (Table S3). Together, these results support the favorable oral absorption and safety of OTS186935 in mice.

## Discussion

Iron is essential for normal cell survival, functioning as a cofactor in numerous processes, including oxygen transport, oxidative phosphorylation, and enzymatic reactions essential for cell proliferation [51]. In cancer, this regulation is often disrupted, resulting in an enhanced dependence on iron, that supports tumor initiation and growth [52]. Reprogramming iron metabolism to disrupt this iron homeostasis and release excess iron can trigger ferroptosis, presenting a therapeutic vulnerability in certain cancers. To date, the mechanism for maintaining iron levels is still unclear. In this study, we identified a NCOA4^low^-FTH1^high^ ferritinophagy axis that sustains iron homeostasis in TNBC. This phenomenon is consistent with the previously reported functions of NCOA4-FTH1 in ferroptosis and ferritinophagy [14]. NCOA4 overexpression reactivated ferritinophagy, promoted iron release from ferritin, induced ferroptosis, and suppressed tumor cell proliferation. Mechanistically, SUV39H2 methylated NCOA4 at K356, enhancing its interaction with the E3 ubiquitin ligase HERC2 and leading to NCOA4 ubiquitination and proteasomal degradation. Consequently, ferritinophagy and ferroptosis were suppressed, promoting TNBC progression and chemoresistance.

In addition to the methylation, HIF also serves as a major transcriptional regulator of cellular iron levels [53]. Li et al. further uncovered the molecular mechanisms in which dual HIF1α and HIF2α can directly transcriptionally activate NCOA4 in response to iron demand in hepatocytes [54]. The regulation of NCOA4 involves a multifaceted interplay between the SUV39H2-mediated epigenetic axis and the HIF-driven transcriptional axis. The SUV39H2-mediated epigenetic axis may function as an independent regulatory module that either fine-tunes or counterbalances HIF-mediated NCOA4 activation. This regulatory interplay likely endows TNBC cells with metabolic flexibility. Under normal conditions, it maintains ferroptosis resistance, while allowing rapid iron mobilization to support proliferation when needed.

Fe-S clusters are essential cofactors for numerous enzymes involved in critical cellular processes, including mitochondrial electron transport, DNA repair, and metabolic catalysis[55]. In rapidly proliferating tumor cells, the demand for Fe-S clusters is markedly elevated [18] and the canonical pathway for releasing stored iron to meet this demand is NCOA4-mediated ferritinophagy[56]. Our findings that SUV39H2 downregulates NCOA4 protein levels establishes a novel layer of NCOA4 regulation. By dampening ferritinophagy, SUV39H2 may act as a brake on iron release, potentially creating a controlled, steady supply of iron for Fe-S cluster assembly rather than a surge that could lead to iron toxicity and ferroptosis. This restraint could be particularly important in TNBC, where high metabolic activity must be balanced against oxidative stress.

Lysosomes possess a large amount of iron and play a crucial role in iron homeostasis by integrating signals from different subcellular compartments and cell death pathways [57]. Lysosomal iron overload causes lipid peroxide production, leading to ferroptosis in cancer. Consistently, we found NCOA4 overexpression or the inhibition of NCOA4 methylation can reactivate ferritinophagy, promote the fusion of autophagosomes and lysosomes for iron release from ferritin, and induced ferroptosis. Subsequently, for this property, a small-molecule activator of lysosomal iron—fentomycin-1-were designed to induce the oxidative degradation of phospholipids and ultimately ferroptosis in cancer [58]. Similarly, we discovered the SUV39H2 inhibitor OTS186935 increased intracellular iron, reduced FTH1 levels, enhanced LC3-II expression, and promoted ferritin–lysosome colocalization, collectively indicating robust induction of ferritinophagy and ferroptosis (Fig. 7). These data indicate that pharmacologic activation of lysosomal iron may server a promising therapeutic strategy in cancer.

Several limitations should be noted. First, definitive validation of NCOA4 K356 monomethylation awaits the development of a site-specific antibody, as current evidence is indirect. Second, given the high sequence homology between SUV39H2 and related methyltransferases (SUV39H1, EZH2, G9a, SETDB1), it would be important to assess the biochemical selectivity of OTS186935 against these enzymes. However, due to technical and resource constraints, such experiments could not be performed in the current study. Therefore, we have reclassified OTS186935 as an SUV39H2 inhibitor rather than a selective one.

In summary, we identify that TNBC cells exhibit “iron addiction” through the NCOA4^low^-FTH1^high^ ferritinophagy axis. NCOA4 K356 methylation promotes NCOA4 degradation and suppresses ferritinophagy; whereas K356R stabilizes NCOA4, reactivates ferritinophagy, and induces ferroptosis. Thus, targeting NCOA4 methylation with OTS186935 represents a promising therapeutic strategy to enhance ferroptosis vulnerability and chemotherapy efficacy in TNBC.

## Materials And Methods

### Cell culture

All cell lines (MDA-MB-468, MDA-MB-453, MDA-MB-231, BT-549, MCF10A, HEK293T) were obtained from the American Type Culture Collection (ATCC, Manassas, VA, USA). All cell lines were cultured at 37 °C in an incubator with 5% CO_2_ atmosphere. The medium used for MDA-MB-468, MDA-MB-453, MDA-MB-231, BT-549, and HEK293T cells was supplemented with DMEM, 10% fetal bovine serum, and 1% penicillin/streptomycin. MCF10A cells were cultured in complete MCF10A culture medium. Prior to the experiment, these cells were screened for mycoplasma contamination, interspecies cross-contamination, and authenticity.

### Data source

RNA-seq data and corresponding clinical information related to TNBC were acquired from TCGA data portal (https://portal.gdc.cancer.gov/) to identify differentially expressed genes (DEGs) in TNBC tissues, compared with normal breast tissues. Furthermore, the differentially expressed genes of TNBC were identified using the “edgeR” package of R software.

### Antibodies and reagents

A description of antibodies and reagents was detailed in Supplementary Materials and Methods.

### Plasmids and viral infection

Detailed descriptions of lentiviral production and infection were presented before [59].

Experiments were carried out as described in Supplementary Materials and Methods.

### GST pulldown assay and in vitro methylation assay

GST or GST-NCOA4 and His-SUV39H2 fragmental proteins were expressed in bacteria (BL21) induced with IPTG and then purified, as described previously [59, 60]. The detailed protocols of GST pulldown assay and in vitro methylation assay were shown in Supplementary Materials and Methods.

### Mass spectrometry analysis

The details of NCOA4 sample preparation for methylation detection by MS analysis were shown in Supplementary Materials and Methods. LC-MS/MS was performed in the Academy of Biological Sciences Proteomics Research and Analysis Center, Shanghai.

### Immunoprecipitation

The details of immunoprecipitation were shown in Supplementary Materials and Methods.

### Immunofluorescence

The protocol of immunofluorescence was shown in Supplementary Materials and Methods.

### Molecular docking

A description of molecular docking was detailed in Supplementary Materials and Methods.

### System sets and Molecular dynamics simulations

Detailed descriptions of the system sets were provided in the Supplementary Materials and Methods.

### Gene expression correlation analysis, Survival analysis and Prediction of IC50 for chemotherapy drugs

A description was detailed in Supplementary Materials and Methods.

### RNA sequencing

A description of RNA sequencing was detailed in Supplementary Materials and Methods.

### ICP-OES analysis

One gram of homogenized tissue digested in a microwave oven with 10 mL of nitric. An inductively coupled plasma optical emission spectrophotometer (ICP OES-Optima 3000DV Perkin-Elmer-Norwalk, USA) was used for the analysis of digested samples. Fe was quantified at 259.940 nm, and all values were normalized to dry weight.

### Iron content determination

The protocol of iron assay was shown in Supplementary Materials and Methods.

### Measurement of Fe^2+^ levels

Cells were seeded in 24-well plates and incubated with 10 μM PGSK in serum-free DMEM. After staining, cells were washed three times with serum-free DMEM to remove unbound dye. The levels of Fe^2+^ were then quantified using flow cytometry and visualized via confocal microscopy.

### Measurement of ROS and lipid ROS

The detailed procedure was shown in Supplementary Materials and Methods.

### MMP measurement

Cells were seeded in 24-well plates and incubated with 2 μM Rho123, which was diluted 1:250 in serum-free DMEM. After staining, cells were washed three times with serum-free DMEM to remove unbound dye. MMP was then analyzed using confocal microscopy.

### Transmission electron microscopy

The protocols were shown in Supplementary Materials and Methods.

### Cell viability assay

Cell viability was measured using the CCK-8 kit and the assay was detailed in Supplementary Materials and Methods.

### Clone formation assay

Experiments were carried out as described in Supplementary Materials and Methods.

### Cell count

Cells were seeded at 1000 per well in 6-well plate and incubated overnight at 37 °C. After managements, cells were counted every 24 h for 5 days.

### EdU assay

The detailed method of EdU assay was described in the Supplementary Materials and Methods.

### Wound-healing, migration and invasion assays

Experiments were carried out as described in the Supplementary Materials and Methods.

### Adhesion experiments

Methods were detailed in the Supplementary Materials and Methods.

### Animal experiments

The animal assay was performed the same protocol as described previously [59]. And the detailed protocols were shown in Supplementary Materials and Methods.

### Immunohistochemistry, Hematoxylin–Eosin (HE) staining and Prussian blue staining

The detailed protocols were shown in Supplementary Materials and Methods.

### ALT, CRE, AST, BUN testing and Pharmacokinetic study

The detailed protocols were shown in Supplementary Materials and Methods.

### Data analysis

All statistical analyses were performed using GraphPad Prism 8.0 (GraphPad, San Diego, CA). Data are expressed as mean ± standard deviations. The sample size (n) for each statistical analysis is shown in the legend. The survival curves were constructed by Kaplan-Meier analysis and log-rank test. Pearson correlation analysis and Spearman’s test were used. All other comparisons employed the Student’s two-tailed t-test and one-way ANOVA and Tukey’s test to determine the statistical significance of differences between groups. The statistical significance was set as *p* < 0.05, *p < 0.05, **p < 0.01, ***p < 0.001.

## Supplementary information

Supplementary Figures

Supplementary Materials and Methods

Table S1

Table S2

Table S3

Full and uncropped western blot

## Data Availability

All data presented in the Results were generated by the authors and are available upon request.
