# Supplementary material for: SUV39H2-mediated NCOA4 methylation controls ferritinophagy and ferroptosis in triple-negative breast cancer
Source: Cell Death Dis. 2026 Jul 16;17(1):876. doi: 10.1038/s41419-026-09008-1 (PMC13654947; doi:10.1038/s41419-026-09008-1)

Figure 1

K

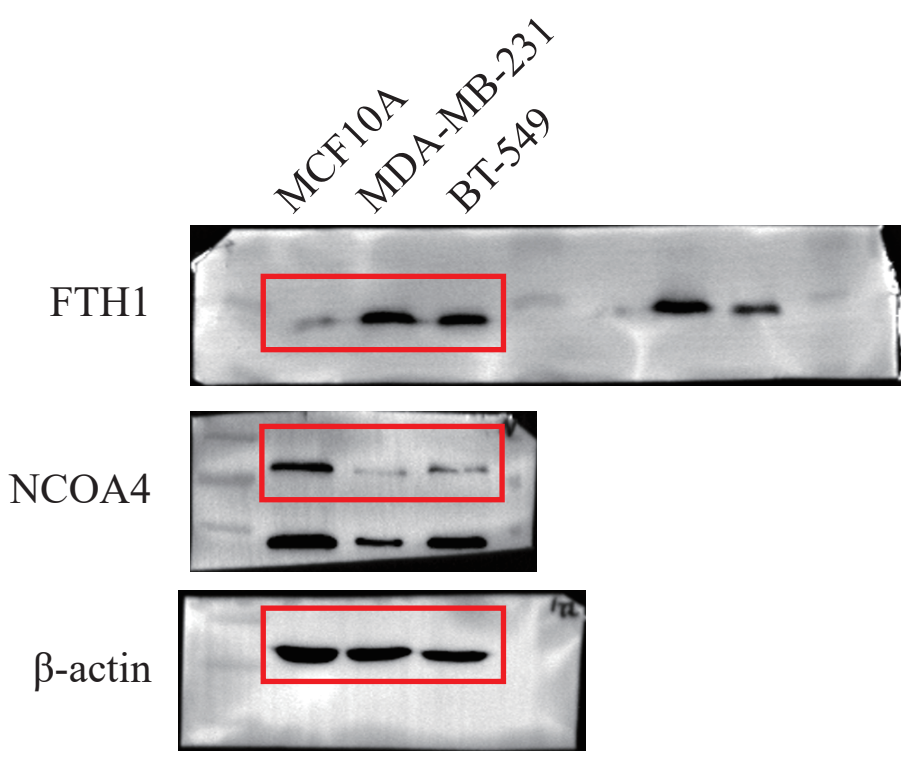

Figure 1

L

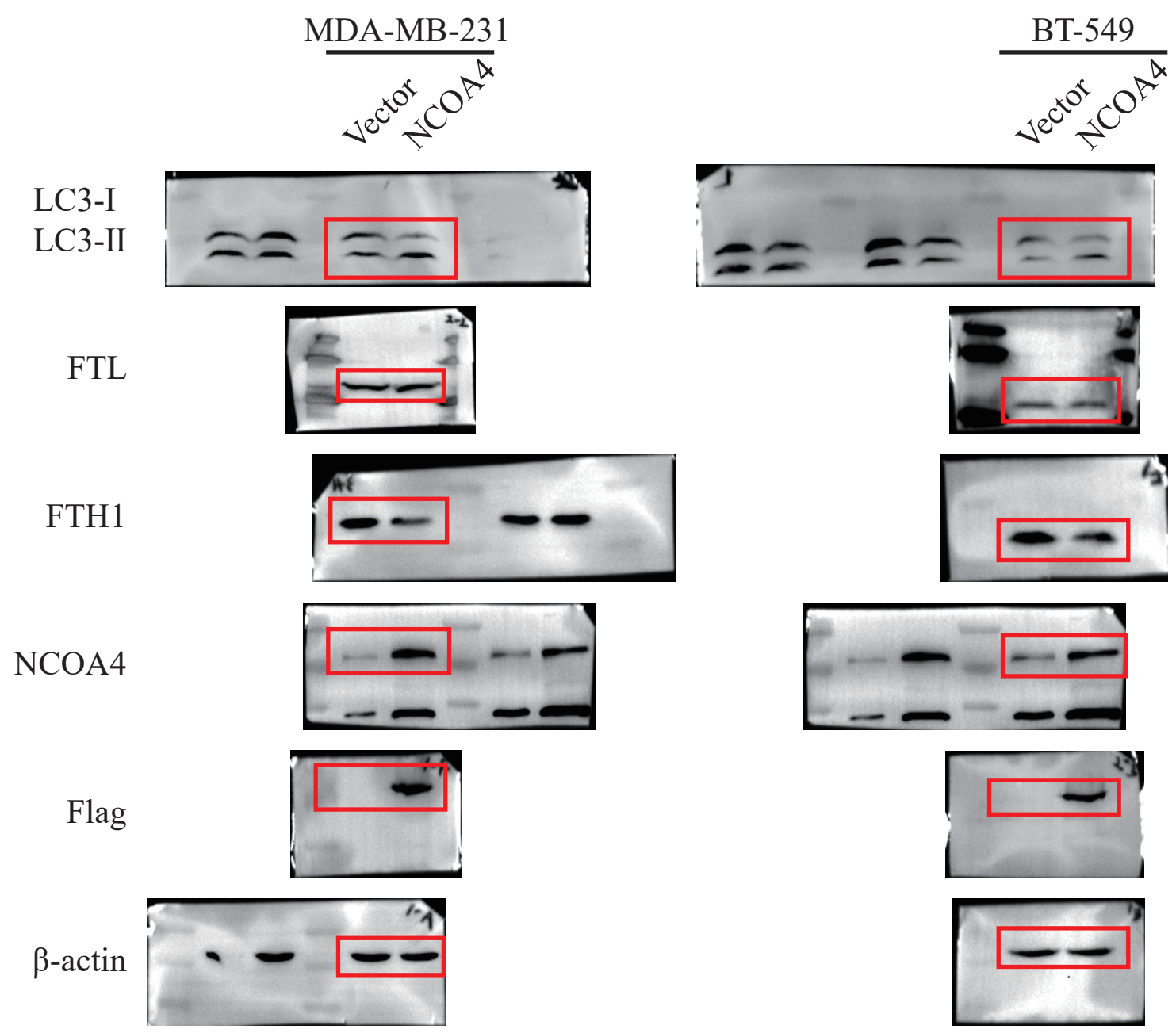

Figure 2

B

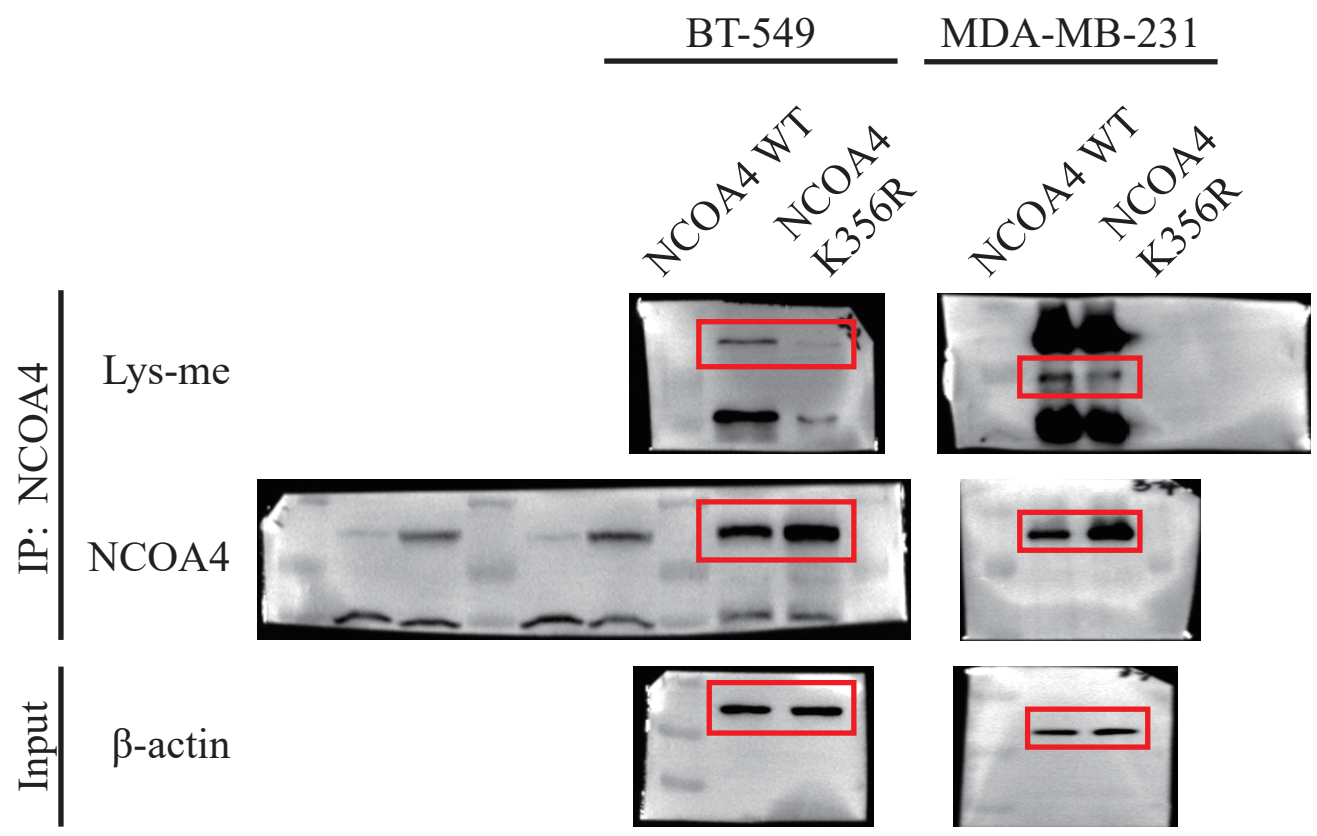

Figure 2

C

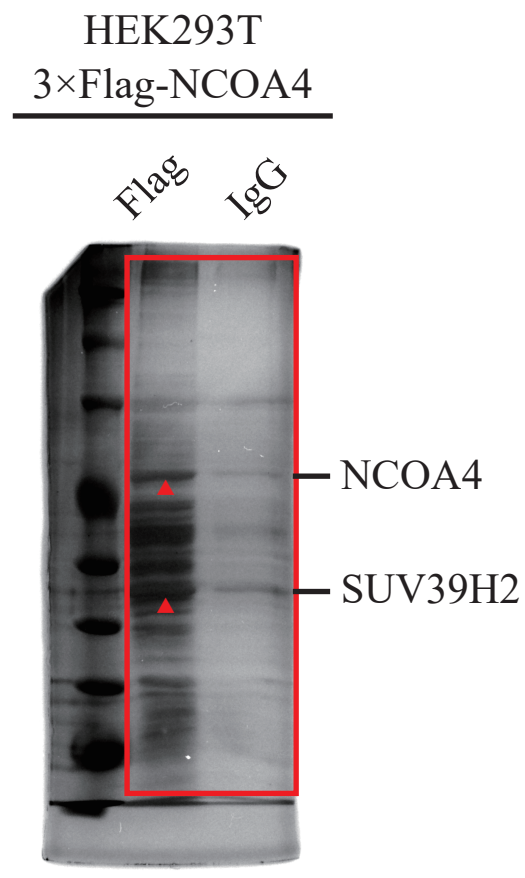

Figure 2

D

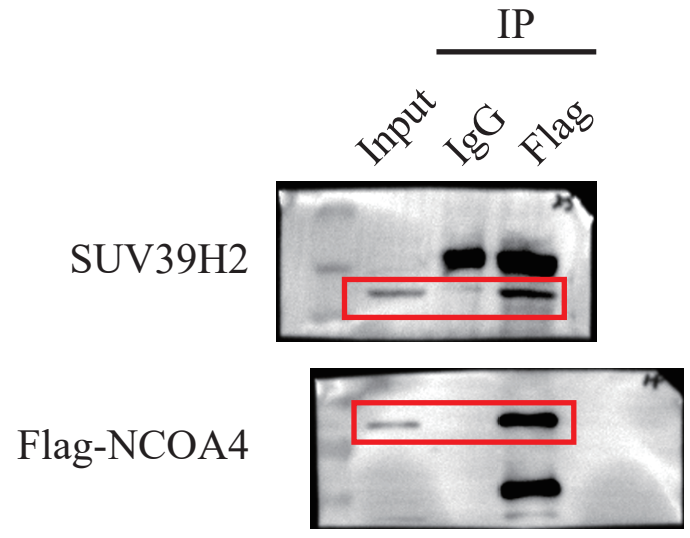

E

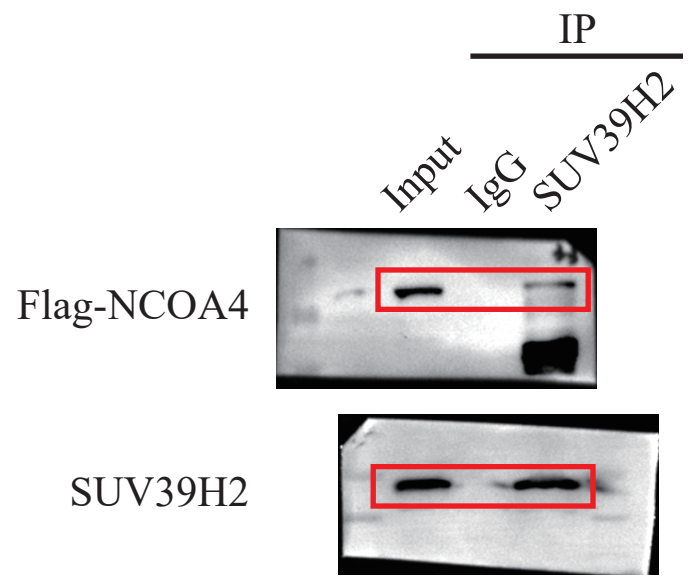

Figure 2

G

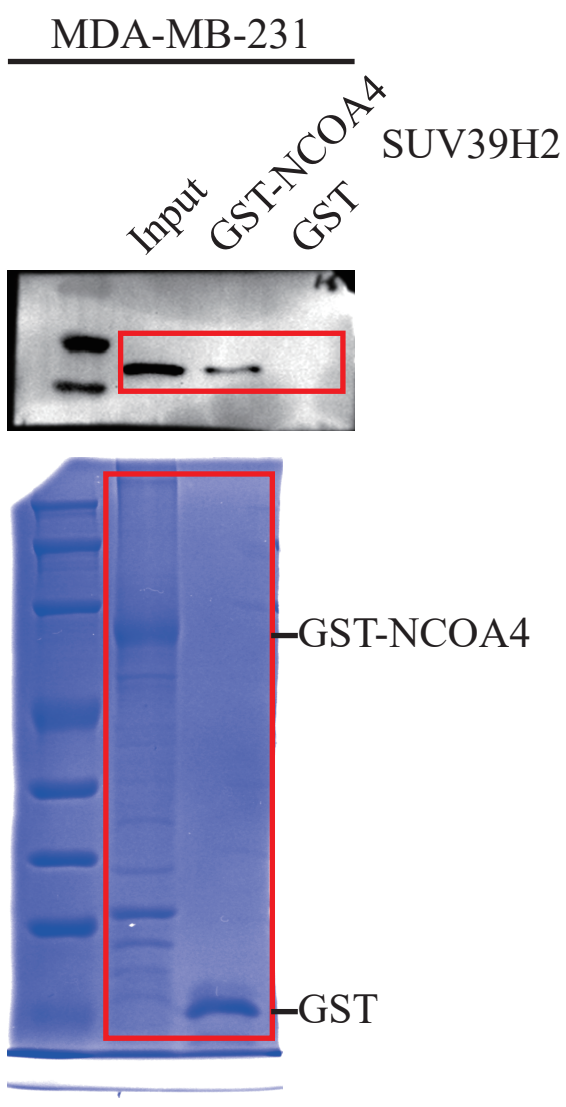

Figure 2

H

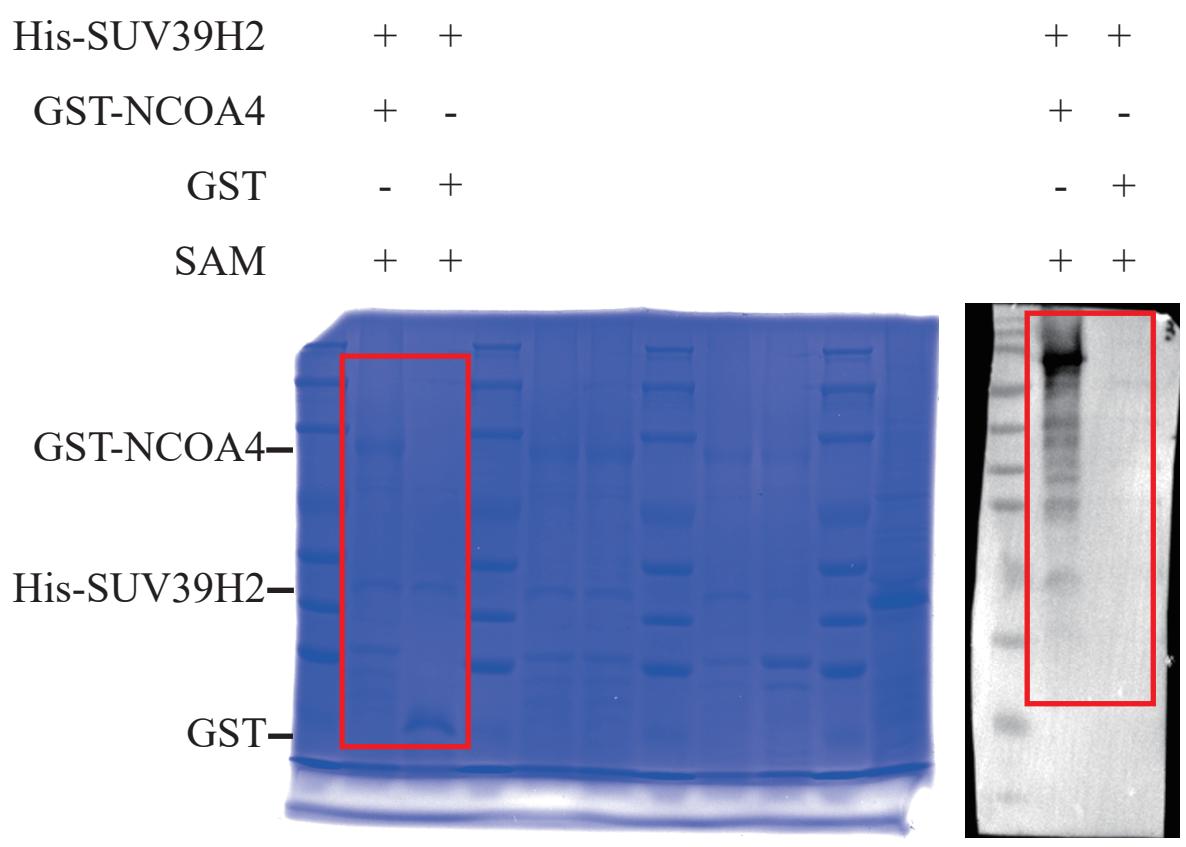

Figure 2

I

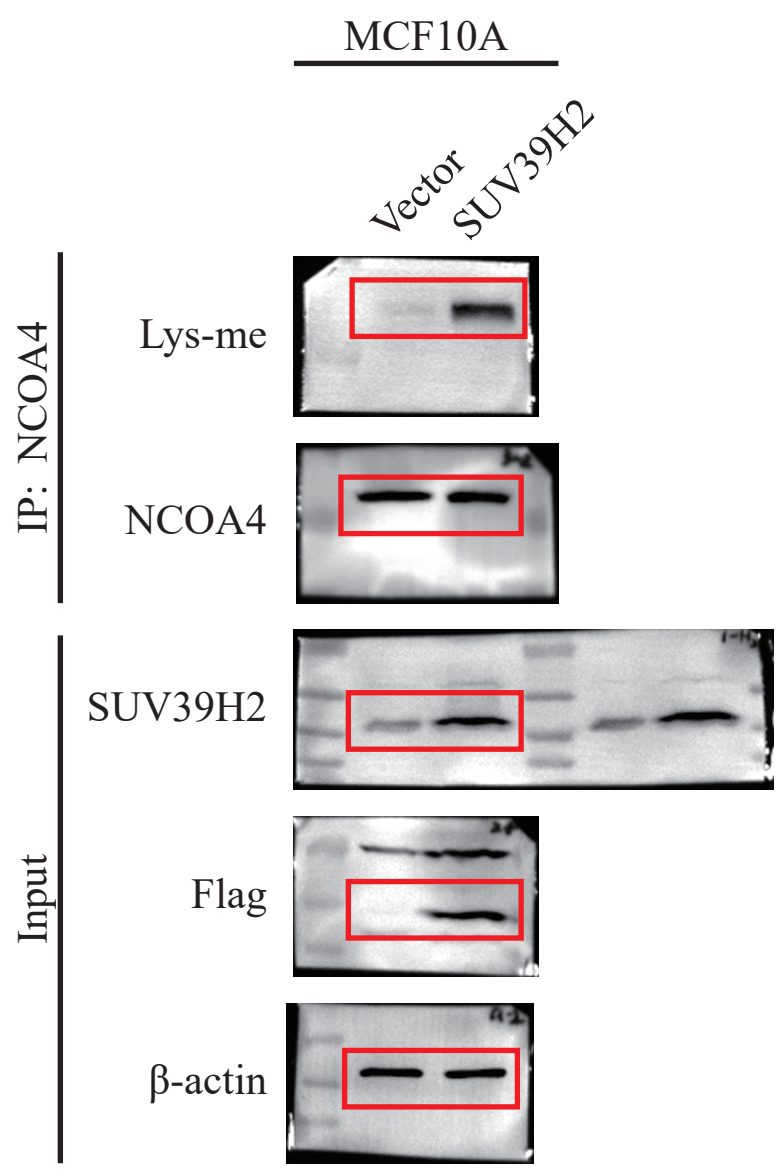

Figure 2

K

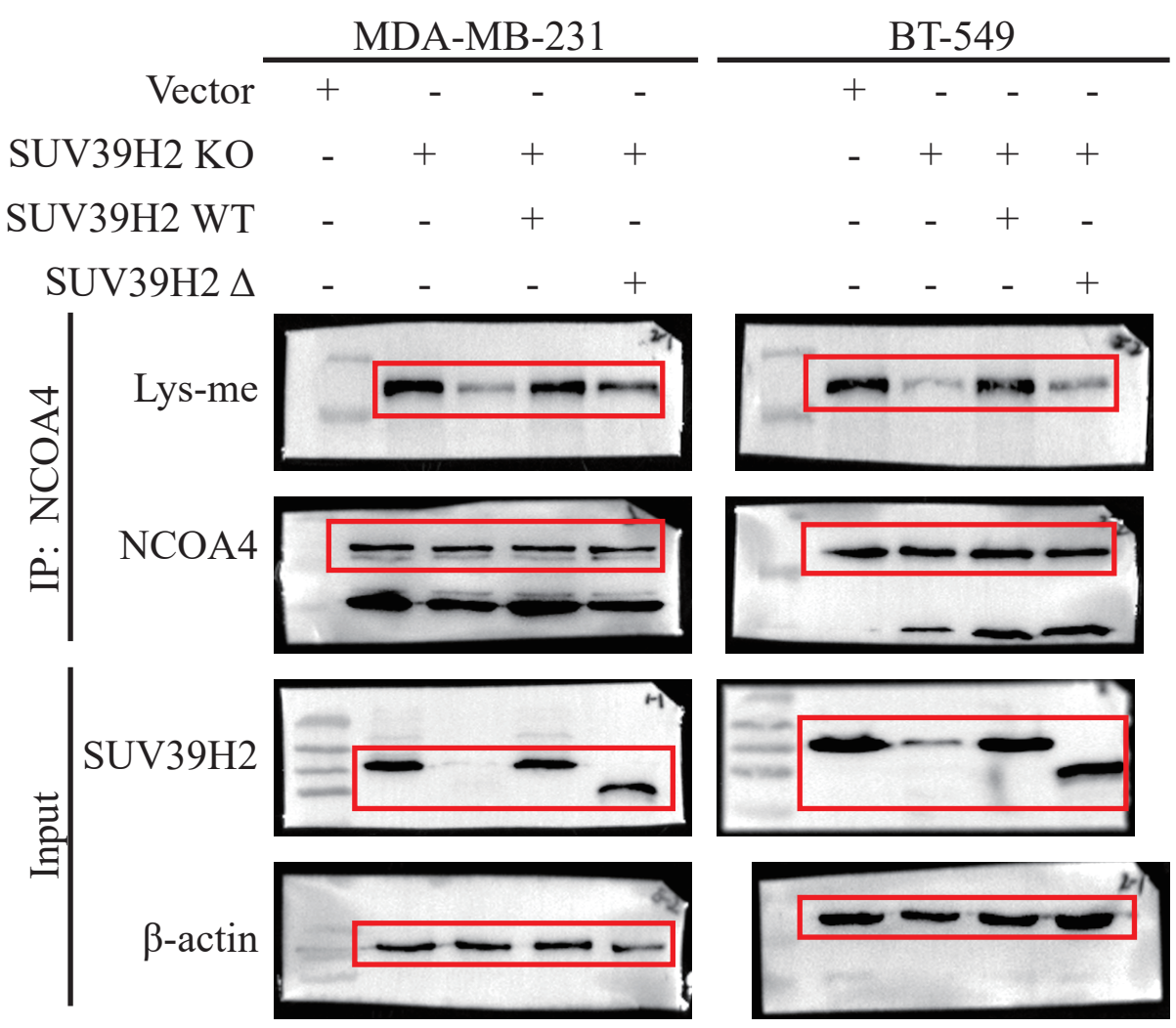

Figure 2

L

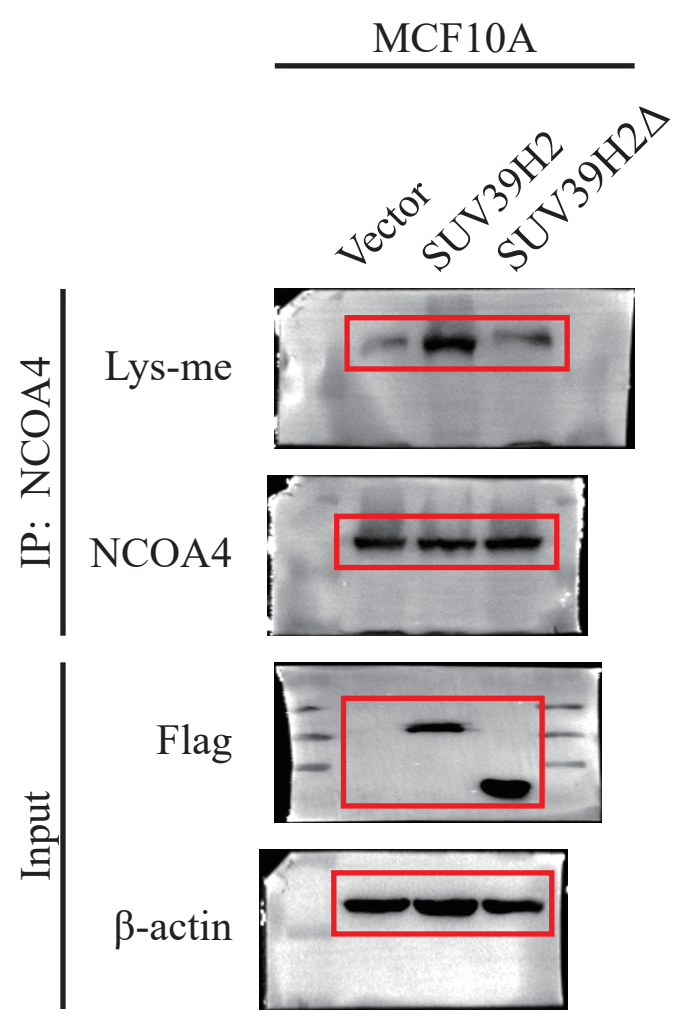

Figure 2

M

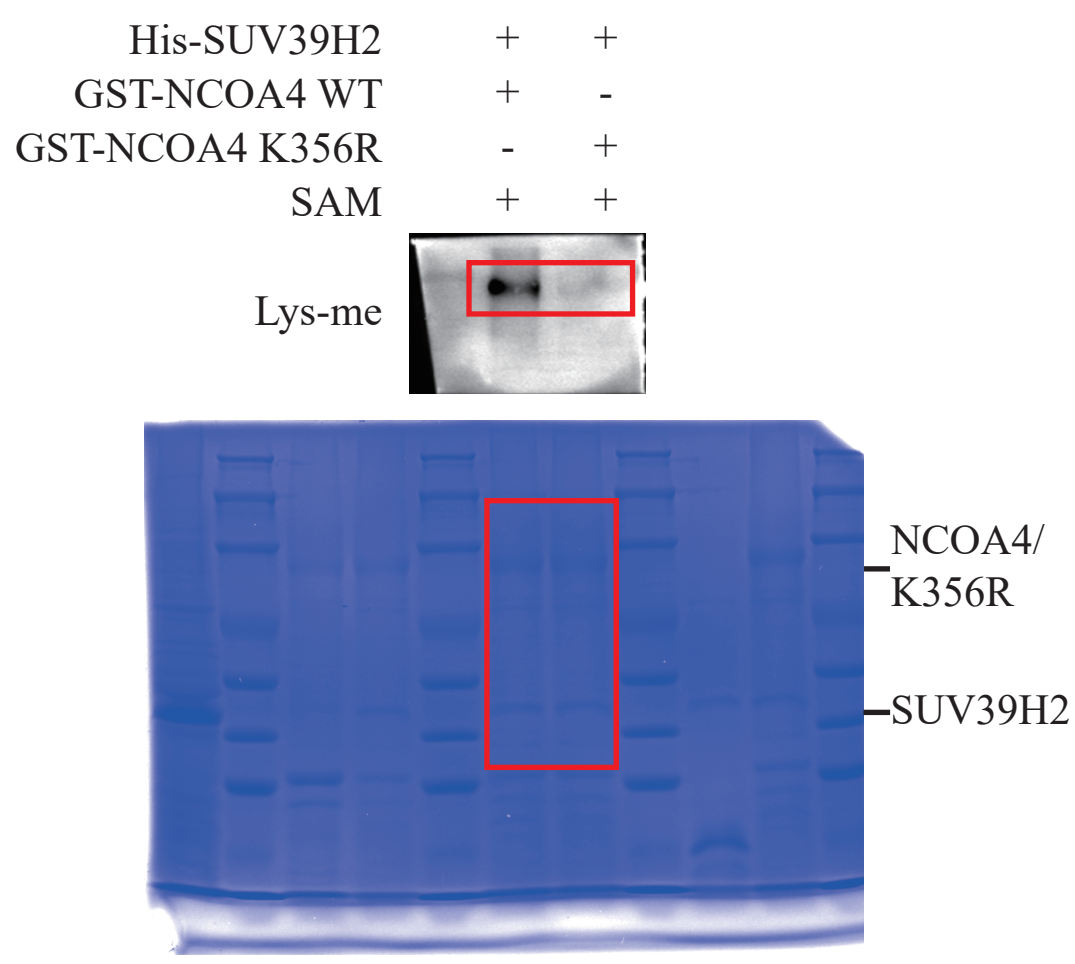

Figure 3

C

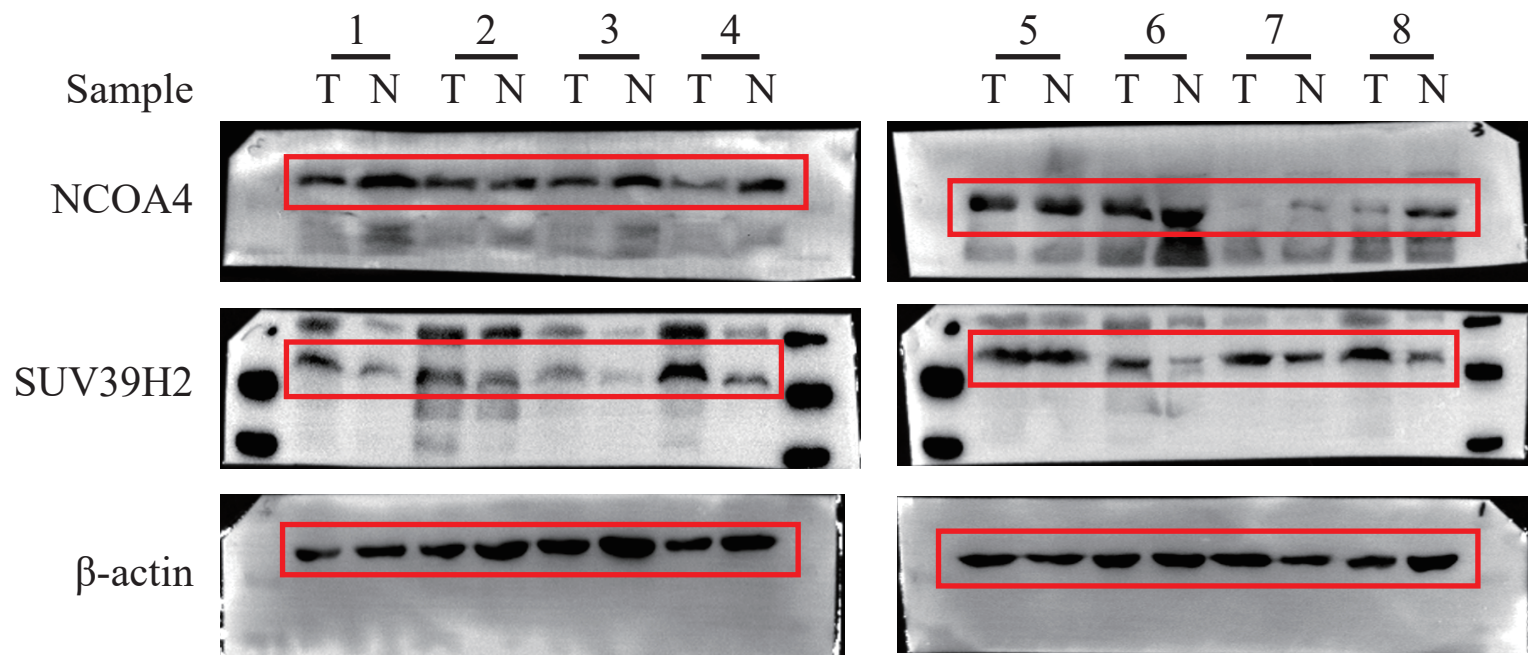

Figure 3

E

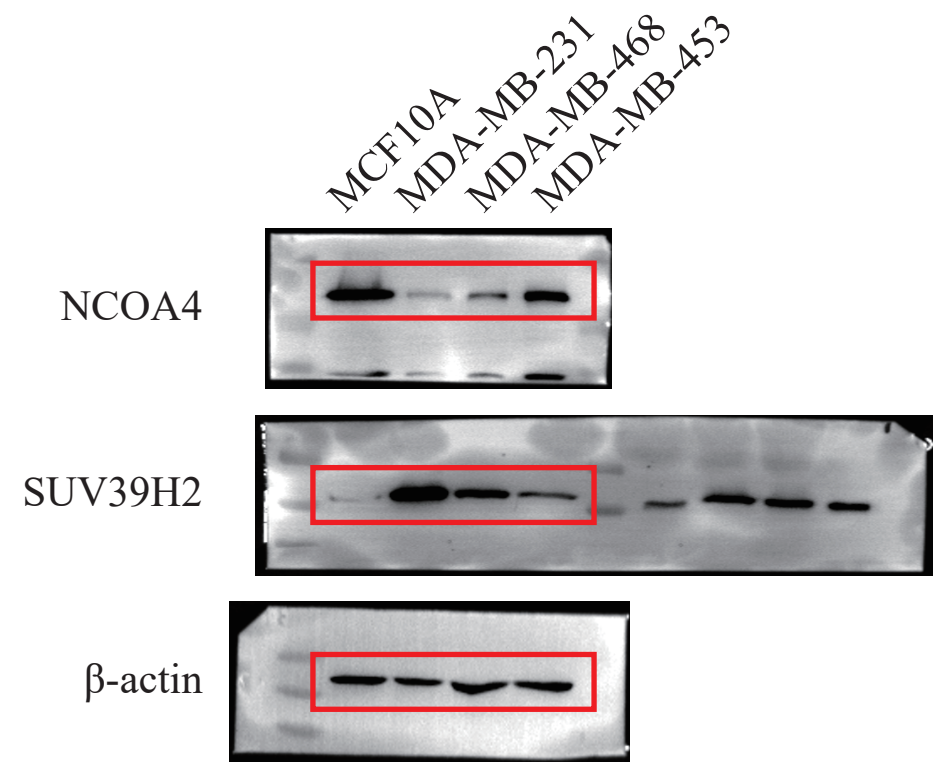

Figure 3

D

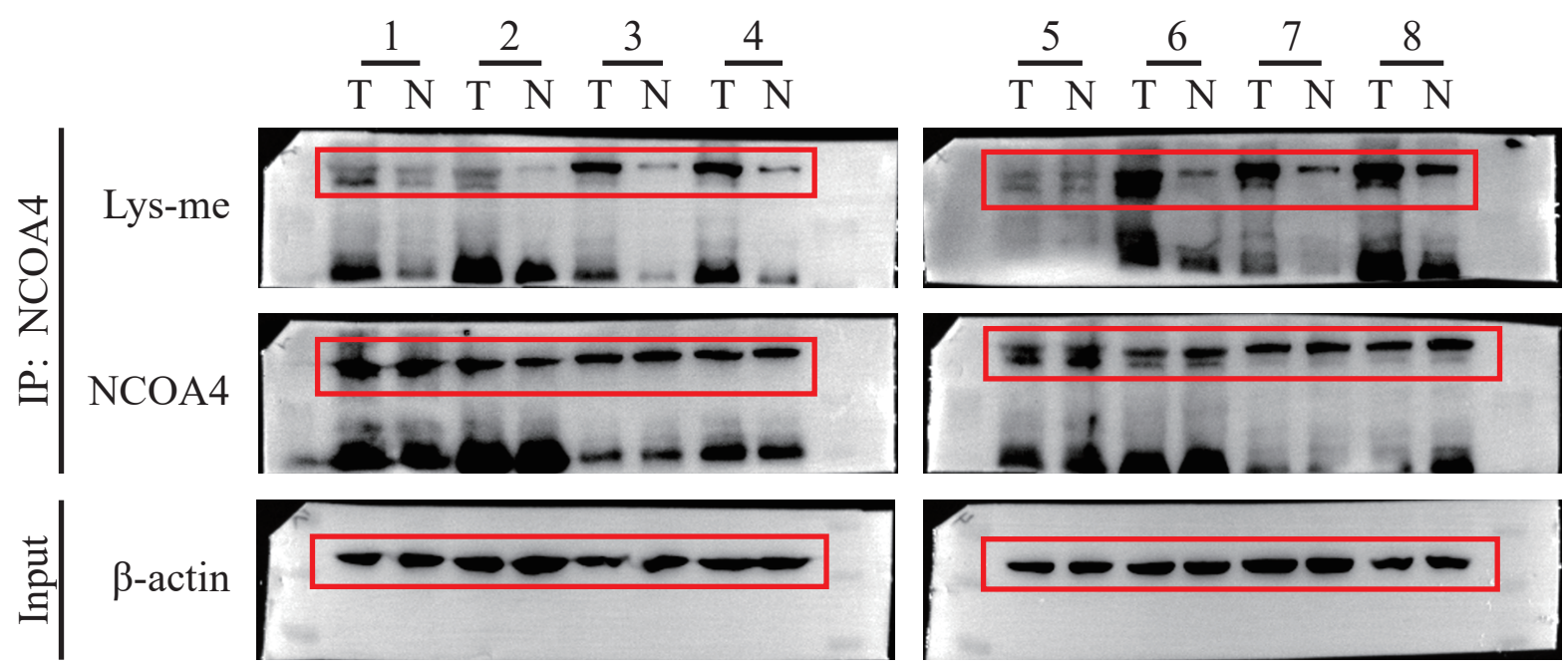

Figure 3

F

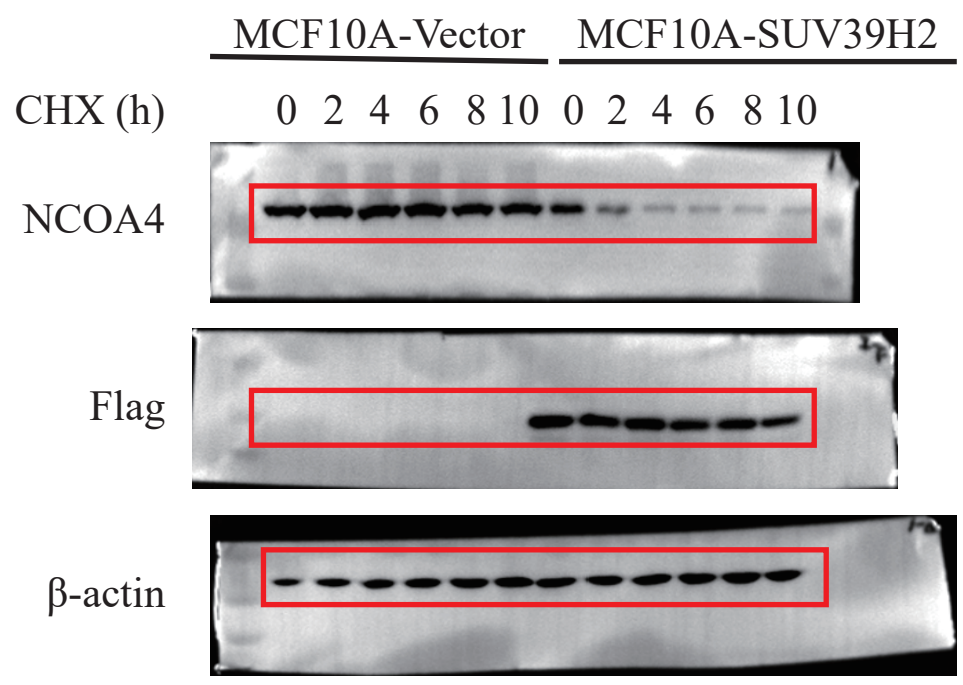

Figure 3

G

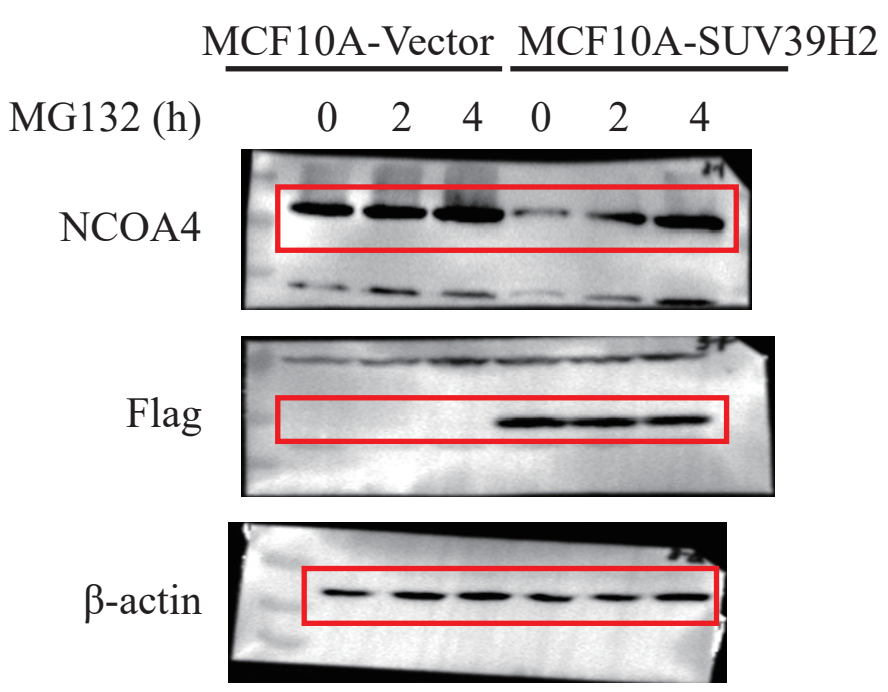

Figure 3

H

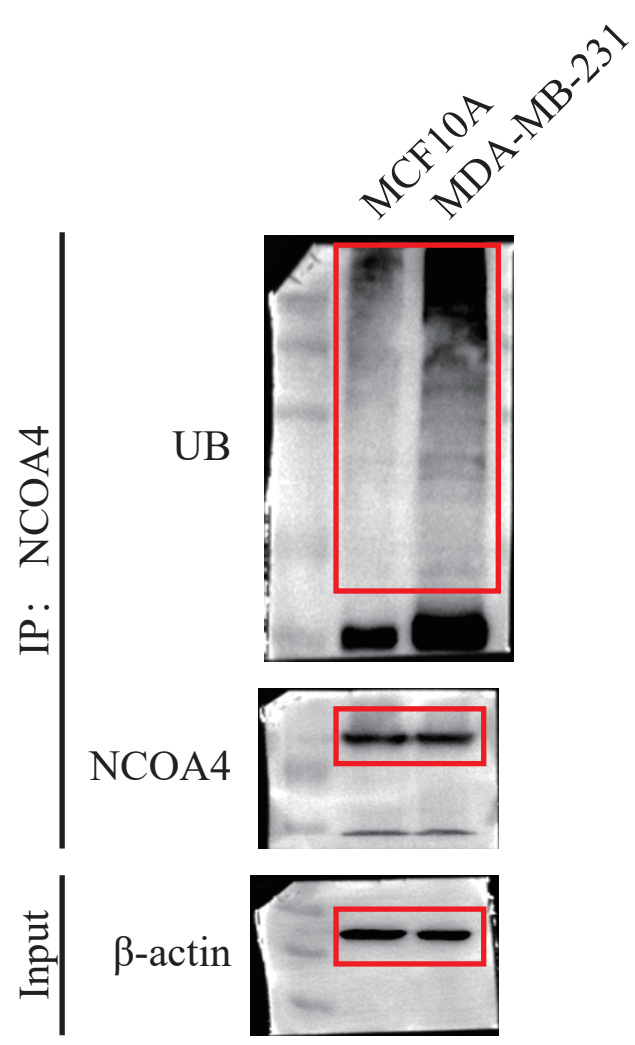

Figure 3

I

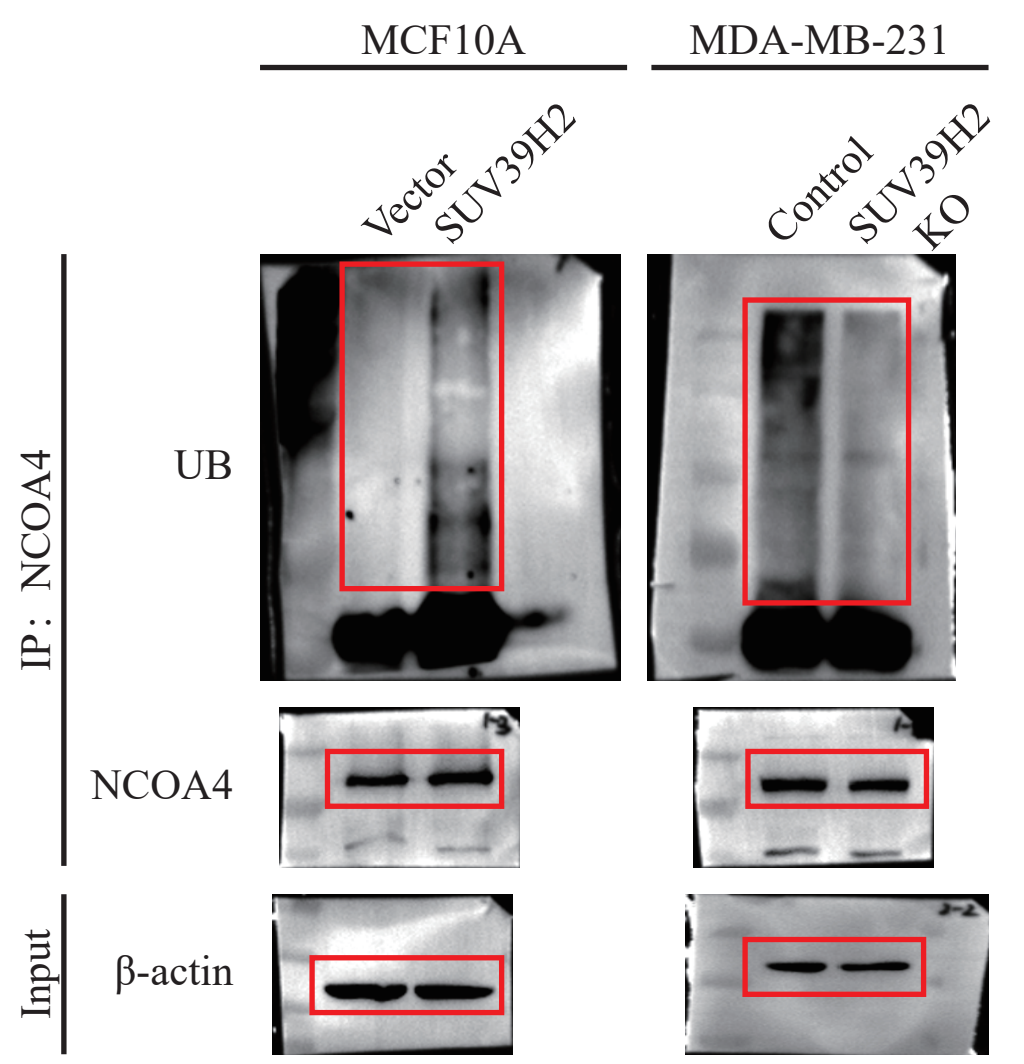

Figure 4

B

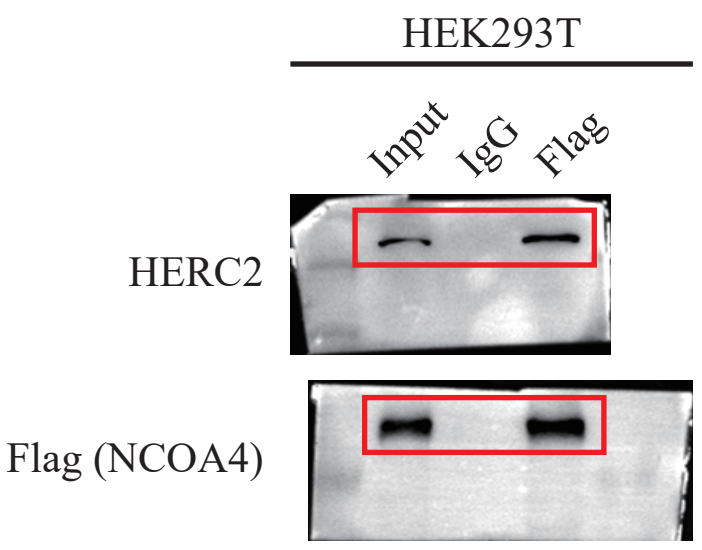

Figure 4

C

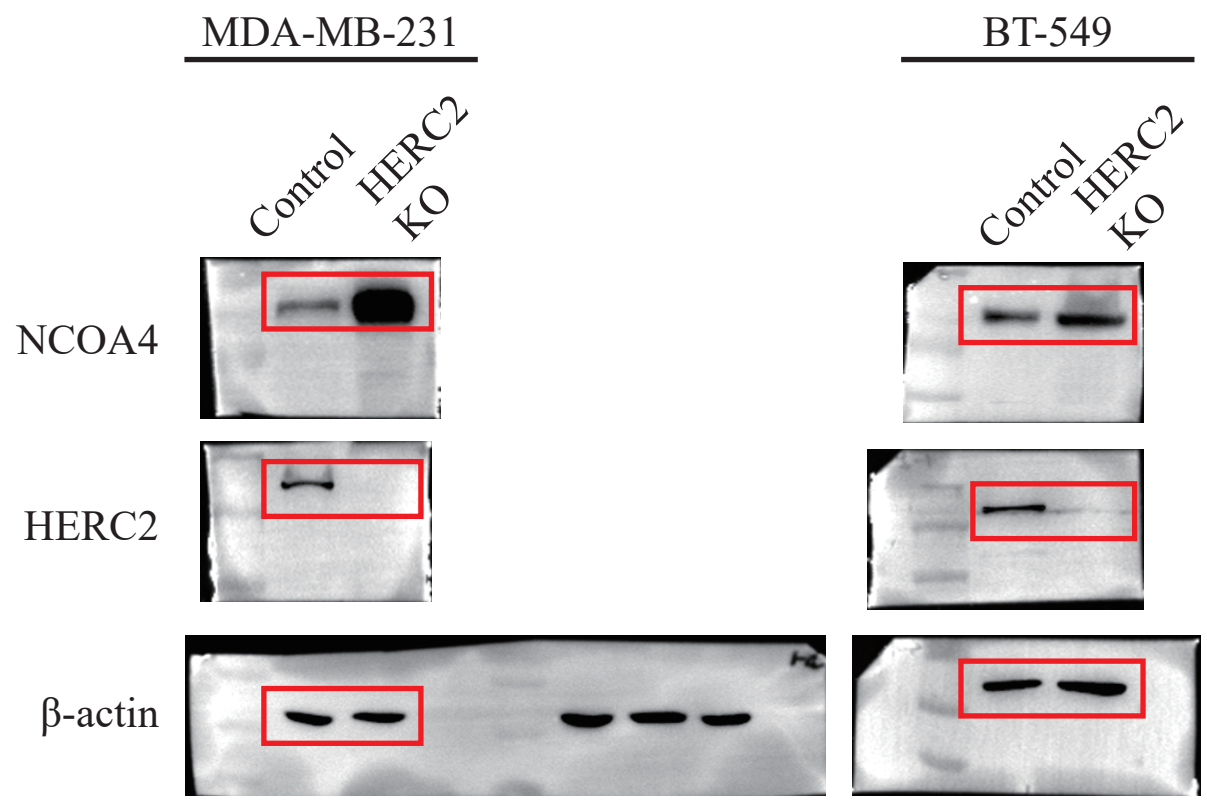

Figure 4

D

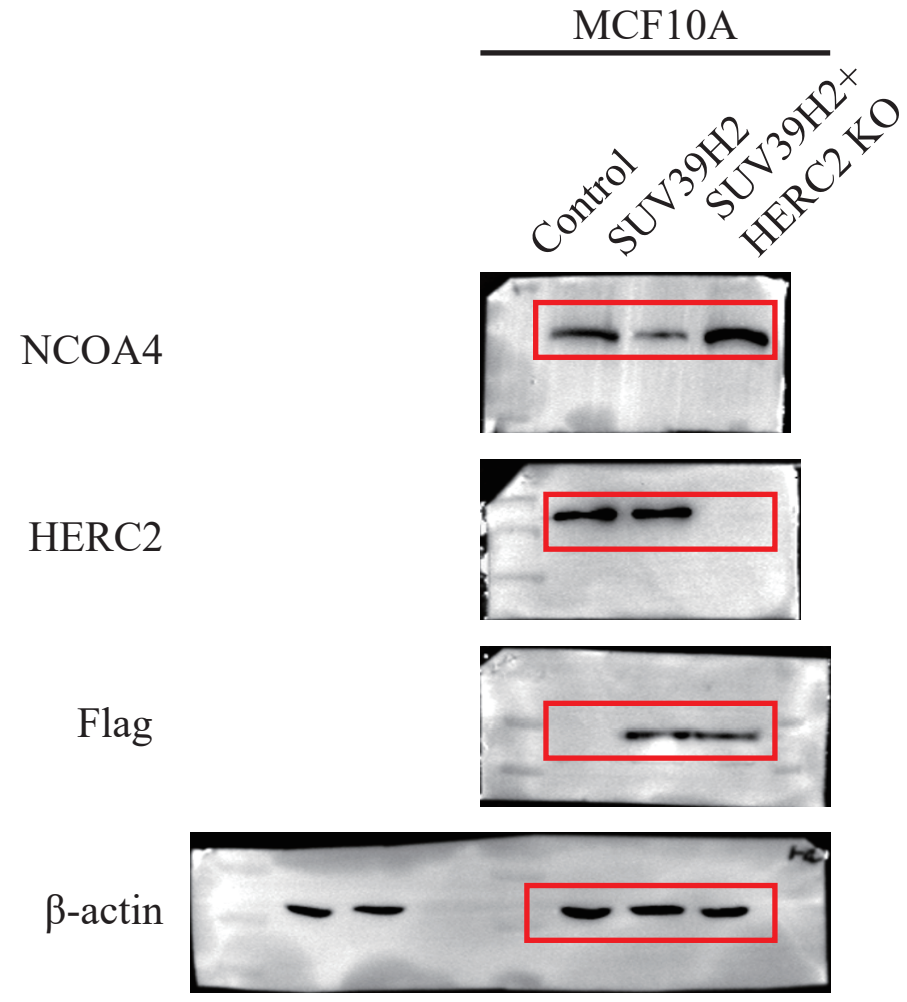

Figure 4

E

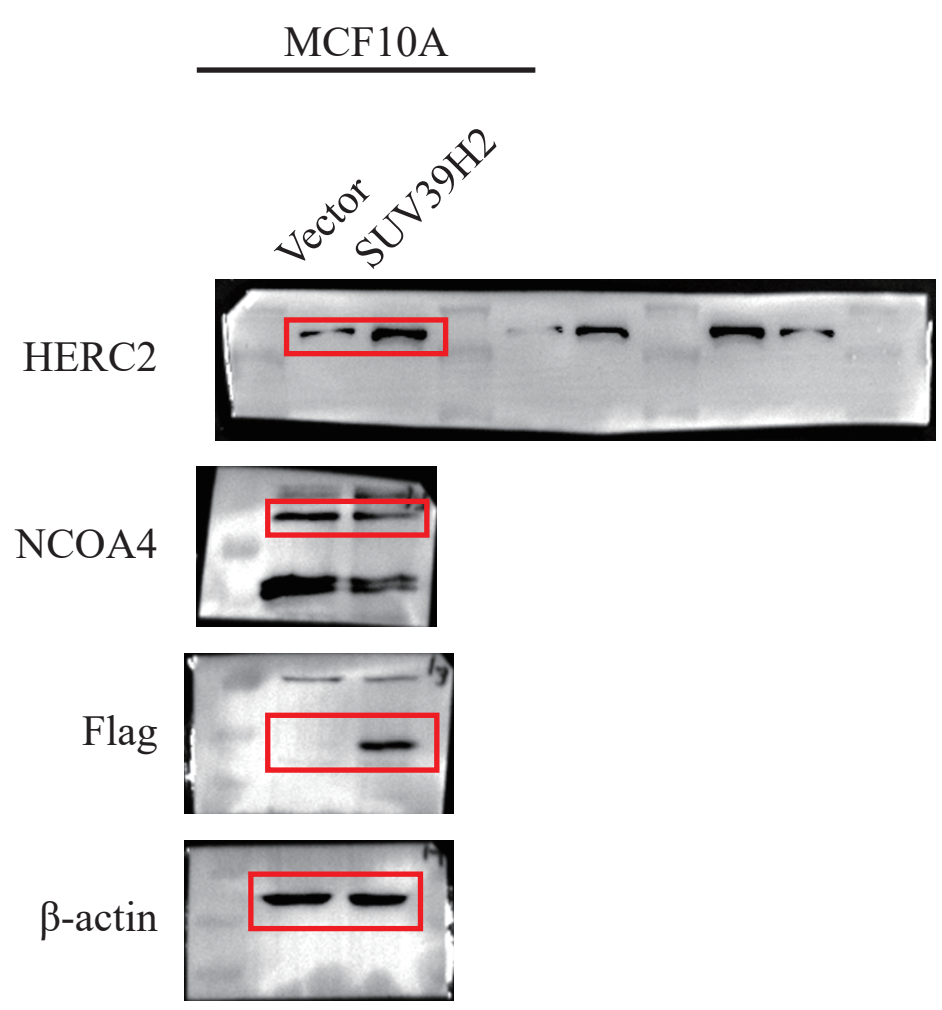

Figure 4

F

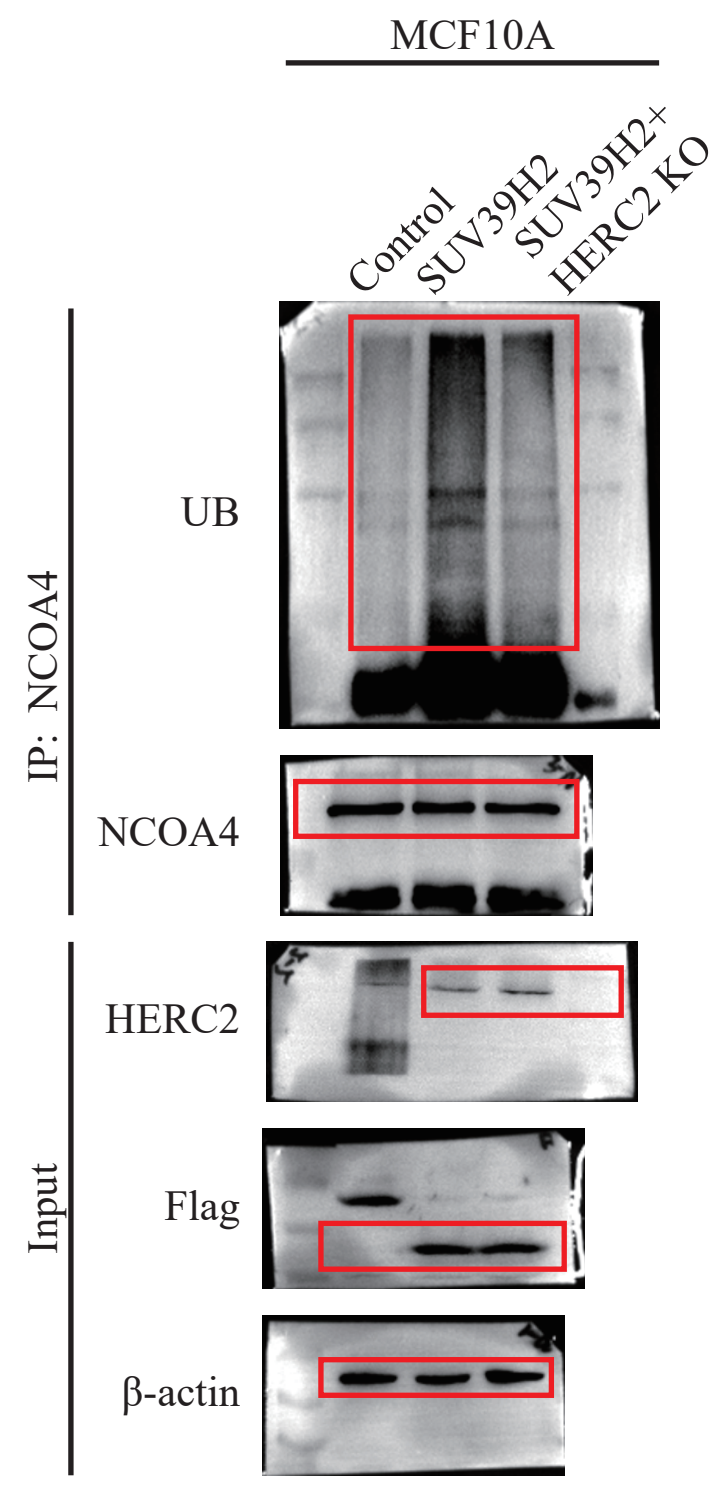

Figure 4

G

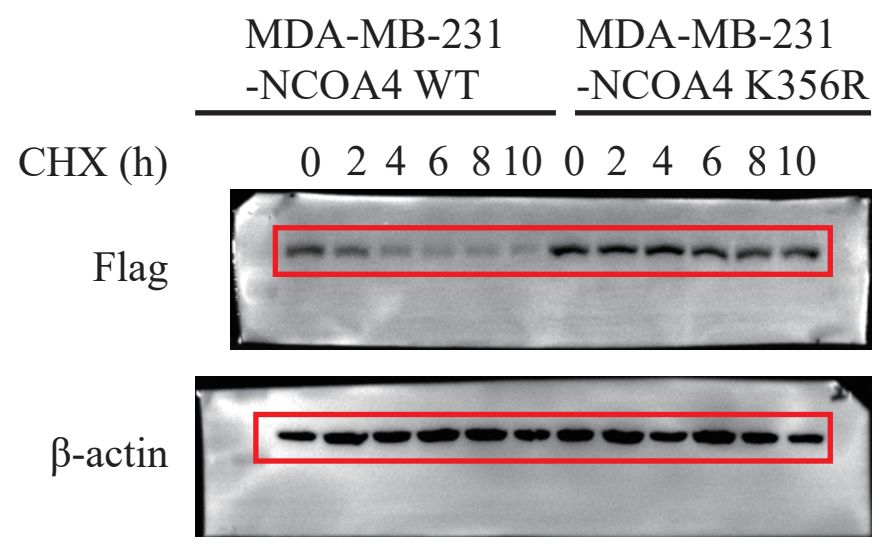

Figure 4

H

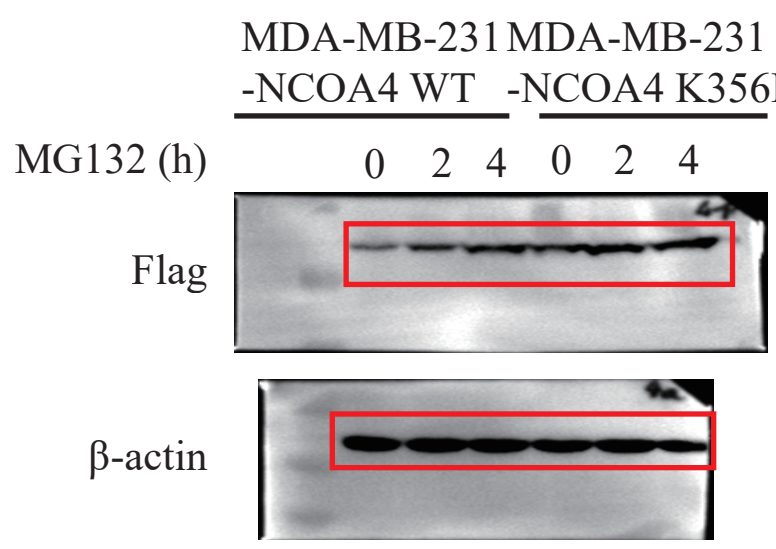

Figure 4

I

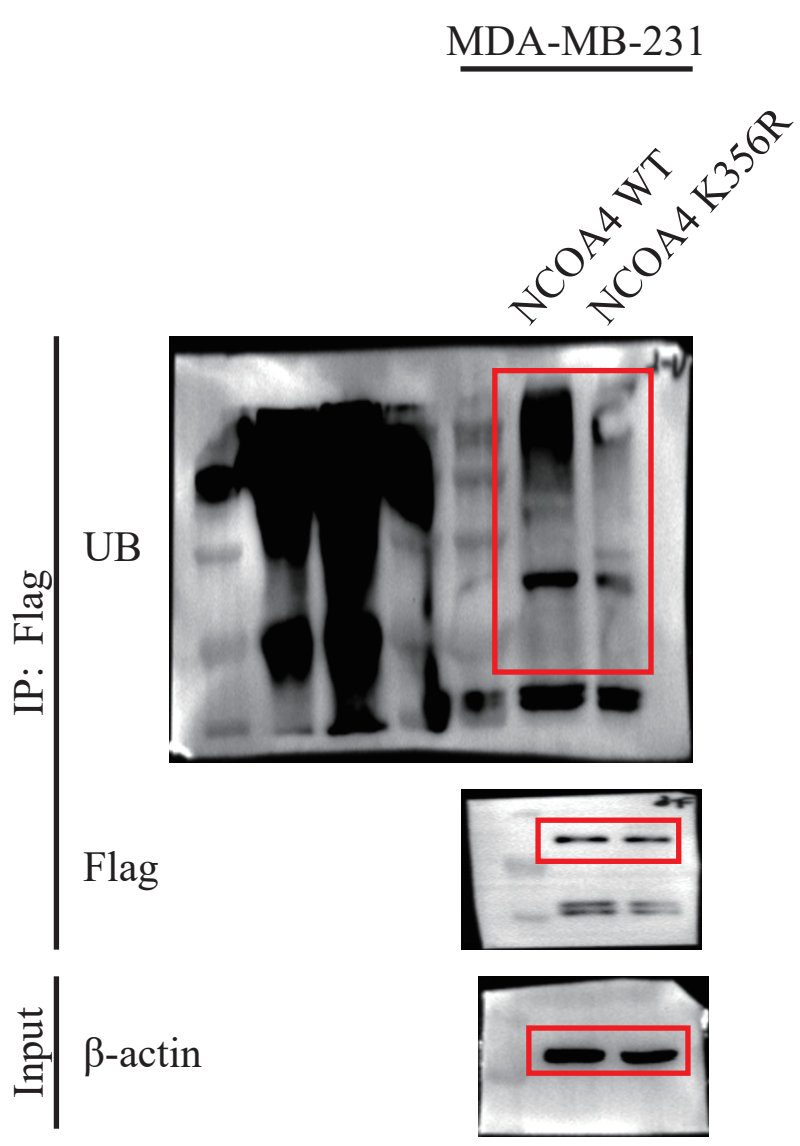

Figure 4

J

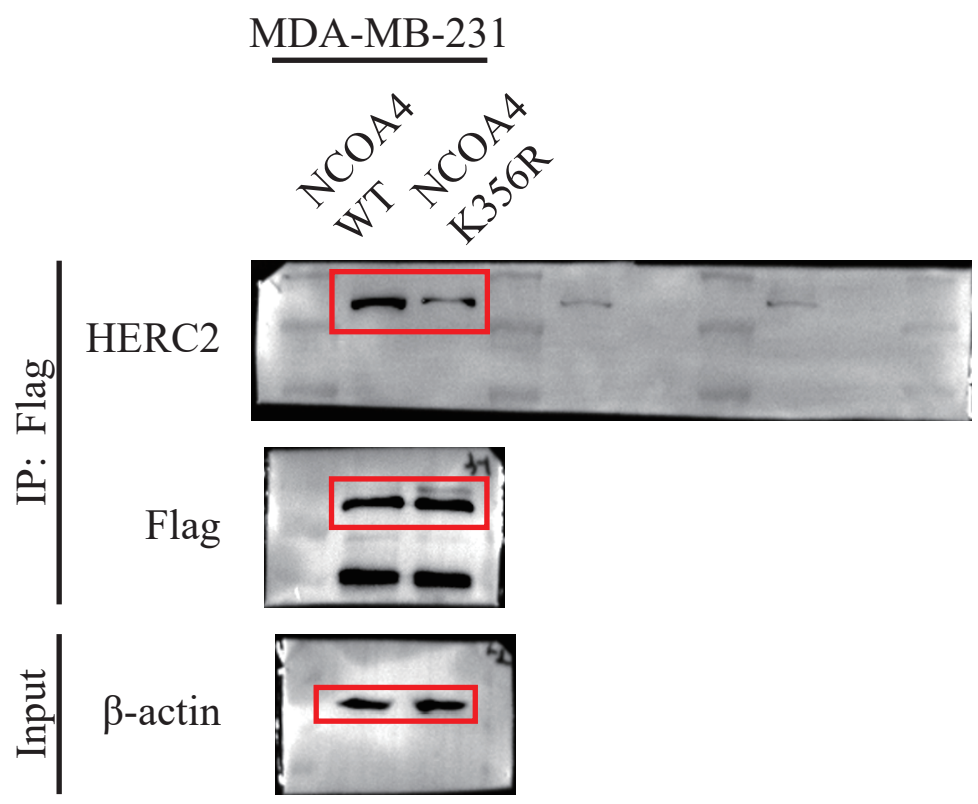

Figure 4

K

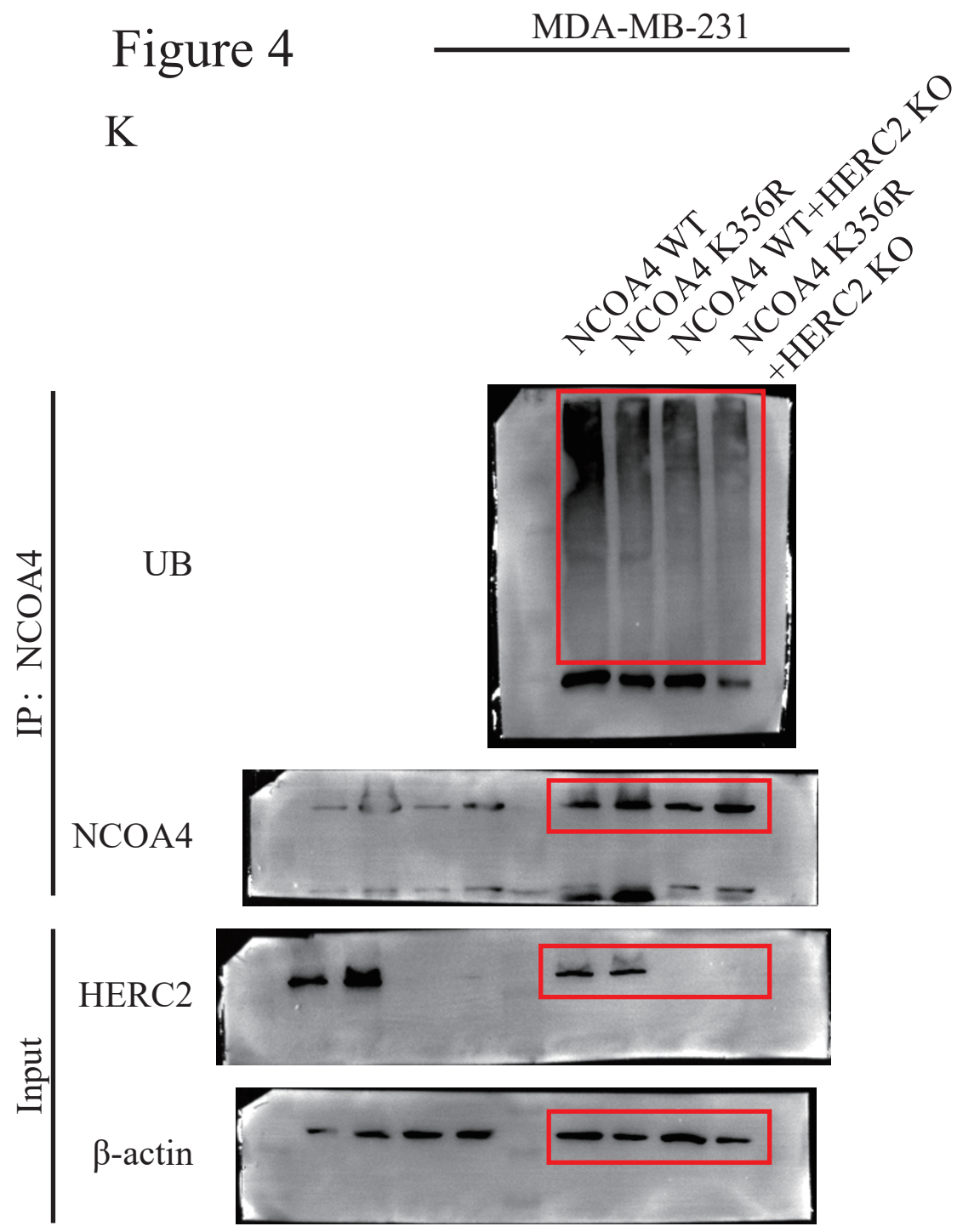

Figure 5

C

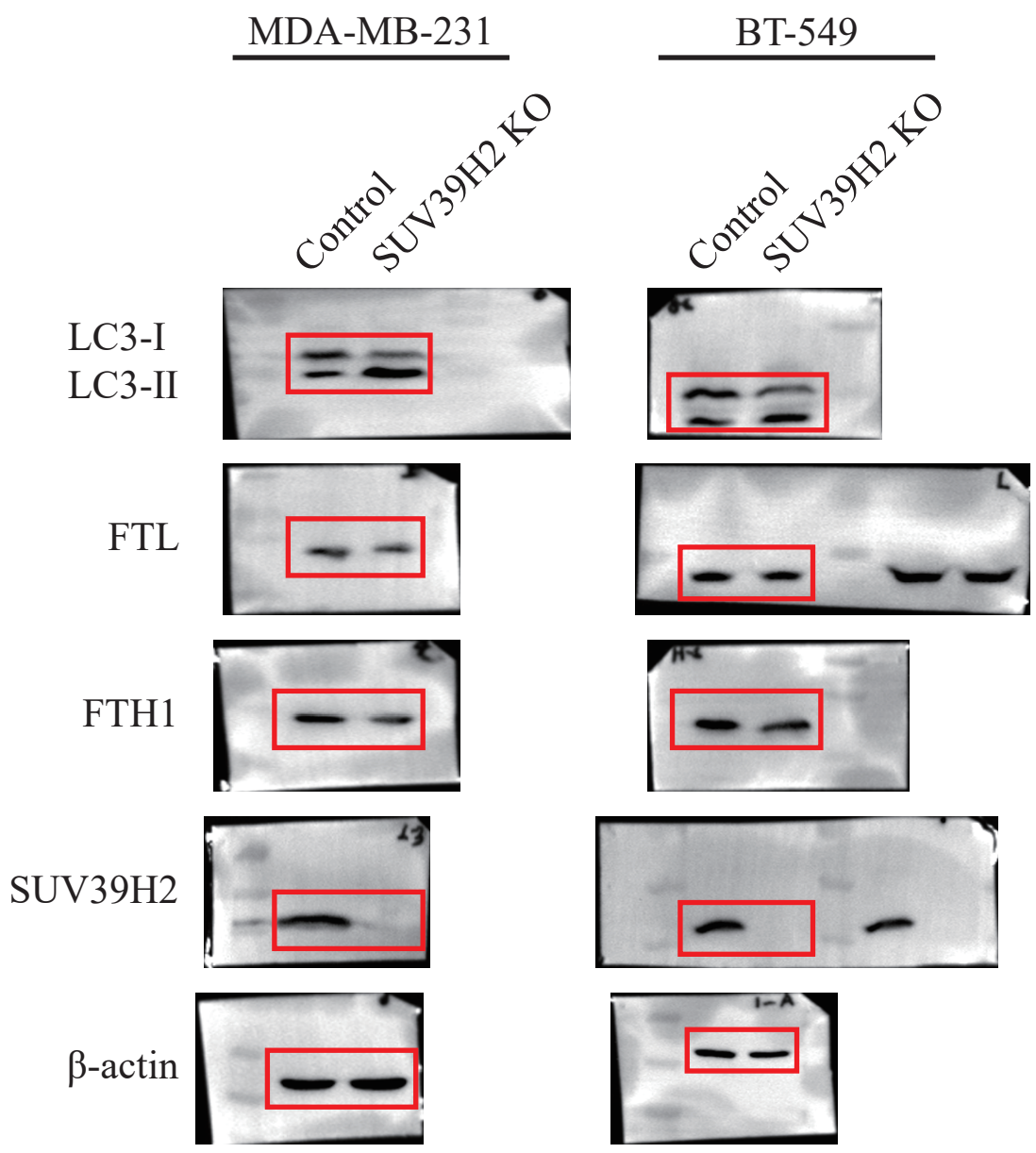

Figure 5

L

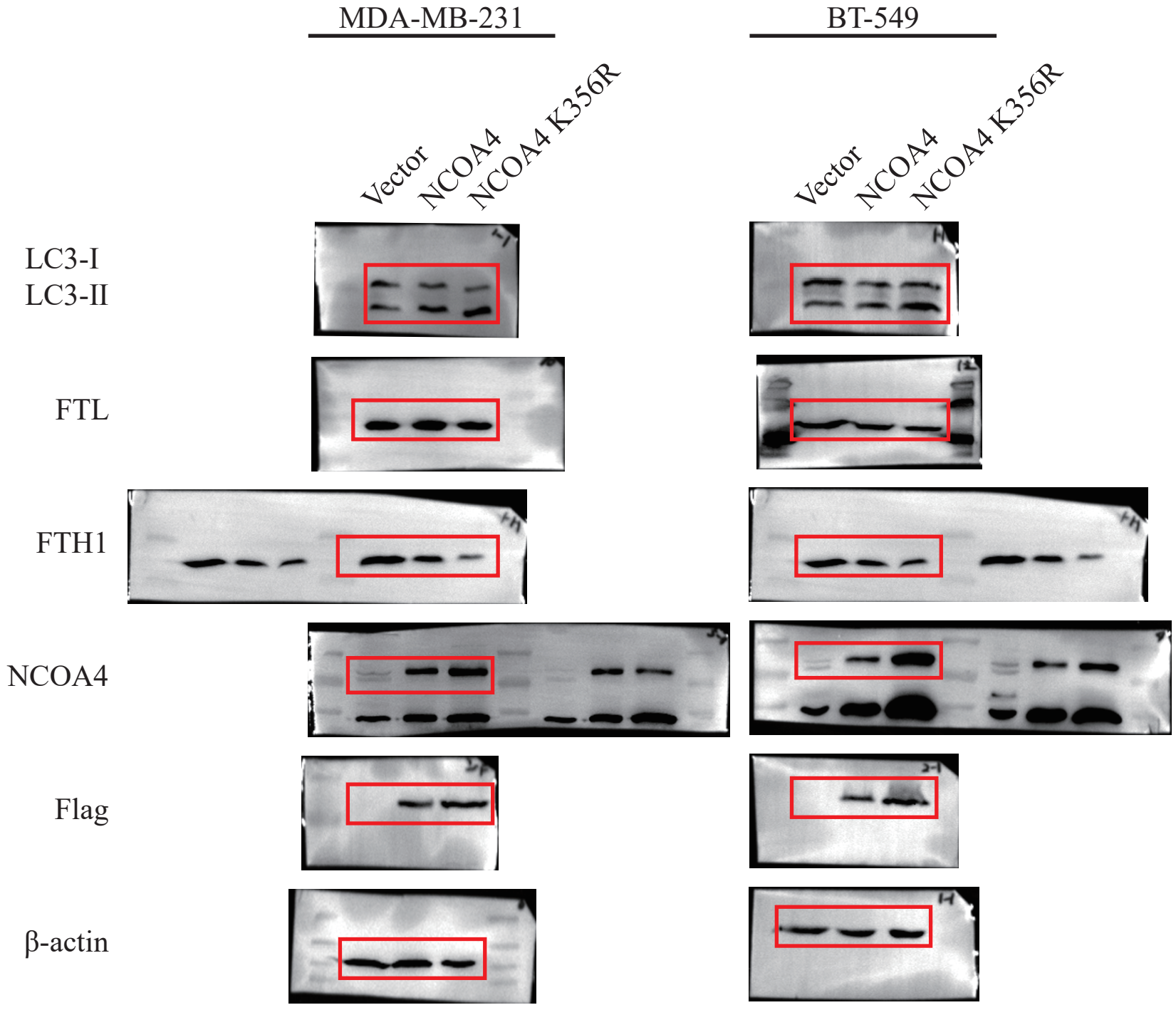

Figure 7

B

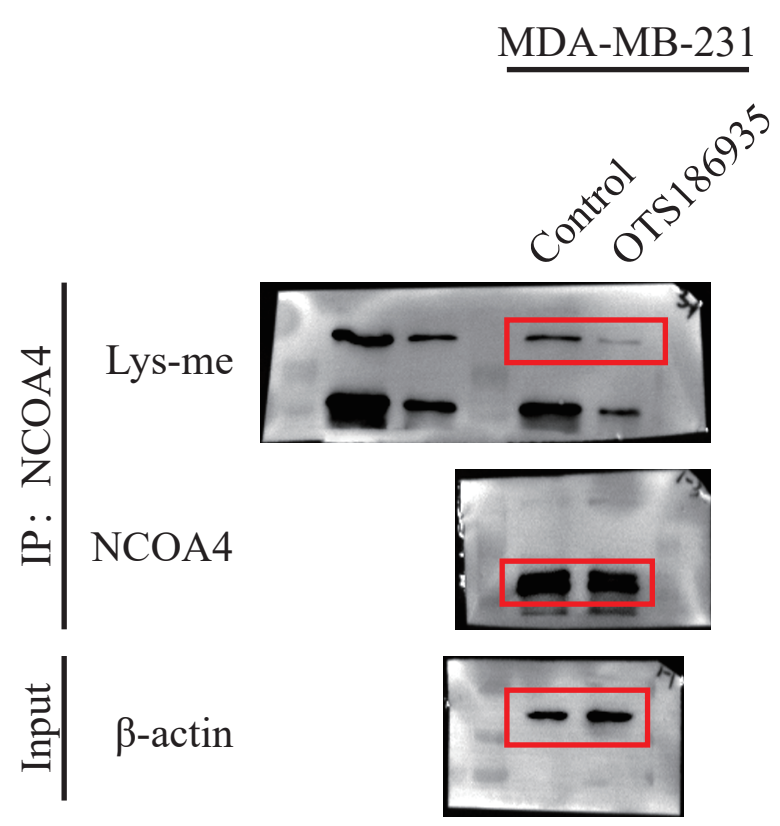

Figure 7

C

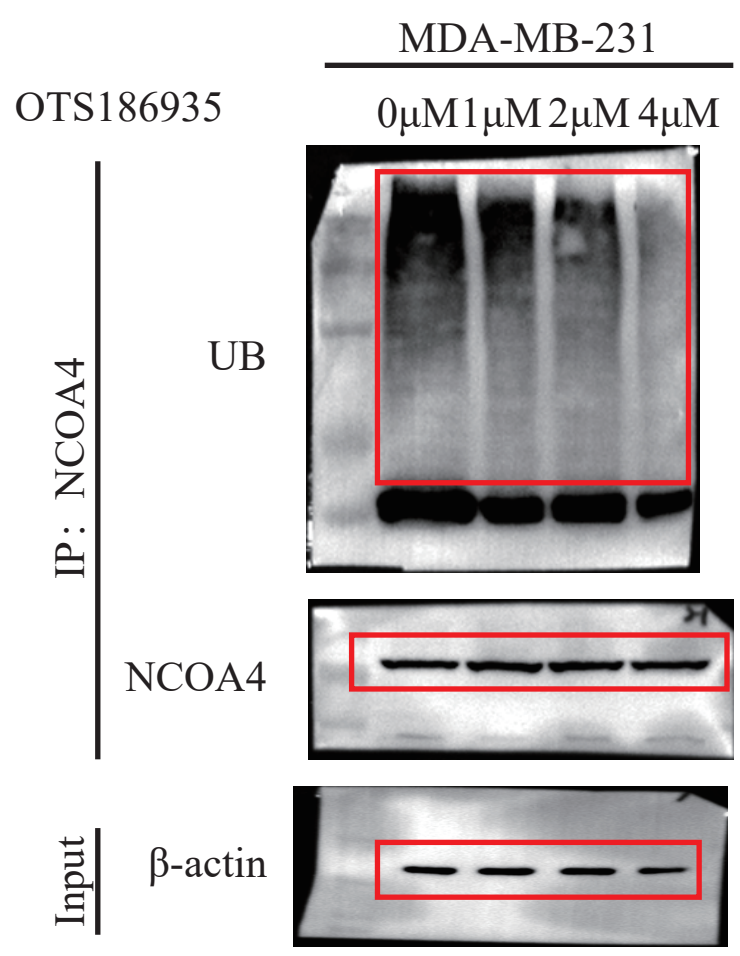

Figure 7

K

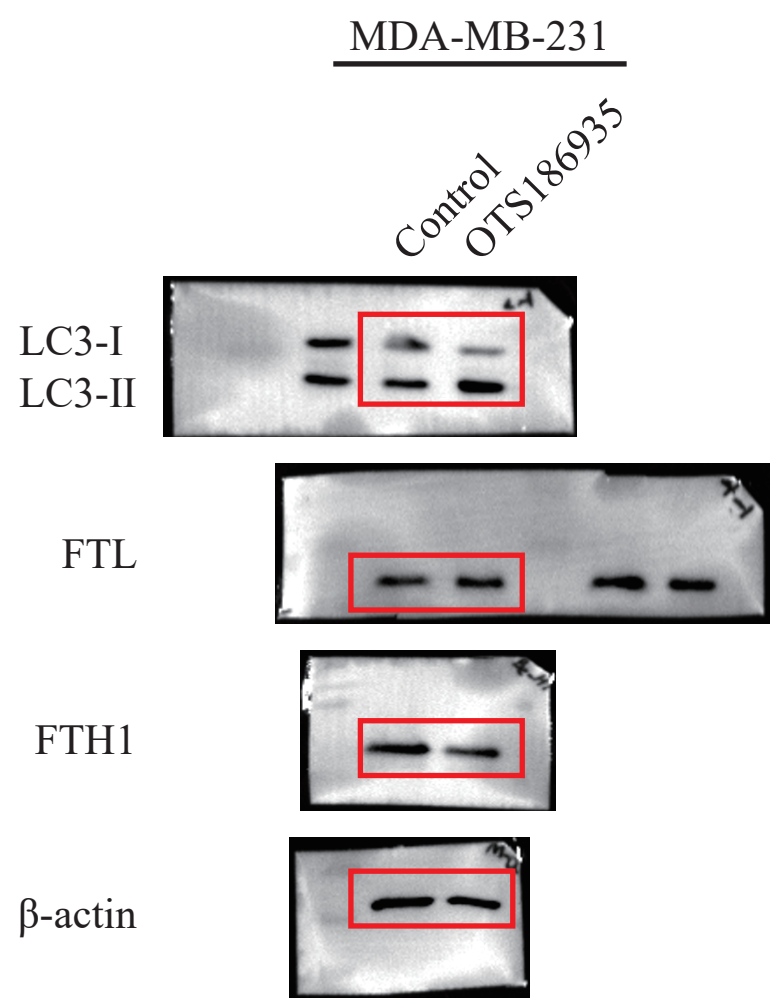

Figure S1

B

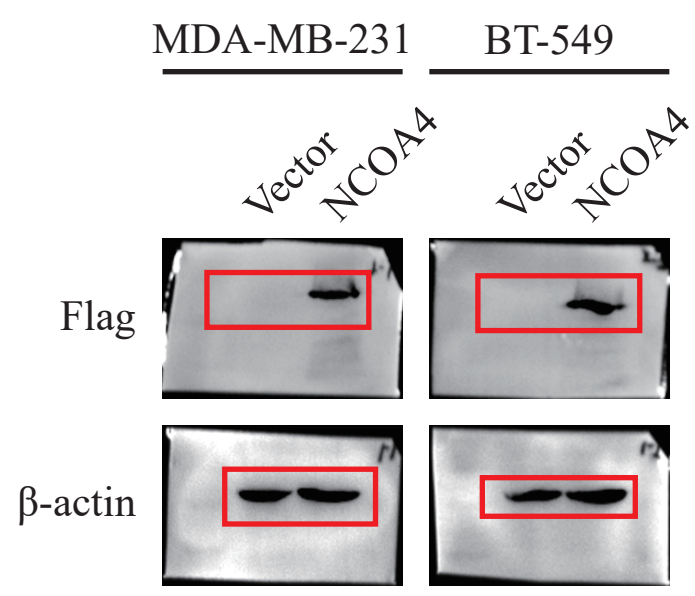

Figure S1

C

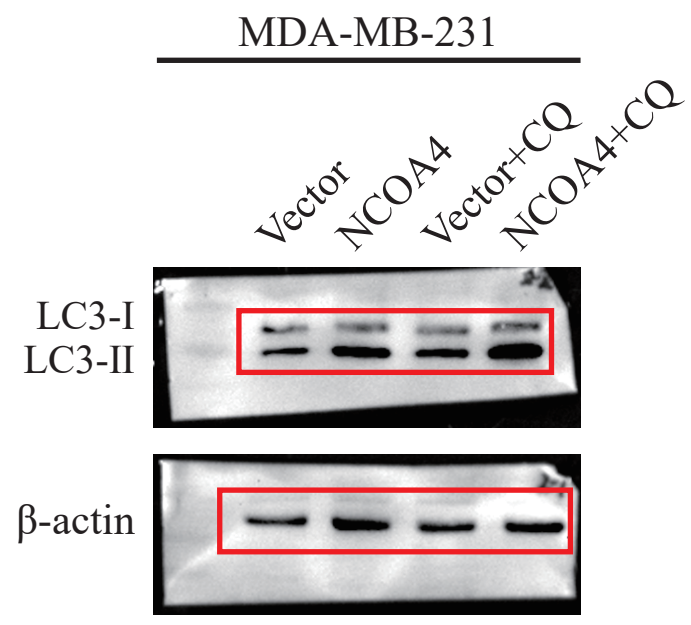

Figure S1

O

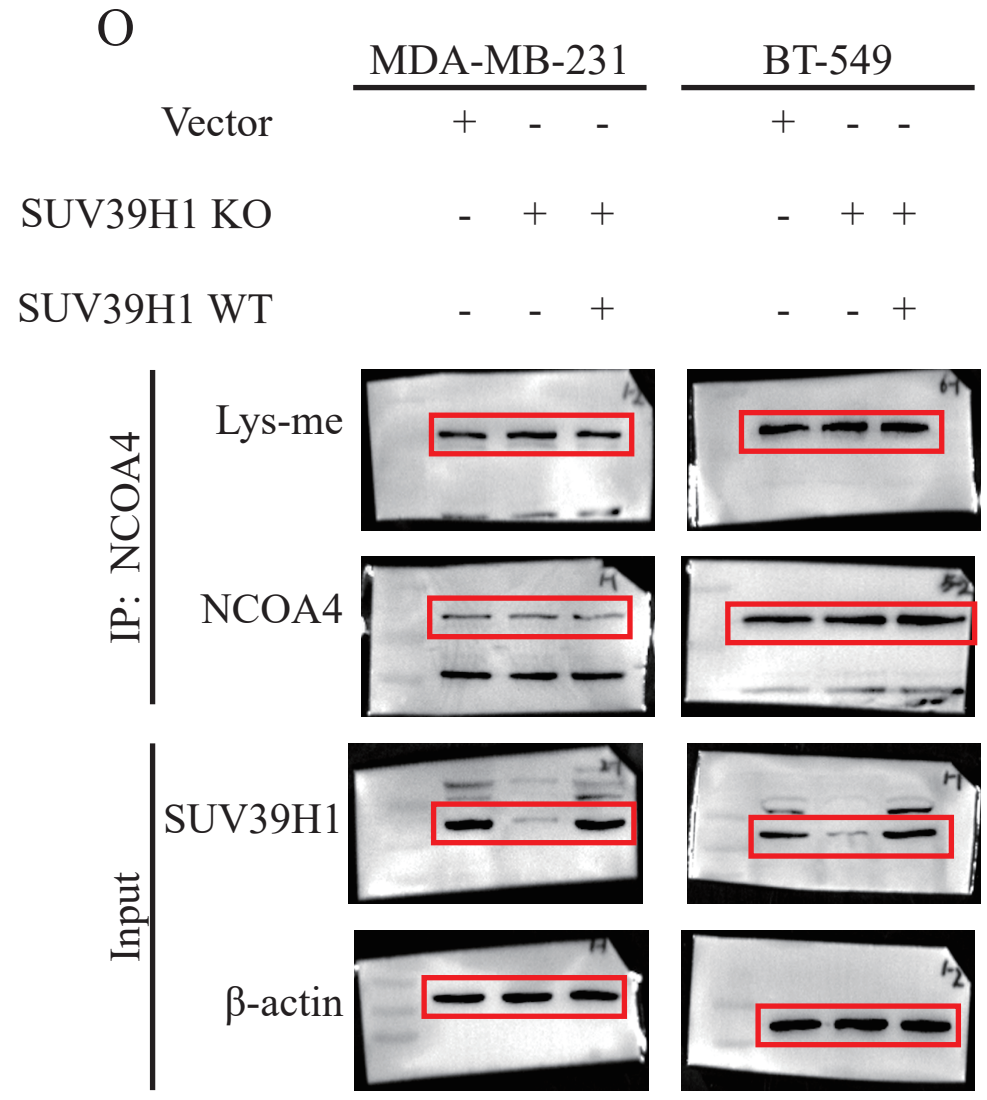

Figure S1

U

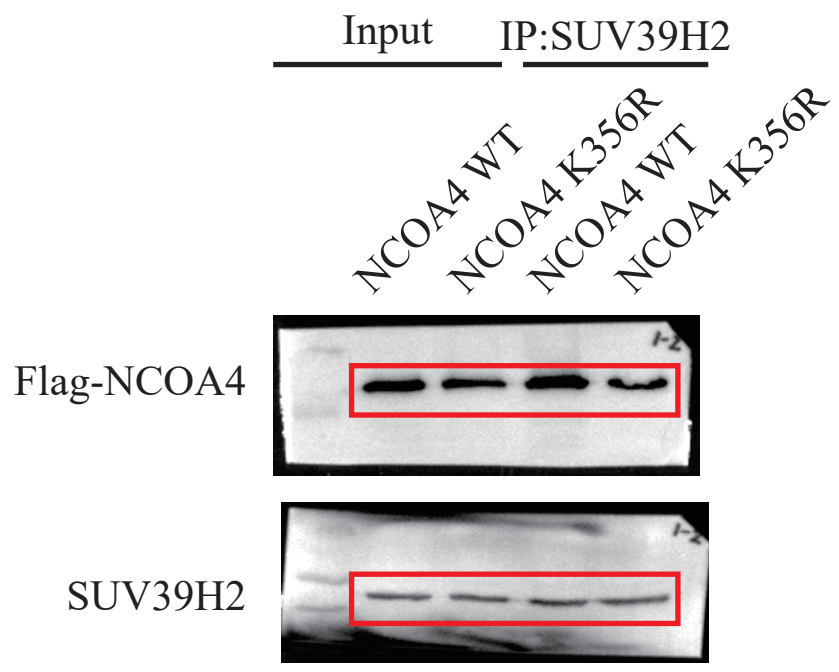

Figure S2

F

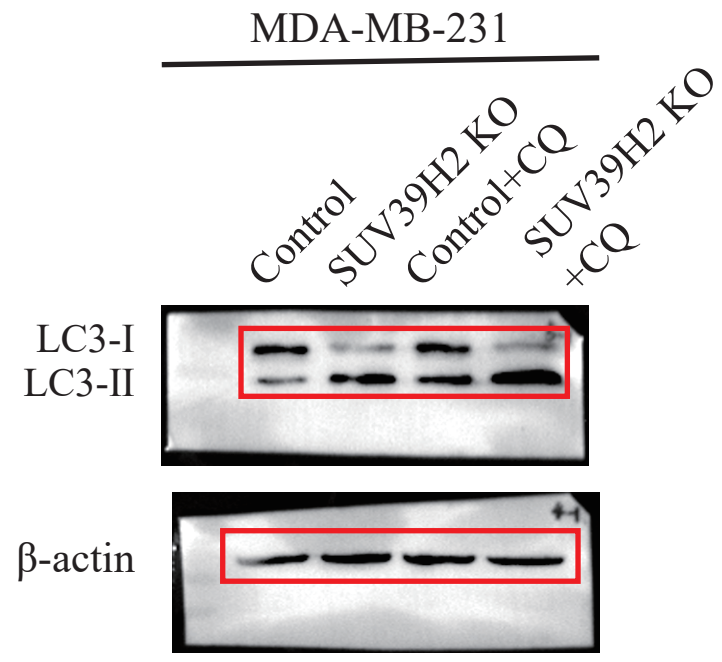

Figure S2

L

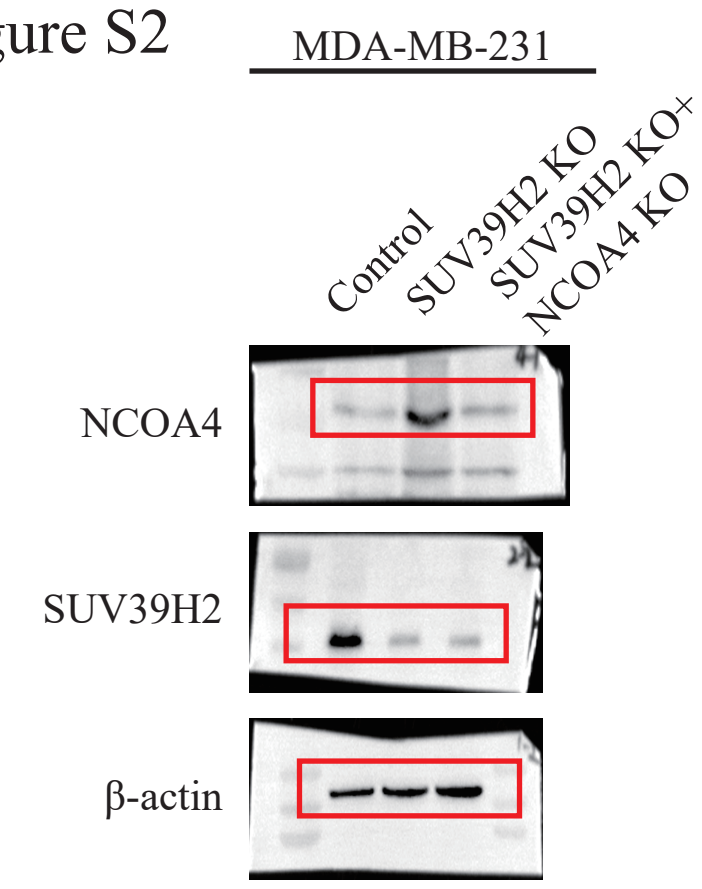

Figure S3

D

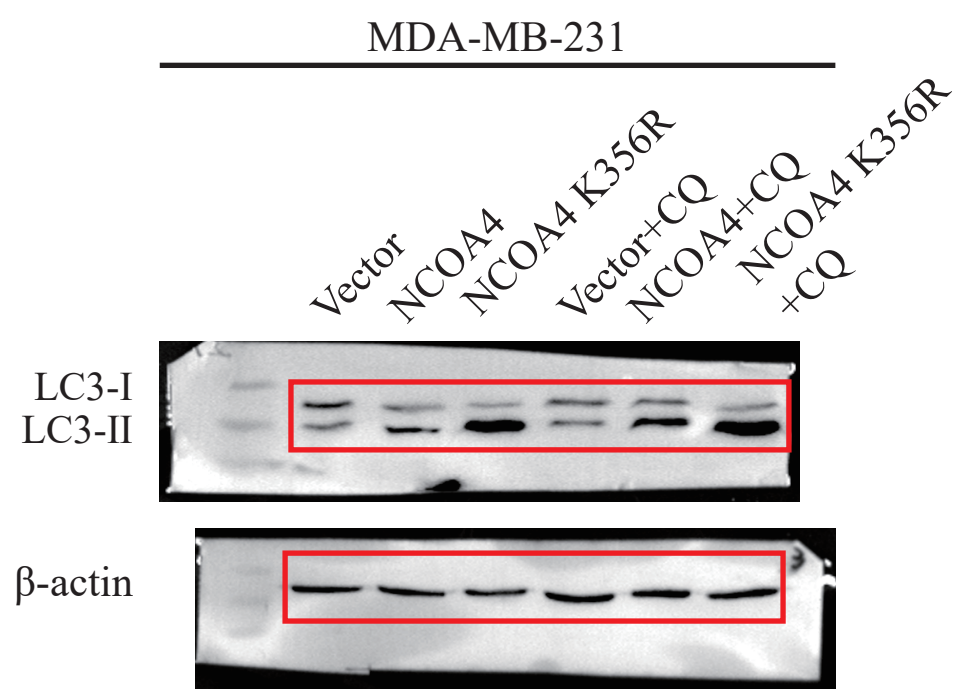

Figure S6

D

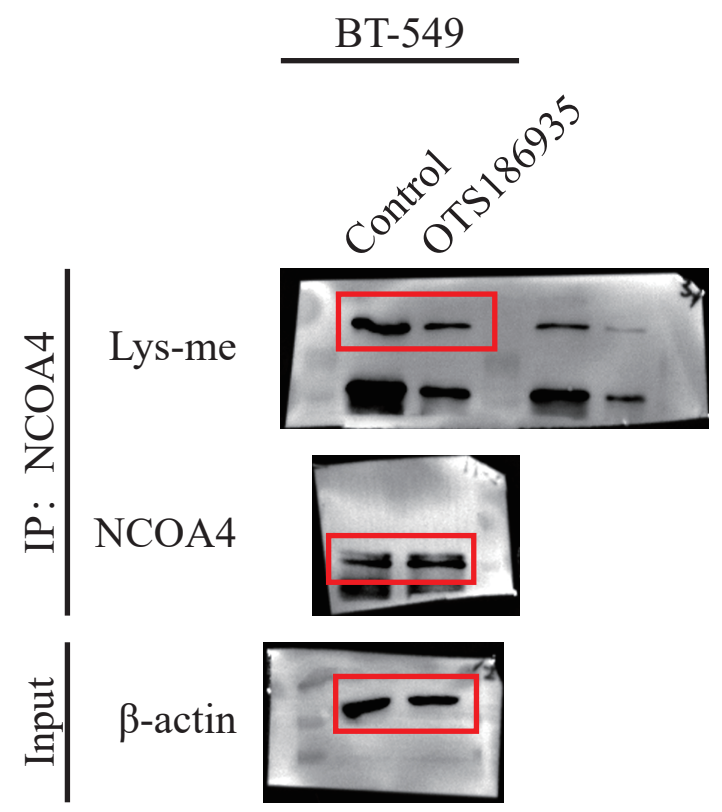

Figure S6

E

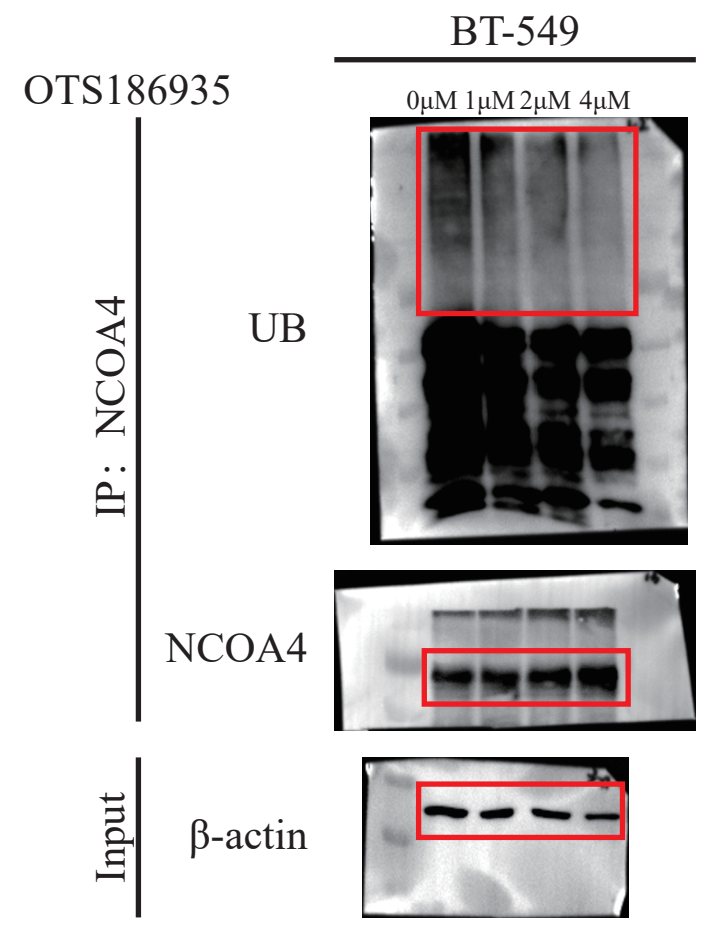

Figure S7

D

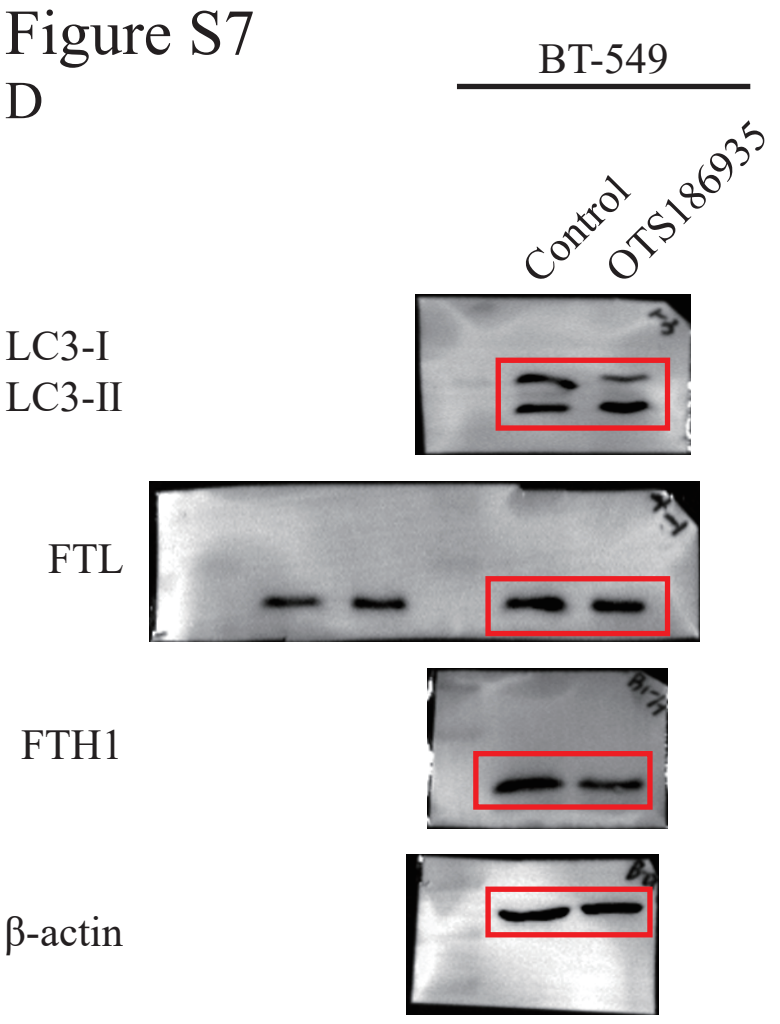

Figure S7

E

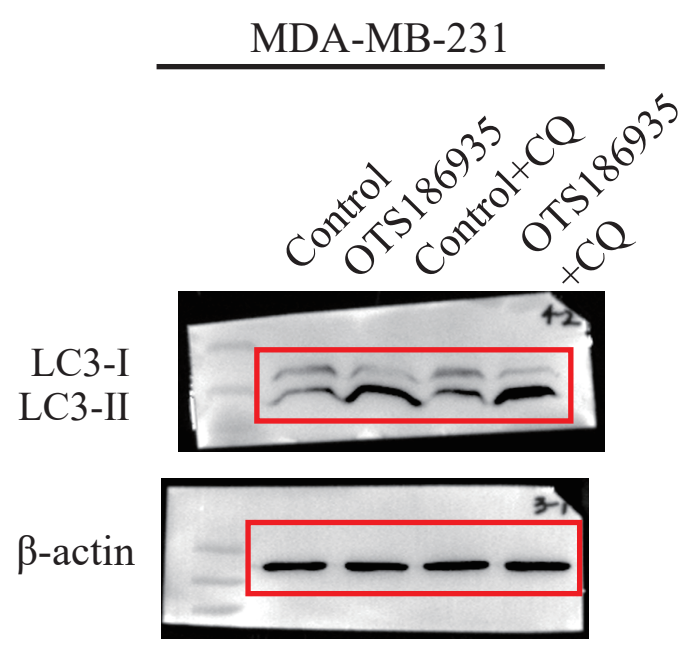

Supplement: Supplementary file 1 [file 41419_2026_9008_MOESM6_ESM.pdf]
