# Supplementary material for: SUV39H2-mediated NCOA4 methylation controls ferritinophagy and ferroptosis in triple-negative breast cancer
Source: Cell Death Dis. 2026 Jul 16;17(1):876. doi: 10.1038/s41419-026-09008-1 (PMC13654947; doi:10.1038/s41419-026-09008-1)
Supplement: Supplementary file 2 [file 41419_2026_9008_MOESM1_ESM.docx]

**Supplementary Figures**

**SUV39H2-mediated NCOA4 methylation** **controls ferritinophagy and ferroptosis in triple-negative breast cancer**

Lingxia Liu^1*^, Xinyun Pei^1*^, Ding Li^2*^, Ying Huo^1^, Zhaoting Wang^1^, Qiu Zhang^1^, Yuan Yue^1^, Bao Deng^3^, Minmin Yu^4^, Tong Xu^1^, Lei Zhong^3#^, Xianhui Ruan^5#^, Xichuan Li^1#^

**Fig. S1. NCOA4 regulates TNBC cell** **ferritinophagy and ferroptosis. A** Quantitative analysis of Fe²⁺ levels in MCF10A and TNBC cells using PGSK fluorescent probe. **B** Western blot confirming NCOA4 overexpression in MDA-MB-231 and BT-549 cells. **C** Western blot analysis of LC3-I/LC3-II protein levels in MDA-MB-231 cells transfected with NCOA4 expression plasmid then treated with CQ (60 µM) for 24h. **D-E** MDA-MB-231-Vector and MDA-MB-231-NCOA4 cells were transiently transfected with a plasmid encoding tandem GFP-mCherry-LC3B, and incubated with 60μM CQ for 24h. GFP and mCherry were visualized by confocal microscopy of live cells. Scale bar, 10 μm. **F** Quantitative analysis of Fe²⁺ levels in NCOA4-overexpressing MDA-MB-231 and BT-549 cells using PGSK fluorescent probe. **G-I** ROS levels in NCOA4-overexpressing cells were detected using DCF fluorescent probes through confocal imaging and flow cytometry. **J, K** Quantitative analyses and flow cytometry of lipid ROS levels in NCOA4 overexpressing cells. **L** Mitochondrial membrane potential (MMP) in NCOA4-overexpressing cells detected using Rhodamine 123 probe. Scale bar, 10 μm. **M** Conservation of NCOA4 K356 across species. **N** AlphaFold-predicted interaction between NCOA4 (turquoise, AlphaFold DB, AF-A0A024QZI5-F1) and SUV39H2 (green, PDB, 6p0r). **O** Detection of NCOA4 methylation in MDA-MB-231 and BT549 cells with SUV39H1 knockout or rescued with SUV39H1. **P** AlphaFold prediction mapping Lys356 is located in the core binding domain of NCOA4 (turquoise, AlphaFold DB, AF-A0A024QZI5-F1) and SUV39H2 (green, PDB, 6p0r). **Q** RMSDs of backbone atoms of proteins during the simulations of the SUV39H2 dimer/NCOA4-M_domain-Wild system (orange) and the SUV39H2 dimer/NCOA4-M_domain-K356R system (blue). **R–T** RMSFs of Cα atoms for NCOA4-M_domain. (R), SUV39H2 monomer 1 (S), and SUV39H2 monomer 2 (T), in the SUV39H2 dimer/NCOA4-M_domain-Wild system (orange) and the SUV39H2 dimer/NCOA4-M_domain-K356R system (blue) during the last 50 ns of MD simulations. **U** Co-IP experiment to detect the interaction between SUV39H2 and NCOA4 WT or NCOA4 K356R mutant. Data are presented as mean ± SD. Comparisons were made using one-way ANOVA with Tukey’s test; **p* < 0.05, ***p* < 0.01, ****p* < 0.001.

**Fig. S2. SUV39H2 inhibits ferroptotic cell death.**

**A** Correlation analysis of NCOA4 and SUV39H2 expression levels was performed using the Pearson correlation coefficient, and visualization was conducted using linear regression. All analyses were performed in R (version 4.2.2). **B-D** The Kaplan-Meier method was used to estimate the survival curves of NCOA4 and SUV39H2, and temporal tests were used to assess differences between the high and low expression groups. Patients were stratified according to expression quartiles (top quartile vs. bottom quartile). All analyses were carried out in R (version 4.2.2), and a two-sided p-value <0.05 was considered statistically significant. **E** Representative images and quantitative analysis of Fe²⁺ in SUV39H2-knockout cells. Scale bar, 100 μm. **F** Western blot detection of changes in LC3I/II in cells with SUV39H2-knockout and CQ treatment. **G-H** GFP and mCherry were visualized by confocal microscopy of MDA-MB-231 cells with SUV39H2 -knockout and CQ treatment. Scale bar, 10 μm. **I-K** Quantitative analysis of ROS, lipid ROS and MMP in SUV39H2 knockout cells. **L** Validation of SUV39H2 and NCOA4 knockout in MDA-MB-231 cells by Western blot. **M** Representative images and quantitative analysis of labile Fe²⁺ in control, SUV39H2 KO and SUV39H2/NCOA4 KO MDA-MB-231 cells. Scale bar, 100 μm. **N** Representative images and quantitative analysis of lipid peroxidation in control, SUV39H2 KO and SUV39H2 KO/NCOA4 KO MDA-MB-231 cells. Scale bar, 100 μm. Data are presented as mean ± SD. Comparisons were made using one-way ANOVA with Tukey’s test; **p* < 0.05, ***p* < 0.01, ****p* < 0.001.

**Fig. S3. NCOA4 K356R mutant enhances ferroptotic cell death.**

**A** Representative images and quantitative analysis of Fe^2+^ in NCOA4 wild-type/K356R overexpressing cells. Scale bar, 100 μm. **B, C** GFP and mCherry were visualized by confocal microscopy of NCOA4 WT/K356R overexpressing cells following treated with CQ. Scale bar, 10 μm. **D** Western blot analysis of changes in LC3 in NCOA4 WT/K356R overexpressing cells treated with CQ. **E, F** Representative images and quantitative analyses of ROS and lipid ROS in NCOA4 WT/K356R overexpression cells. Scale bar, 100 μm. Data are presented as mean ± SD. Comparisons were made using one-way ANOVA with Tukey’s test; **p* < 0.05, ***p* < 0.01, ****p* < 0.001.

**Fig. S4. NCOA4 K356 methylation promotes TNBC cell migration and invasion. A** Representative images and quantitative analyses of colony formation assay in NCOA4-overexpressing MDA-MB-231 and BT-549 cells. **B** Growth curves of NCOA4-overexpressing MDA-MB-231 and BT-549 cells. **C** Representative images and quantitative analyses of Edu assay in NCOA4-overexpressing MDA-MB-231 and BT-549 cells. **D** Representative images of adhesion assay. Scale bar, 100 μm. **E** Invasion and migration assays. Representative images (left) and quantification (right). Scale bar, 100 μm. **F-G** scratch assays, migration and invasion under SUV39H2 KO conditions. Scale bar, 100 μm. **H-J** scratch assays, migration and invasion under NCOA4 WT/K356R overexpression conditions. Scale bar, 100 μm. Data are presented as mean ± SD. Comparisons were made using one-way ANOVA with Tukey’s test; **p* < 0.05, ***p* < 0.01, ****p* < 0.001.

**Fig. S5. Prediction and experimental validation of chemotherapy drug IC50.**

**A** CCK8 assay to detect the IC50 of four chemotherapy drugs. **B** Using the PRROPHIC R package to predict the half-maximal inhibitory concentration (IC50) of chemotherapy drugs, which is the drug concentration required to inhibit 50% of cell growth. Data are presented as mean ± SD. Comparisons were made using one-way ANOVA with Tukey’s test; **p* < 0.05, ***p* < 0.01, ****p* < 0.001.

**Fig. S6. OTS186935 strengthens ferroptosis.**

**A** The Venn diagram shows the intersection of DEGs in MDA-MB-231 cells with either SUV39H2 knockout or treatment with the inhibitor OTS186935. **B** The top 50 genes in the intersection were selected for heat mapping. Row-wise z-score scaling was used to emphasize the relative expression changes. Generate heat maps using the pheatmap package (version 1.0.13). **C** CCK8 assay detecting the IC50 of OTS186935 in BT-549 cells. **D** Immunoprecipitation assay detecting NCOA4 methylation levels in OTS186935-treated BT-549 cells. **E** Immunoprecipitation assay detecting NCOA4 ubiquitination levels under OTS186935 treatment. **F** Analysis of cell viability in BT-549 cells after OTS186935 treatment combined with ferroptosis inhibitors (DFO, Fer-1, NAC). **G-J** Representative images and quantitative analyses of ROS, lipid ROS, MMP and TEM in OTS186935-treated BT-549 cells. Data are presented as mean ± SD. Comparisons were made using one-way ANOVA with Tukey’s test; **p* < 0.05, ***p* < 0.01, ****p* < 0.001.

**Fig. S7. OTS186935 enhances ferritinophagy and inhibits the proliferation of TNBC cells.**

**A-C** Representative images and quantitative analyses of Fe^2+^ in OTS186935-treated BT-549 cells. **D** Western blot analysis of LC3-I/II, FTL and FTH1 protein levels in OTS186935-treated BT-549 cells. **E** Western blot analysis of LC3-I/II protein levels in MDA-MB-231 cells treated with OTS186935 or in combination with CQ. **F, G** Representative images and quantitative analysis of autophagosome and autolysosome formation in MDA-MB-231 cells treated with OTS186935 or in combination with CQ. Scale bar, 10 μm. **H-J** Representative images and quantitative analyses of colony formation assay in OTS186935-treated BT-549 cells. **K** Growth curves of OTS186935-treated BT-549 cells. **L** Representative images and quantitative analyses of EdU assay in OTS186935-treated BT-549 cells. Scale bar, 100 μm. Data are presented as mean ± SD. Comparisons were made using one-way ANOVA with Tukey’s test; **p* < 0.05, ***p* < 0.01, ****p* < 0.001.

**Fig. S8. OTS186935 inhibits the invasion and migration of TNBC cells and shows no significant toxicity in mice.**

**A-F** Scratch assays, migration and invasion in OTS186935-treated MDA-MB-231/BT-549 cells. Scale bar, 100 μm. **G** Representative images of adhesion assay in OTS186935-treated MDA-MB-231 and BT-549 cells. Scale bar, 100 μm. **H** Cell viability of OTS186935 combined with chemotherapeutic drugs in BT-549 cells.

**I** Detect the levels of ALT, AST, CREA and BUN in the serum of administered mice. **J** Representative images of HE-stained mouse lung, heart, liver, spleen and kidney. Scale bar, 50 μm. Data are presented as mean ± SD. Comparisons were made using one-way ANOVA with Tukey’s test; **p* < 0.05, ***p* < 0.01, ****p* < 0.001.
