# Supplementary material for: SUV39H2-mediated NCOA4 methylation controls ferritinophagy and ferroptosis in triple-negative breast cancer
Source: Cell Death Dis. 2026 Jul 16;17(1):876. doi: 10.1038/s41419-026-09008-1 (PMC13654947; doi:10.1038/s41419-026-09008-1)
Supplement: Supplementary file 3 [file 41419_2026_9008_MOESM2_ESM.docx]

**Supplementary Materials and Methods**

**SUV39H2-mediated NCOA4 methylation controls** **ferritinophagy and ferroptosis in triple-negative breast cancer**

Lingxia Liu^1*^, Xinyun Pei^1*^, Ding Li^2*^, Ying Huo^1^, Zhaoting Wang^1^, Qiu Zhang^1^, Yuan Yue^1^, Bao Deng^3^, Minmin Yu^4^, Tong Xu^1^, Lei Zhong^3#^, Xianhui Ruan^5#^, Xichuan Li^1#^

**Antibodies and reagents**

**Antibody**

Anti-NCOA4 antibody (Abcam, ab314553; ABclonal, A5695). Anti-KMT1B/SUV39H2 antibody (Abcam, ab190870; ABclonal, A3705). Ubiquitin Rabbit mAb (ABclonal, A19686). Anti-Methylated Lysine (dimethyl, mono methyl) antibody (Abcam, AB23366). Ferritin Heavy Chain Rabbit mAb (ABclonal, A19544). Ferritin Light Chain Rabbit mAb (ABclonal, A18051). LC3B (Sigma-Aldrich, L7543). Ki-67 (Cell Signaling Technology, D2H10). DDDDK-Tag Rabbit mAb (ABclonal, AE063). DYKDDDDK tag Monoclonal antibody (Proteintech, 66008-4-Ig). Rabbit Control IgG (ABclonal, AC005). β-actin Rabbit mAb (ABclonal, AC038). Goat Anti-Rabbit IgG(H+L) (ABclonal, AS070). PRSPURE HRP-conjugated goat anti-rabbit IgG H&L (ANOLA, AN19618). Goat Anti-Mouse IgG H&L, Cy5 conjugated (Bioss Antibodies, bs-0296G-Cy5). Goat Anti-Rabbit IgG H&L, FITC conjugated (Bioss Antibodies, bs-0295G-FITC). LAMP2 Mouse Monoclonal Antibody (HUABIO, EM1901-62).

**Reagent**

GST-tagged protein purified agarose magnetic beads (Beyotime, P2258-2 ml). DMEM (Gibco, C11995500BT). Complete MCF10A culture medium (Wuhan Pricella Biotechnology Co., Ltd, CM-0525). Cell Counting Kit-8 (CCK-8, Biosharp, BS350B）. IPTG solution (Sangon Biotech, B541007). Cycloheximide (CHX, Glpbio, GC17198, 100μg/mL). MG-132 (MCE, HY-13259, 10μM). OTS186935 (MCE, HY-122181B, purity was 99.58%). Chloroquine (CQ, TargetMol, T8689, purity was 99.98%). Cisplatin injection (DDP, Nuoxin, H20040813). Paclitaxel (PTX, Solarbio, SP8020). Doxorubicin (ADM, Aladdin, A183027-25 mg). Capecitabine (NVB, Solarbio, A8740). Deferoxamine mesylate (DFO, MCE, HY-B0988). Ferrostatin-1 (Fer-1, MCE, HY-100579). Acetylcysteine (NAC, MCE, HY-B0215). BeyoClick™ EdU-555 Cell Proliferation Assay Kit (Beyotime, C0075S). BODIPY 581/591 C11（Thermo Fisher Scientific，D3861）. Reactive Oxygen Species Detection Kit (Beyotime, S0033S). Phen Green SK diacetate (MCE, HY-126823). Cell Ferrous Iron Colorimetric Assay Kit (Elabscience, E-BC-K881-M). Ferrous Iron Colorimetric Assay Kit (Elabscience, E-BC-K773-M). Rhodamine 123（Beyotime, C2007）. Poly-D-sinewy (Beyotime, ST508). Rapid Silver Staining Kit (Beyotime, P0017S). Prussian blue staining kit (Solarbio, G1428). Fibronectin (Solarbio, F8180). Hematoxylin (Solarbio, H8070). Eosin Y Stain Solution, For HE (Solarbio, G1100). Alanine aminotransferase (ALT) Assay Kit, Creatinine (CRE) Assay kit (sarcosine oxidase), Aspartate aminotransferase (AST) Assay Kit, Blood Urea Nitrogen (BUN) Assay Kit (Nanjing Jiancheng Bioengineering Institute, C009-2-1, C011-2-1, C010-2-1, C013-2-1).

**Plasmids and viral infection**

3×Flag-NCOA4, 3×Flag-SUV39H2, 3×Flag-NCOA4-K356R plasmids were constructed by Quanyang organisms and these plasmids were used to generate GST-NCOA4 (pGEX-6P-1-NCOA4), GST-NCOA4-K356R (pGEX-6P-1-NCOA4-K356R), His-SUV39H2 (PET-28a-SUV39H2) and His-SUV39H2 mutant (PET-28a-SUV39H2 mutant). SUV39H2 KO，HERC2 KO, SUV39H1 KO and NCOA4 KO plasmids were purchased from Beyotime Biotechnology Co., Ltd. (Shanghai, China). The mCherry-EGFP-LC3B plasmid was the gifts from Dr. Cong Lin (Changchun Institute of Applied Chemistry Chinese Academy of Sciences).

The gRNA sequences used for CRISPR/Cas9 knockout of SUV39H2, HERC2, SUV39H1 and NCOA4 were as follows:

human SUV39H2: GGACAGTTCTATGACAACAA

human HERC2: TAGGAGGGAGCTCTCCGTTC

human SUV39H1: GTTCCTCTTAGAGATACCGA

human NCOA4: CAGTTGCATAAGCCGTCACC

The lentivirus packaging vectors used were packaging plasmid psPAX2 (Addgene, Cambridge, MA, USA) and envelop plasmid pMD2.G (Addgene). The transfer vectors together with the previous two plasmid transfected into HEK-293T cells in a suitable ratio mixed with transfection reagent polyethylenimine (PEI, Sigma-Aldrich). The supernatant was filtered through a 0.45-μm filter (Millipore, Temecula, CA, USA) after collected in 48 h, and the concentrated virus was then infected recipient cell lines with 5 mg/mL polybrene.

**GST pulldown assay and in vitro methylation assay**

Briefly, GST or GST fusion proteins were expressed in bacteria BL21 (Sangon Biotech, B528414-0010) induced with 0.1 mM isopropyl-β-D-thio-galactoside (IPTG) for 4 h at 37 °C and then purified on glutathione-Sepharose 4B (GE Healthcare). His fusion proteins protein was also expressed in BL21 induced with 0.1 mM IPTG and then purified on Prot Elut Ni according to the manufacturer's instructions (Enriching Biotechnology).

For GST pulldown assay, GST fusion protein-adsorbed beads are incubated with cell lysate from MDA-MB-231 at 37 °C for 1 h. Beads were washed 3 times with 1 mL of GST lysate, with a 100 μL protein buffer metal bath for 10 min, immunoblotting analysis, and Coomassie brilliant blue staining. The protein samples were placed in a 10% sodium dodecyl sulfate-polyacrylamide gel electrophoresis (SDS-PAGE) gel, and after electrophoresis, the gel was stained with Coomassie brilliant blue on the protein band for 10 min. Soak in distilled water overnight.

For in vitro methylation, the purified enzyme (His-SUV39H2) and substrate (GST-NCOA4) were purified in S-adenosyl-methionine (SAM; 15 Ci/mmol; Perkin Elmer, USA) was incubated at 30 °C for 1 h and then western blot analysis was performed with lysine pan-methylated antibody.

**Mass spectrometry analysis**

For identification of NCOA4-binding proteins, Flag-NCOA4 protein was purified from HEK293T-3×Flag-NCOA4 cells and then detected by SDS-PAGE. Stained with Coomassie Brilliant Blue (CBB). The whole gel was analyzed by liquid chromatography-tandem mass spectrometry (LC-MS/MS) in the Academy of Biological Sciences Proteomics Research and Analysis Center, Shanghai.

For identification of lysine methylation site, 3×Flag-NCOA4 protein was purified from HEK-293T cells and then detected by SDS-PAGE. Stained with Coomassie Brilliant Blue (CBB). The 3×Flag-NCOA4 band was excised and analyzed by liquid chromatography-tandem mass spectrometry (LC-MS/MS) in the Academy of Biological Sciences Proteomics Research and Analysis Center, Shanghai.

**Immunoprecipitation**

For immunoprecipitation, cells were harvested and lysed in Buffer A including a protease inhibitor cocktail tablet (Roche) for 30 min at 4 °C. Total protein lysates were incubated overnight with corresponding antibodies with gentle shacking at 4 °C, followed by addition of 30-50 μL of Pure Proteome Protein A/G Mix Magnetic Beads for another 2 h at room temperature. The beads were washed 3 times with Buffer A, resuspended in 60-100 μL of 2×loading buffer and boiled for 10 min. Then the supernatant was subjected to SDS-PAGE, transfered onto a PVDF membrane and detected by ECL reagents.

**Immunofluorescence**

Used 0.1 mg/mL of polylysine overnight to coat the cell crawl slides. After the crawl slide was dry, the cells were seeded onto the crawl slide. Then, the cells were fixed with absolute ethanol for 10 min and washed 3 times in PBS. Permeabilized with PBS containing 0.3% Triton X-100 for 15 min at room temperature and washed 3 times in PBS. The coverslips were blocked with 10% goat serum for 30 min. Wash 3 times in PBS and the cells were incubated with the primary antibody at 4 °C overnight. After washing the coverslips thrice with PBS, the cells were incubated with secondary antibodies for 1 h and DAPI for 15 min at room temperature. Taked pictures using a confocal microscope.

**Molecular docking**

The crystal structure of SUV39H2 was obtained from the Protein Data Bank (PDB code: 6P0R; http://www.rcsb.org/). The three-dimensional structure of NCOA4 was acquired from the AlphaFold Protein Structure Database (https://alphafold.ebi.ac.uk/). Protein-protein interactions were modeled using AlphaFold3 (https://alphafoldserver.com/) to predict the optimal docking sites between SUV39H2 and NCOA4, and visualized using PyMOL (version 1.8.2.0; DeLano Scientific, LLC).

**System sets**

The structure of the SUV39H2 dimer in complex with the middle domain of NCOA4 (NCOA4-M_domain) was generated using AlphaFold. The CHARMM-GUI web server [1] was employed to construct the molecular dynamics simulation systems. The SUV39H2 dimer/NCOA4-M_domain complex was centered in a 13 × 13 × 13 nm³ TIP3P water box containing 0.15 mol·L⁻¹ NaCl to mimic physiological ionic conditions [2], yielding the SUV39H2 dimer/NCOA4-M_dimain-Wild system. To investigate the effect of residue substitution, Lys356 of NCOA4 was manually mutated to Arg, generating the SUV39H2 dimer/NCOA4-M_dimain-K356R system.

**Molecular dynamics simulations**

All molecular dynamics (MD) simulations were performed using GROMACS 2023 with periodic boundary conditions [3]. The CHARMM36m force field was employed for proteins and ions [4]. The LINCS algorithm was used to constrain all bonds involving hydrogen [5]. Van der Waals interactions were switched and cut off at 1.0 nm and 1.2 nm. Long-range electrostatic interactions were handled using the particle-mesh Ewald (PME) method with a cut-off value of 1.2 nm [6]. Pressure and temperature were maintained at 1 atm and 310.15 K using the Parrinello-Rahman and Nosé-Hoover temperature coupling algorithms [7]. Each system underwent initial energy minimization using the steepest descent algorithm and was subsequently equilibrated for 2 ns. Harmonic constraints were applied to the heavy atoms of the protein, with a force constant of 956.0 kcal/(mol*nm²) for the backbone and 478.0 kcal/(mol*nm²) for the side chain at the beginning of equilibration. The constraints were gradually decreased to zero during the equilibration phase. After this, 200 ns MD simulations were performed for each system under the isothermal-isobaric ensemble (NPaT).

The root-mean-square deviations (RMSDs) and root-mean-square fluctuations (RMSFs) were calculated through internal modules of GROMACS. Protein-ligand hydrogen bond interactions were analyzed by ProLIF-2.0.1[8]. VMD-1.9.4[9] and PyMol-2.6.0[10] were used to visualize the trajectories of the simulation.

**Gene expression correlation analysis**

Pearson correlation coefficient was used to analyze the correlation of gene expression levels, and linear regression was used to visualize. All analyses were performed in R (version 4.2.2).

**Survival Analysis**

Kaplan Meier method was used to estimate the survival curve, and the difference between high expression group and low expression group was evaluated by by the log-rank test. Patients were stratified according to the expression quartiles (upper 25% vs lower 25%). All analyses were performed in R (version 4.2.2), and bilateral P values<0.05 were considered statistically significant.

**Prediction of IC50 for Chemotherapy Drugs**

The 50% maximum inhibitory concentration (IC50) of chemotherapeutic drugs was predicted by pRRophetic R software package.

**RNA sequencing**

Total RNA isolated from normal, SUV39H2-knockout, and OTS186935-treated MDA-MB-231 cells was subjected to RNA sequencing at Novogene. The overlap between different gene sets was displayed by Venn diagram, and intersections between gene sets was calculated for visualization. The Venn diagram is generated using the VennDiagram package(version1.8.2). Generate heat maps to visualize gene expression patterns. The normalized read counts was obtained using variance stabilizing transformation (VST) in the DESeq2 package (version 1.48.1). The top 50 genes in the intersection were selected for heat mapping. Row-wise z-score scaling was used to emphasize the relative expression changes. Generate heat maps using the pheatmap package (version 1.0.13)

**Iron content determination**

An Iron Assay Kit was used to measure the Ferrous Iron content of cells. Follow the manufacturer’s protocols, collected the cells, and added the lysate. The supernatant was centrifuged, and the sample was added to a 96-well plate according to the kit system. Finally, the absorbance was measured at 593 nm (A593).

An Iron Assay Kit was used to measure the Ferrous Iron content of TNBC tissues. Take a measured amount of tissue, cut fresh tissue blocks, add the extraction buffer according to the manufacturer’s protocols to homogenize, centrifuge at 12,000 × g for 10 minutes, and collect the supernatant for later use. The supernatant was added to the 96-well plate according to the manufacturer’s protocols and the absorbance was measured at 593 nm.

**Measurement of ROS and lipid ROS**

**Measurement of ROS**

DCFH-DA was diluted 1:1000 in serum-free DMEM, resulting in a final concentration of 10 μM. Cells were incubated with the staining solution for 20 min at 37 °C. After incubation, the cells were washed three times with serum-free DMEM to remove excess DCFH-DA. ROS levels were then quantified using flow cytometry and visualized via confocal microscopy.

**Measurement of lipid ROS**

Cells were seeded in 24-well plates and treated according to the indicated experimental conditions. After treatment, the medium was removed, and cells were incubated with 5 μM BODIPY 581/591 C11 in serum-free medium. Lipid ROS levels were quantified using a 488 nm laser on a flow cytometer or visualized via super-resolution microscopy.

**Transmission electron microscopy**

Cells were fixed with 2.5% glutaraldehyde in phosphate buffer and subsequently treated with 1% OsO_4_ for 2 h. After dehydration, the cells were embedded in epoxy resin. Ultrathin sections were prepared using an ultramicrotome, stained with lead citrate and uranyl acetate, and the distribution of mitochondria was observed using a trans mission electron microscope.

**Cell viability assay**

Cell viability was measured using the CCK-8 kit. MDA-MB-231 and BT-549 cells are seeded in 96-well plates at a density of 3000 or 5000 cells per well and then treated with test compounds. At the indicated times, add 10μL CCK-8 solution to each well of the plate. After incubation for 2h, the absorbance of the plate was measured at 450 nm. Cell viability under the indicated conditions is shown as a percentage relative to the negative control.

**Clone formation assay**

Cells were seeded at 1000 per well in 6-well plates and incubated overnight at 37 ℃. After 7 days, colony cell growth was measured and the colonies were fixed, stained, photographed and counted.

**EdU assay**

Cells were seeded in 24-well plates at an appropriate density and incubated overnight at 37 ℃. Then the cells were fixed using 4% paraformaldehyde and stained with 50 mM EdU solution for 30 min. DAPI working solution (DAPI final concentration of 10 μg/mL) was used for nuclear staining for 10 min. After incubation, the cells were washed with PBS buffer containing 3% BSA and visualized via confocal microscopy.

**Wound-healing, migration and invasion assays**

For wound-healing assay, cells were plated in 6-well plates at a density of 1×10^6^ cell/well. Cell layers were scratched by a 10-μl pipette tip, and the progression of migration was detected and photo graphed in 24 and 48h. In vitro cell migration and invasion assays were performed using transwell chambers with 8.0 μm polyethylene terephthalate membrane. For the migration assay, 2×10^4^ cells were seeded into the top chambers. For the invasion assay, 2×10^5^ cells were added to top chambers coated with Matrigel. Basic medium was added to the top chambers while complete medium was added to the bottom wells to stimulate migration or invasion. After incubation for 20 h (migration) and 48 h (invasion), cells adhered to the lower surface of the membrane were stained with 0.1% Crystal Violet. Five randomly fields per filter were counted.

**Adhesion experiments**

The six-well plate were coated with 5 μg/mL cohesin overnight at 4 °C. Aspirate the cohesin and add 5,000 cells per well for incubation for 0.5-1 h. Cells were washed three times with PBS, and then fixed with 4% paraformaldehyde for 10 min at room temperature. Then, the cells were incubated with 0.1% crystal violet staining for 15 min and washed with PBS. Finally, taked a picture under a microscope.

**Animal Experiments**

Female BALB/c nude mice purchased from purchased from [SPF Biotechnology Co., Ltd.](https://spfbiotech.com/en) (Beijing, China) were used to generate the xenograft models. MDA-MB-231-Vector, MDA-MB-231-NCOA4 WT, MDA-MB-231-NCOA4 K356R cells, were inoculated subcutaneously at 1×10^6^ /100 μL into mice. After 2 weeks, the tumor size is about 5mm^3^ and DDP (5 mg/kg), OTS186935 (25 mg/kg) or normal saline were injected by intraperitoneal injection every 2 days. After 21 days, the mice were sacrificed with euthanasia and xenografts were collected for subsequent tests.

**Immunohistochemistry, Hematoxylin–Eosin (HE) staining and Prussian blue staining**

Formalin-fixed, paraffin-embedded sections are deparaffinized and rehydrated by xylene and alcohol with different concentration gradients. The antigen is exposed in boiled EDTA buffer and treated with 3% hydrogen peroxide to inactivate endogenous peroxidase activity. Sections are blocked with 10% goat serum for 30 min and then incubated with primary antibody overnight at 4 °C. Then, sections are incubated with secondary antibody for 2 h at room temperature and staining is observed with DAB. Subsequently, the nuclei were dehydrated with hematulan nuclei, gradient ethanol, xylene transparent, neutral gum mounting.

For HE staining, haematoxylin staining solution and eosin staining solution were sequentially utilized to stain the formalin-fixed, paraffin-embedded sections in accordance with the directions.

In terms of Prussian blue staining, the formalin-fixed, paraffin-embedded sections were stained by Perls staining solution and then eosin staining solution.

**ALT, CRE, AST, BUN testing.**

Mice were sacrificed by spinal dislocation, orbital blood was collected, supernatant was obtained by centrifugation, and samples were added for testing according to the kit instructions.

**Pharmacokinetic Study**

In female 6-week-old ICR mice (N=3 per group), OTS186935 was administered intravenously (IV) or orally (PO) at doses of 5.0 mg/kg and 25 mg/kg, respectively. Blood samples (0.05 ml) were collected via the orbital sinus under isoflurane anesthesia before and after dosing, placed in EDTAK2 centrifuge tubes and kept on ice. Centrifuge at 5000 rpm at 4°C for 10 minutes to collect plasma. Sample collection time points: 0, 5 min, 0.25, 0.5, 1, 2, 4, 6, 8, 24 hours. Before analysis, all plasma samples were stored at -80°C. To prepare samples, take 15.0 μL of plasma, add 150 μL of internal standard solution (dexamethasone, 60.0 ng·ml⁻¹), vortex for 60 seconds, then centrifuge for 3 minutes (12,000 rpm). Transfer 100 μL of the supernatant to a 96-well autosampler plate containing an equal volume of water, mix thoroughly by shaking, and analyze by LC-MS/MS, with an injection volume of 10 μL. Data acquisition and control software used is Analyst 1.5.1 (Applied Biosystems).

**Reference**

1.	Wu EL, Cheng X, Jo S, Rui H, Song KC, Dávila-Contreras EM*, et al.* CHARMM-GUI Membrane Builder toward realistic biological membrane simulations. *J Comput Chem* 2014, **35**(27)**:** 1997-2004.

2.	Wang Y, Zhang C, Tang K, Wang X. En route for molecular dynamics simulation of a living cell. *Fundam Res* 2025, **5**(4)**:** 1478-1480.

3.	Van Der Spoel D, Lindahl E, Hess B, Groenhof G, Mark AE, Berendsen HJ. GROMACS: fast, flexible, and free. *J Comput Chem* 2005, **26**(16)**:** 1701-1718.

4.	Huang J, Rauscher S, Nawrocki G, Ran T, Feig M, de Groot BL*, et al.* CHARMM36m: an improved force field for folded and intrinsically disordered proteins. *Nat Methods* 2017, **14**(1)**:** 71-73.

5.	Hess B, Bekker H, Berendsen HJC, Fraaije JGEM. LINCS: A linear constraint solver for molecular simulations. *J Comput Chem* 1997, **18**(12)**:** 1463-1472.

6.	Darden T, York D, Pedersen L. Particle mesh Ewald: An N⋅log(N) method for Ewald sums in large systems. *J Chem Phys* 1993, **98**(12)**:** 10089-10092.

7.	Nosé S. A molecular dynamics method for simulations in the canonical ensemble. *Mol Phys* 1984, **52**(2)**:** 255-268.

8.	Bouysset C, Fiorucci S. ProLIF: a library to encode molecular interactions as fingerprints. *J Cheminform* 2021, **13**(1)**:** 72.

9.	Humphrey W, Dalke A, Schulten K. VMD: Visual molecular dynamics. *J Mol Graph Model* 1996, **14**(1)**:** 33-38.

10.	Delano WL. The PyMOL Molecular Graphics System. 2002; 2002.
